# Ring size changes in the development of class I HDAC inhibitors

**DOI:** 10.1080/14756366.2021.1941920

**Published:** 2021-06-24

**Authors:** Er-Chieh Cho, Chi-Yuan Liu, Di-Wei Tang, Hsueh-Yun Lee

**Affiliations:** a School of Pharmacy, College of Pharmacy, Taipei Medical University, Taipei, Taiwan; b Master Program in Clinical Genomics and Proteomics, College of Pharmacy, Taipei Medical University, Taipei, Taiwan; c Cancer Center, Wan Fang Hospital, Taipei Medical University, Taipei, Taiwan; d Ph.D. Program in Drug Discovery and Development Industry, College of Pharmacy, Taipei Medical University, Taipei, Taiwan

**Keywords:** Thienylbenzamides, ring transformation, HDAC, colon cancer

## Abstract

Five pathways involving different ring structures led to generation of fourteen thienylbenzamides (**7–20**) which display the structure-activity relationships of class I HDAC inhibitors. All the synthesised compounds inhibit HDAC1 and HDAC2 selectively over other isoforms and many inhibit DLD1 and HCT116 cells more effectively than a parent compound. Compounds **8** and **16** inhibit HCT116 cells by activation of the apoptosis pathway.

**ARTICLE HISTORY**
Received 24 March 2021
Revised 31 May 2021
Accepted 8 June 2021

**GRAPHICAL ABSTRACT**

**8**

**16**

| | DLD1 | HCT116 | HDAC1 | HDAC2 |
|---|---|---|---|---|
| **8** | 2.98 μM | 1.01 μM | 31.4 nM | 177 nM |
| **16** | 3.17 μM | 0.77 μM | 34.6 nM | 201 nM |

## 1. Introduction

Epigenetic regulation involves a variety of DNA based processes such as DNA replication, transcription and DNA repair and is critical to cellular development[1–2]. Targeting epigenetic regulators has emerged in recent decades as an attractive strategy for treatment of human diseases. The dysregulation of histone deacetylase (HDAC) activity is linked to neurological syndromes, inflammation and carcinogenesis. HDAC, an epigenetic enzyme, plays an important role in gene expression by removing the acetyl group of acetylated lysine residues on histones and some other proteins[3]. To date, 18 members of HDAC groups have been identified in humans, and can be categorised into four classes (I-IV) depending on their cellular localisation and homologies with yeast. So far, most HDAC inhibitors (HDACi) such as the FDA-approved drugs vorinostat, belinostat and panobinostat are pan-HDACis which non-selectively inhibit class I and II members. Use of pan-HDACis can lead to some side effects, including gastrointestinal syndrome, fatigue, and thrombocytopenia[4–5]. Researchers have begun to focus on the development of HDACis with class or isoform selectivity in order to reduce their adverse effects and raise their therapeutic index. The relationship between each HDAC isoform and human cancer is quite complicated and not yet fully understood; nevertheless, it is certain that HDAC1 and HDAC2 are significantly involved in several types of malignancies and serve as important targets for cancer treatment[6]. HDAC1 is highly expressed in liver cancer cell lines for example, and its inactivation impairs cell cycle transition and causes autophagic cell death[7]. HDAC2 is found in pancreatic ductal adenocarcinoma (PDAC) and confers resistance towards etoposide in PDAC cells[8]. A program in our institute has tried to identify selective inhibitors that target HDAC1/2 preferentially. In general, the structure of an HDACi is divided into a cap region, a linker, and a zinc binding group (ZBG) and modification of each of these three parts is likely to cause a considerable impact on the selectivity of an HDACi[9]. After transforming the hydroxamic acid ZBG on vorinostat for example, into an *o*-aminoanilide, the molecule became inactive towards HDAC6[10]. Further

**CONTACT** Hsueh-Yun Lee hyl@tmu.edu.tw School of Pharmacy, College of Pharmacy, Taipei Medical University, Taipei, Taiwan; Ph.D. Program in Drug Discovery and Development Industry, College of Pharmacy, Taipei Medical University, Taipei, Taiwan
#Contributed equally to this work.

Supplemental data for this article can be accessed here.

studies suggest that structures containing an *o*-aminoanilide as the ZBG generally inhibit class I HDACs. Such inhibitors are exemplified by benzamide compounds **1** (MS-275), **2** (MGCD0103), and **3** (CI-994)[11,12]. In a survey of the literature, we noted that a 14 Å internal cavity, which is located near the catalytic active site, is crucial for the selectivity of HDAC proteins. This internal cavity was first studied by Wang et al.[13] It was hypothesised that this cavity accommodates the acetate by-product of deacetylation and provides it with an exit pathway. Researchers discovered that attached substituents on the *o*-aminoanilide can access the 14 Å internal cavity, and this led to the development of biaryl type benzamides including **4** (Merck60), **5** (MRLB-223) and compound **6**[14–16]. These molecules specifically attained selectivity towards HDAC1 and HDAC2 by adding a small hydrophobic aromatic unit such as a phenyl or thienyl group to the *para* position of the aniline. According to the homology models of HDAC1 and HDAC3 based on the crystal structure of an HDAC8 mutant, the additional aromatic unit is able to occupy the 14 Å internal cavity in HDAC1 but not in HDAC3 due to the difference in the amino acid sequence of the latter (Figure 1).

Ring constriction is a comprehensive strategy in the process of structural optimisation. In our previous study, we utilised this strategy to modify the antitubulin agent ABT751, obtaining furanylazaindoles such as **7** as potent antitubulin agents[17]. In an enzyme complex with compound **4**, the amide group of **4** is located at an open area of the enzyme, which is an area that can accommodate various scaffolds. In order to understand the details of the SAR study and seek structural possibilities, this study applied the strategy of ring transformation to the modification of the benzamide moiety (Figure 2). There are four ring closing pathways envisaged in the study, which respectively give derivatives of indole (**7**, pathway a), indoline-/tetrahydroquinoline (**8–13**, pathway b), pyridine (**14**, pathway c), and lactam-containing moieties (**15–16**, pathway d). In addition, the amide moiety was modified by reversing the motif followed by ring restriction, which provides compounds **17–20**. In addition to the synthetic routes to

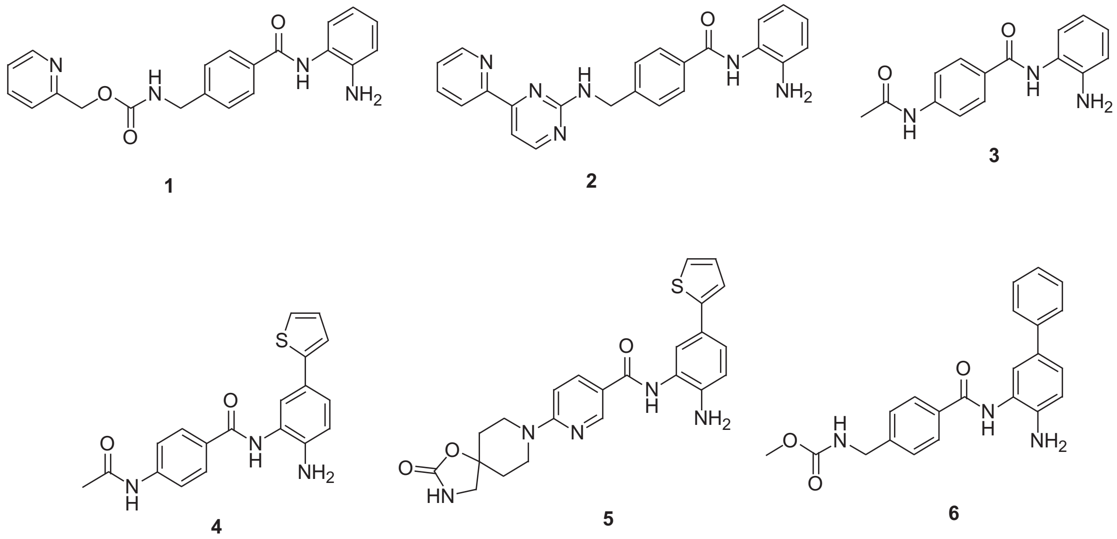

Figure 1. Class I HDAC inhibitors (**1–6**).

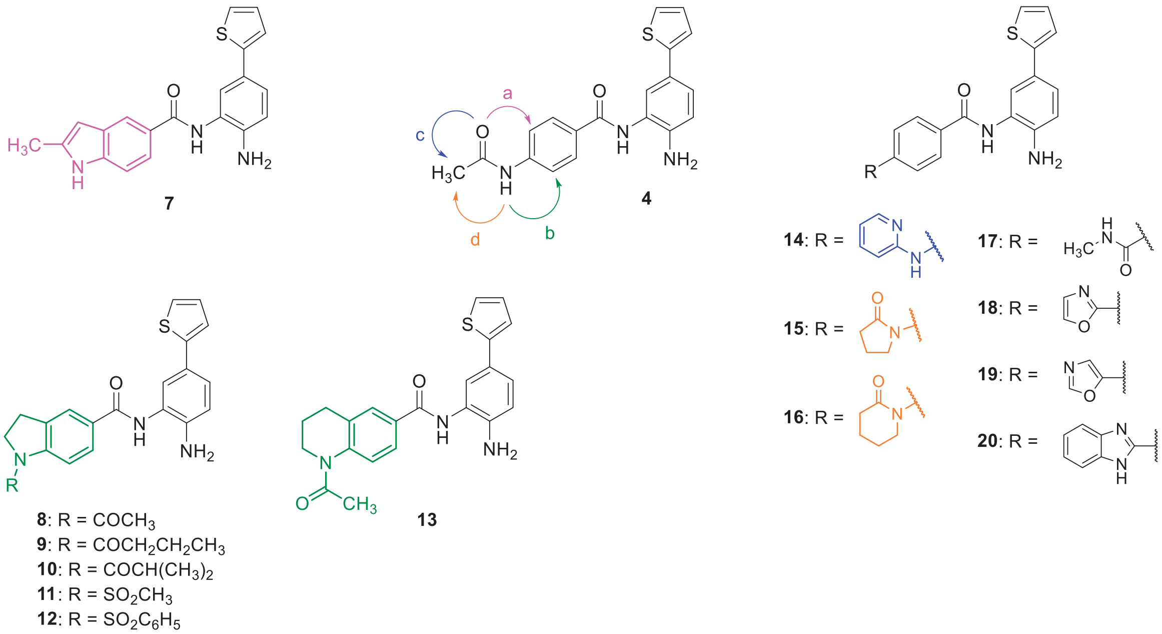

Figure 2. Structural modification and structures of compounds **7–20**.

these designed compounds (**7–20**), an investigation of their biological activity will be examined in this study.

## 2. Results and discussion

### 2.1. Chemistry

In the synthesis of compounds **7–20**, the Boc-masked benzene-1,2-diamine (**25**) is a crucial building block which is obtained from the synthetic route shown in Scheme 1. 2-Nitroaniline (**21**) was brominated with NBS, and the resulting product (**22**) was protected by a Boc group to provide compound **23**. The subsequent Suzuki arylation of **23** with 2-thienylboronic acid gave **24**, which was subjected to reduction of its nitro group to afford compound **25**.

Scheme 2 shows the synthesis of compound **7**. The condensation between 4-methyl-3-nitrobenzoate (**26**) and DMF-DMA yielded the enamine **27**, which was then treated with acetyl chloride and subsequent hydrolysis generated compound **28**. The resulting product was then reacted with iron powder in the presence of acetic acid to generate the indole-5-carboxylate (**29**) which was subjected to hydrolysis to obtain the corresponding indole-5-carboxylic acid (**30**). The amidation of **30** with **25**, with the assistance of HATU gave compound **31** which was subjected to TFA-medicated Boc-deprotection to afford compound **7**.

Scheme 3 shows the synthesis of indoline-containing compounds (**8–12**). The N-substitution of methyl indoline-5-carboxylate (**32**) was carried out by the reaction with acetic anhydride, butyryl chloride, isobutyryl chloride, methanesulfonyl chloride or benzenesulfonyl chloride affording **33a–33e**. The subsequent hydrolysis of the ester group in **33a–33e** using LiOH yielded the corresponding carboxylic acids (**34a–34e**). The subsequent amidation of **34a–34e** with **25** gave compounds **35a–35e** which were subjected to TFA-mediated deprotection to obtain compounds **8–12**.

Production of a tetrahydroquinoline was also achieved, and is shown in Scheme 4. With a catalytic amount of 10% Pd/C, reduction of quinoline-6-carboxylic acid (**36**) by ammonium formate was carried out in refluxing MeOH to obtain the acid (**37**). The resulting product was treated with acetyl chloride to yield **38**

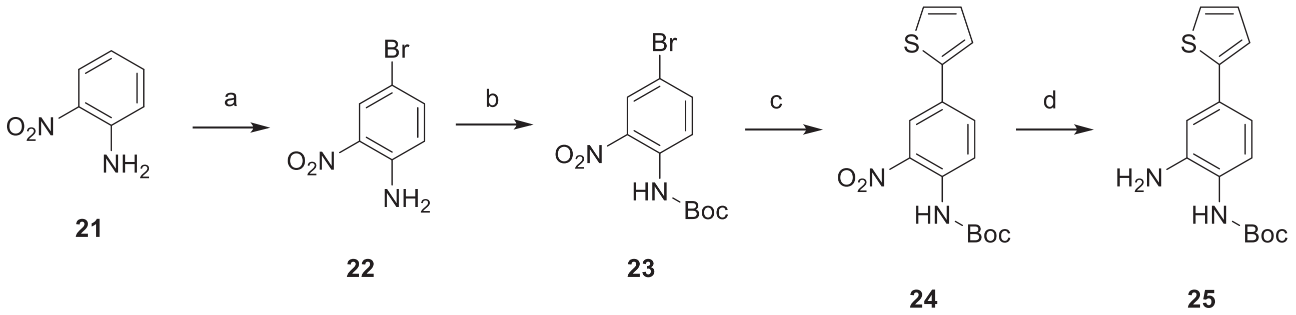

**Scheme 1.** Synthetic approach to compound **25**. Reagents and conditions: (a) NBS, acetic acid, 0 °C; (b) i. $Boc_2O$, DMAP, THF, rt; ii. 5N $NaOH_{(aq)}$, THF, 70 °C; (c) 2-thienylboronic acid, $Pd(PPh_3)_4$, $P(o\text{-}tol)_3$, $K_2CO_3$, $H_2O$, DME, reflux; (d) $H_2$, 10% Pd/C, MeOH, rt.

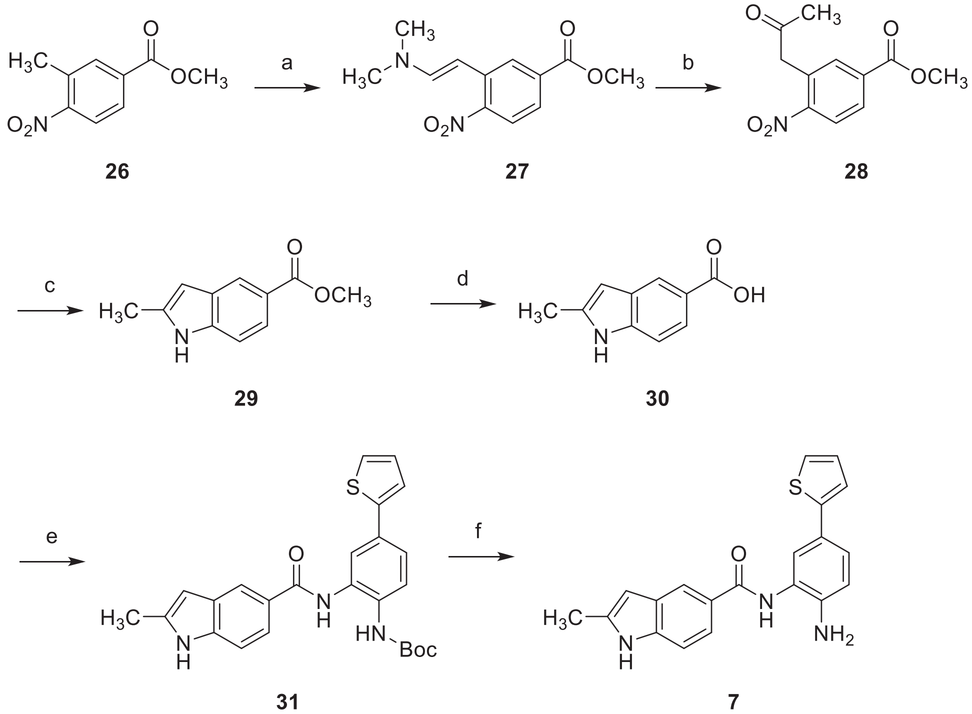

**Scheme 2.** Synthetic approach to compound **7**. Reagents and conditions: (a) DMF-DMA, reflux; (b) i. acetyl chloride, pyridine, DCM, rt; ii. p-dioxane, $H_2O$, reflux; (c) Fe powder, $CH_3COOH$, 90 °C; (d) NaOH, $H_2O$, MeOH, reflux; (e) **25**, HATU, DIPEA, DMF, 70 °C; (f) TFA, DCM, rt.

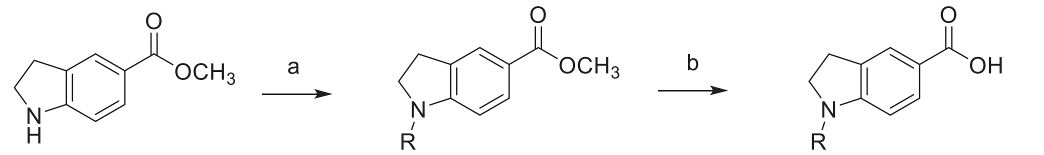

**32**

**33a**: R = acetyl
**33b**: R = butyryl
**33c**: R = isobutyryl
**33d**: R = methanesulfonyl
**33e**: R = benzenesulfonyl

**34a**: R = acetyl
**34b**: R = butyryl
**34c**: R = isobutyryl
**34d**: R = methanesulfonyl
**34e**: R = benzenesulfonyl

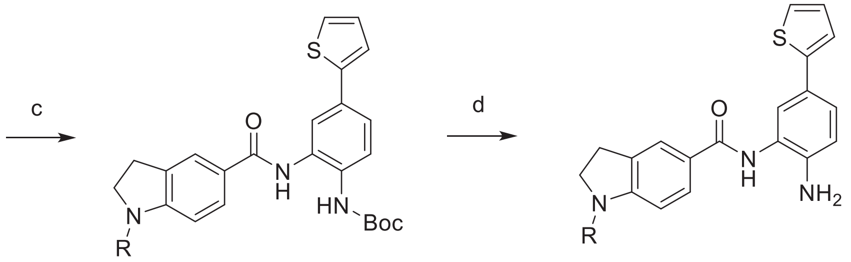

**35a**: R = acetyl
**35b**: R = butyryl
**35c**: R = isobutyryl
**35d**: R = methanesulfonyl
**35e**: R = benzenesulfonyl

**8**: R = acetyl
**9**: R = butyryl
**10**: R = isobutyryl
**11**: R = methanesulfonyl
**12**: R = benzenesulfonyl

**Scheme 3.** Synthetic approach to compounds **8–12**. Reagents and conditions: (a) for **33a**: acetic anhydride, TEA, DCM, 130 °C; for **33b**: butyryl chloride, TEA, DCM, 0 °C; for **33c**: isobutyryl chloride, TEA, DCM, 0 °C; for **33d**: methanesulfonyl chloride; pyridine, 0 °C; for **33e**: benzenesulfonyl chloride; pyridine, rt; (b) 1N $LiOH_{(aq)}$, THF, rt; (c) **25**, HATU, DIPEA, DMF, 60 °C; (d) TFA, DCM, rt.

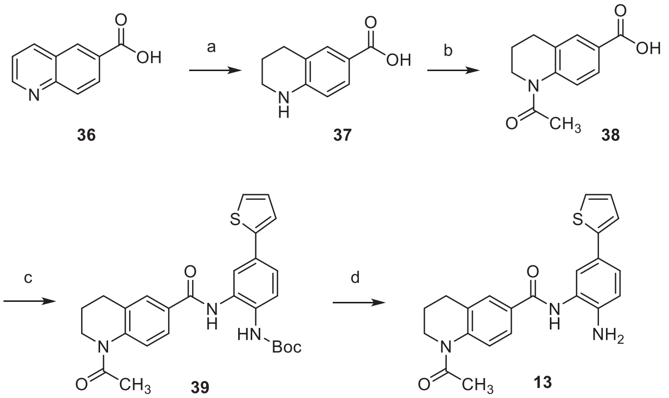

**Scheme 4.** Synthetic approach to compound **13**. Reagents and conditions: (a) ammonium formate, 10% Pd/C, MeOH, reflux; (b) acetyl chloride, TEA, DCM, rt; (c) **25**, HATU, DIPEA, DMF, rt, 6 h, 50 °C; (d) TFA, DCM, rt.

which was subjected to the synthetic routes to the anticipated 4-thienyl-2-aminobenzamide shown in Scheme 1 to obtain compound **13**.

Scheme 5 shows the synthetic route to compounds **14–20**. The reaction of methyl 4-aminobenzoate (**40a**) with 2-bromopyridine gave the corresponding benzoate (**41a**). Methyl 4-bromobenzoate (**40b**) underwent Buchwald-Hartwig amination with 2-pyrrolidone and 2-piperidone yielding compounds **41b** and **41c**, respectively. The reverse amide moiety was obtained from the reaction of **40c** with methylamine hydrochloride with assistance of EDC·HCl, generating compound **41d**. Compound **40c**, upon amidation with aminoacetaldehyde dimethyl acetal followed by treatment with $MeSO_3H$ and phosphorus pentoxide ($P_4O_{10}$), yielded the oxazole-substituted compound (**41e**). Methyl 4-formylbenzoate (**40d**) was reacted with toluenesulfonylmethyl isocyanide (TosMIC) under the conditions of the van Leusen reaction to afford compound **41f**, which is a regioisomer of **41e**. Alternatively, **40d** was reacted with *o*-phenylenediamine in the presence of $Na_2S_2O_4$ to produce the benzimidazole ester (**41g**). All synthesised substituted benzoates (**41a–41g**) underwent hydrolysis by LiOH or NaOH, which generated the corresponding carboxylic acids **42a–42g**. The resulting products were converted into the final products (**14–20**) through routes similar to those in Scheme 1.

### *2.2. Biological evaluation*

#### *2.2.1.* In vitro *cell growth inhibitory activity*

All synthetic compounds (**7–20**) were examined for their cellular activity against two colorectal cancer cells, DLD1 and HCT116. The $IC_{50}$ values shown in Table 1 were determined after DLD1 and HCT116 cells were exposed to each tested compound for 48 h or 72 h. $IC_{50}$ curves of these compounds in DLD1 and HCT116 cells were analysed and shown in the Supplementary Figure 1. The results in the first column reveal that most of the compounds tested, with the exception of **10**, **17**, and **18**, showed better DLD1 inhibitory activity than the thiophenylaniline (**4**). In the third column it can be seen that most of tested compounds exhibited comparable or weaker activity against HCT116 cells, when compared with **4**, but compounds **8**, **16**, **17**, and **18** inhibit the growth of HCT116 cells with $IC_{50}$ values of 4.13, 0.34, 6.12, and 1.84 μM, respectively therefore displaying improved HCT116 inhibitory activity. In order to understand the influence of exposure time on antiproliferative activity, compounds that showed activity against both DLD1 and HCT116 cells were selected and tested for their cellular activity in a 72 h exposure. The result shows that most of the selected compounds displayed slightly better potency

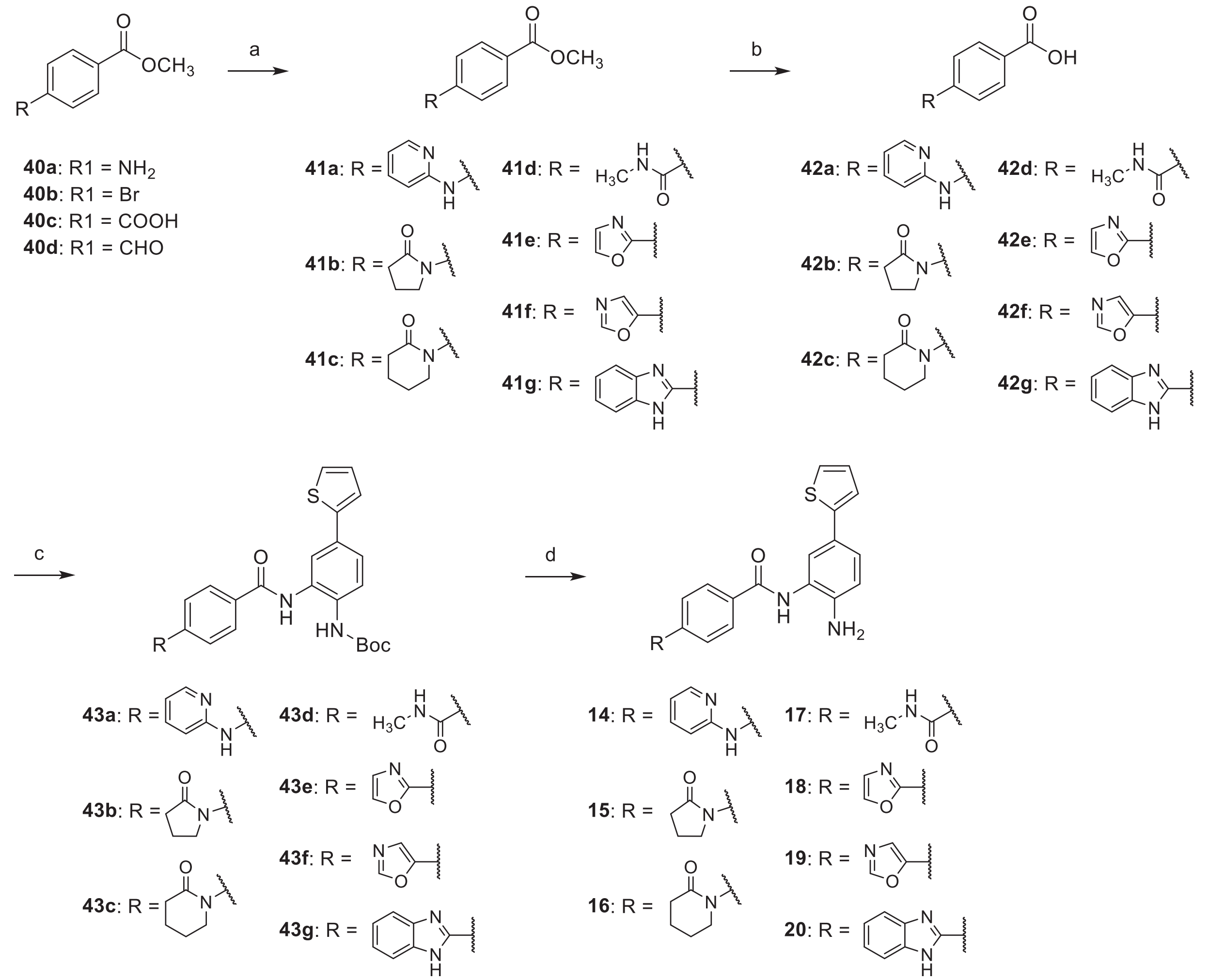

**Scheme 5.** Synthetic approach to compounds **14–20**. Reagents and conditions: (a) for compound **41a**: **40a**, 2-bromopyridine, 140 °C; for compound **41b**: **40b**, 2-pyrrolidone, $Pd_2(dba)_3$, xantphos, $Cs_2CO_3$, p-dioxane, reflux; for compound **41c**: **40b**, 2-piperidone, $Pd_2(dba)_3$, xantphos, $Cs_2CO_3$, p-dioxane, reflux; for compound **41d**: **40c**, methylamine hydrochloride, EDC·HCl, DMAP, DMF, rt; for compound **41e**: i. **40c**, aminoacetaldehyde dimethyl acetal, EDC·HCl, NMM, DCM, rt; ii. $MeSO_3H$, $P_4O_{10}$, $N_2$, 140 °C; for compound **41f**: **40d**, TosMIC, $K_2CO_3$, MeOH, reflux; for compound **41g**: **40d**, o-phenylenediamine, $Na_2S_2O_4$, DMF, 70 °C; (b) for compounds **41a**, **41d**, **41g**: 1N $LiOH_{(aq)}$, p-dioxane, rt; for compounds **41b**, **41c**, **41e**, **41f**: 1N $NaOH_{(aq)}$, MeOH, rt; (c) **25**, HATU, DIPEA, DMF, rt; (d) TFA, DCM, rt.

**Table 1.** Inhibitory activity ($IC_{50}$, μM[a]) against colorectal cancer cells after the treatment of tested compounds for 48 h or 72 h.

| | DLD1 | | HCT116 | |
|---|---|---|---|---|
| Compd | 48 h | 72 h | 48 h | 72 h |
| **7** | 18.68 ± 0.73 | ND[b] | 16.31 ± 2.04 | ND |
| **8** | 2.87 ± 0.20 | 2.98 ± 0.20 | 4.13 ± 0.14 | 1.01 ± 0.42 |
| **9** | 18.47 ± 2.45 | 2.45 ± 0.49 | 25.63 ± 1.41 | 2.46 ± 0.39 |
| **10** | 29.58 ± 23.6 | 5.17 ± 3.13 | 15.56 ± 1.92 | 2.61 ± 0.42 |
| **11** | 10.03 ± 0.64 | 1.64 ± 0.07 | 13.67 ± 1.36 | 2.30 ± 0.37 |
| **12** | 3.82 ± 0.15 | 1.39 ± 0.58 | 36.54 ± 0.54 | 2.72 ± 0.43 |
| **13** | 3.29 ± 0.12 | 4.83 ± 0.50 | 14.55 ± 0.81 | 1.21 ± 0.02 |
| **14** | 3.98 ± 0.14 | 3.16 ± 0.27 | 13.04 ± 0.40 | 1.31 ± 0.07 |
| **15** | 7.66 ± 1.09 | 3.33 ± 0.30 | 11.13 ± 1.71 | 1.44 ± 0.93 |
| **16** | 3.76 ± 0.94 | 3.17 ± 0.11 | 6.12 ± 0.86 | 0.77 ± 0.01 |
| **17** | 26.28 ± 1.99 | ND | 0.34 ± 0.14 | ND |
| **18** | 31.32 ± 1.58 | ND | 1.84 ± 0.76 | ND |
| **19** | 14.84 ± 2.41 | ND | 12.02 ± 0.73 | ND |
| **20** | 3.55 ± 0.99 | ND | 24.88 ± 0.09 | ND |
| **4** | 29.28 ± 3.41 | 6.20 ± 0.26 | 13.6 ± 0.68 | 1.05 ± 0.10 |

[a]The $IC_{50}$ was estimated using GraphPad Prism 7 software and shown as mean ± SD from at last three independent experiments. [b]ND: not determined.

than **4** towards DLD1 cells, while only compound **16** showed better HCT116 inhibitory activity than compound **4**. Moreover, in order to evaluate the cancer targeting specificity of these compounds, we applied two non-cancerous human cell lines, 293 T and IMR90 cells, to address this issue. Cells were treated with ten of the compounds with great cancer cell inhibition activity for 48 h, analysed by MTT assay, and then calculated for $IC_{50}$. Our results showed that the $IC_{50}$ of these compounds in non-cancerous cells were much higher than that in cancer cells, suggested that our compounds exhibit high cancer targeting specificity. In summary, the synthesised compounds derived from ring transformation of the amide moiety act more rapidly than the parent compound, and the selected compounds (**8–16**) display a slightly better DLD1 inhibitory activity than **4**.

Due to the distinct differences in the results from the 48 h experiment, the following discussion is focussed on these results. The acetyl-substituted indoline molecule (**8**, from path b in Figure 2) showed approximately a 10-fold and 3-fold rate increase in cytotoxicity against DLD1 and HCT116 cells, respectively when compared to compound **4**. After 48 h treatment of **8**, the compound inhibited the growth of DLD1 and HCT116 cells with $IC_{50}$ values of 2.87 and 4.13 μM, respectively. Comparison of **8** with **9** and **10** suggests that the decrease of activity can probably be attributed to the bulky nature of the N-substituents in the indoline scaffold. The expansion of ring size from indoline (**8**) to tetrahydroquinoline (**13**) indeed led to a slight reduction of HCT116 inhibition.

Table 2. Inhibitory activity ($IC_{50}$, M) of compounds against HDAC1, HDAC2, HDAC6 and HDAC8.

| Compd | HDAC1 | HDAC2 | HDAC6 | HDAC8 |
|---|---|---|---|---|
| **7** | $7.80 \times 10^{-8}$ | $2.60 \times 10^{-7}$ | $>1.0 \times 10^{-5}$ | $>1.0 \times 10^{-5}$ |
| **8** | $3.14 \times 10^{-8}$ | $1.77 \times 10^{-7}$ | $>1.0 \times 10^{-5}$ | $>1.0 \times 10^{-5}$ |
| **9** | $9.27 \times 10^{-8}$ | $4.58 \times 10^{-7}$ | $>1.0 \times 10^{-5}$ | $>1.0 \times 10^{-5}$ |
| **10** | $4.62 \times 10^{-8}$ | $2.18 \times 10^{-7}$ | $>1.0 \times 10^{-5}$ | $>1.0 \times 10^{-5}$ |
| **11** | $3.07 \times 10^{-8}$ | $1.52 \times 10^{-7}$ | $>1.0 \times 10^{-5}$ | $>1.0 \times 10^{-5}$ |
| **12** | $1.11 \times 10^{-7}$ | $5.35 \times 10^{-7}$ | $>1.0 \times 10^{-5}$ | $>1.0 \times 10^{-5}$ |
| **13** | $3.88 \times 10^{-8}$ | $2.29 \times 10^{-7}$ | $>1.0 \times 10^{-5}$ | $>1.0 \times 10^{-5}$ |
| **14** | $5.84 \times 10^{-8}$ | $1.88 \times 10^{-7}$ | $>1.0 \times 10^{-5}$ | $>1.0 \times 10^{-5}$ |
| **15** | $6.71 \times 10^{-8}$ | $3.15 \times 10^{-7}$ | $>1.0 \times 10^{-5}$ | $>1.0 \times 10^{-5}$ |
| **16** | $3.46 \times 10^{-8}$ | $2.01 \times 10^{-7}$ | $>1.0 \times 10^{-5}$ | $>1.0 \times 10^{-5}$ |
| **17** | $3.25 \times 10^{-8}$ | $8.53 \times 10^{-8}$ | $>1.0 \times 10^{-5}$ | $>1.0 \times 10^{-5}$ |
| **18** | $5.51 \times 10^{-8}$ | $2.83 \times 10^{-7}$ | $>1.0 \times 10^{-5}$ | $>1.0 \times 10^{-5}$ |
| **19** | $3.68 \times 10^{-8}$ | $1.63 \times 10^{-7}$ | $>1.0 \times 10^{-5}$ | $>1.0 \times 10^{-5}$ |
| **20** | $8.94 \times 10^{-8}$ | $3.61 \times 10^{-7}$ | $>1.0 \times 10^{-5}$ | $>1.0 \times 10^{-5}$ |
| **4** | $3.11 \times 10^{-8}$ | $1.53 \times 10^{-7}$ | $>1.0 \times 10^{-5}$ | $>1.0 \times 10^{-5}$ |
| Trichostatin A | $1.51 \times 10^{-8}$ | $3.20 \times 10^{-8}$ | $1.45 \times 10^{-9}$ | $8.71 \times 10^{-7}$ |

Compounds **15** and **16**, designed from path d in Figure 2 displayed better activity than **4**, and also showed that the six-membered lactam (**16**) is favoured for cellular activity. Compounds **17–20**, obtained through a reverse amide route have inconsistent inhibitory activities on DLD1 and HCT116 cells. Compounds **17** and **18** for instance, show remarkable antiproliferative activity against HCT116 cells but are inactive towards DLD1 cells. Notably, among compounds **7–20**, compound **17**, with an $IC_{50}$ value of 0.34 μM is the most active inhibitor of HCT116 cells.

### *2.2.2. HDAC isoform inhibitory activity*

Table 2 indicates the inhibitory activity against HDAC isoforms. Compounds with cellular activity were selected for this enzymatic activity, and their results are compared with those from compound **4**. With exception of **12**, all other tested compounds display HDAC1 and HDAC2 inhibitory activity comparable to that of **4**, which reveals that this series of compounds retain class I HDAC isoform activity over other HDAC isoforms. This result also reveals that with the presence of a thienylbenzamide moiety the tested compounds retain class I HDAC inhibitory activity, and the modification of surface recognition area provokes only a very slight change in the HDAC isoform inhibition. In the first column it can be seen that HDAC1 is more sensitive to compounds having bulkier substitution. For example, **9**, **12**, and **20** exhibit weaker HDAC1 inhibitory activity than others, with $IC_{50}$ values of 92.7, 111.0, and 89.4 nM, respectively. Despite **9**, **12**, and **20** also result in weaker HDAC2 inhibitory activity, the extent of influence is less than that shown in the first column. In addition to the influence of bulkiness of substituents on the inhibition of HDAC subtypes, the orientation of amide moiety is also observed. For instance, **17** possessing a reverse amide motif shows comparable HDAC1 activity to that of **4** and results in 2-fold increase of HDAC2 potency than **4**. Compound **17** exhibits the highest inhibitory activity against HDAC1 and HDAC2, with $IC_{50}$ values of 32.5 and 85.3 nM, respectively.

### *2.2.3. Flow cytometry analysis*

Biological assays were carried out to investigate the inhibitory and regulatory mechanisms of compounds **8** and **16** towards cancer cells, because **8** and **16** show potent activity against both DLD1 and HCT116 in a 48 h exposure (Table 1). Firstly, flow cytometry analysis was performed. HCT116 colon cancer cells were treated with compounds **8** or **16** at different concentrations and harvested at various time points for analysis (Figure 3(A)). The cell cycle profile was analysed, and the sub-G1 population represents apoptotic cells. The results showed that there was significant (∼20%) induction of the sub-G1 population in both groups, one treated at 1 μM for 48 h, the other at 0.2 μM for 72 h (Figure 3(A)), suggesting that both compounds can inhibit cancer cells by activating the apoptosis pathway. A dose dependent effect was observed with both compounds (Figure 3(A)). The morphology of cells in each group was monitored and recorded as a control in the flow cytometry (Figure 3(B)).

### *2.2.4. Western blot analysis*

A Western blot assay was also performed. HCT116 cells were treated with compounds **8** and **16** at different concentrations and harvested at the 48 h time point for analysis. The signal of acetylated histone H3 induced in cells being treated (Figure 4(A)), suggested that these two compounds exhibited class I HDACs inhibition capacity. There was also slight induction of the acetylated α-tubulin signal, which suggested that these two compounds might exhibit a certain level of HDAC6 inhibitory capacity (Figure 4(A)). The data showed that under the treatment of compounds **8** and **16**, the signal of apoptotic markers, including the PARP cleaved form and PUMA, and cell cycle arrest marker p21 were all induced (Figure 4(A)). This suggested that compounds **8** and **16** are able to activate an apoptosis pathway and affect the cell cycle regulation in colon cancer cells. The signals from Western blot assay were quantified and analysed by image J software compared to GAPDH, the loading control, and are shown in Figure 4(B).

### *2.2.5. Docking study of compounds 4, 8, and 16*

Figure 5 demonstrates the binding modes of compounds **4** (yellow), **8** (purple), and **16** (green) in the binding site of HDAC2 (PDB ID: 5IX0), which was defined using the Glide module of Schrödinger Maestro. The result indicates that compounds (**8** and **16**) designed with ring closure, have similar spatial alignments to that of **4** in the binding site. The 2-aminobenzamide moiety of **4**, **8**, and **16** forms H-bonds with His145 and His146 of the target enzyme. The amide motif of **4**, **8**, and **16** interacts with Gly154 residue *via* H-bonding. The similar interactions of **4**, **8**, and **16** explain their HDAC isoform inhibitory activity shown in Table 2.

## 3. Conclusion

This study utilised a ring restriction strategy to modify the amide motif of reported HDAC inhibitors, and led to various heterocycle-containing replacements, including indole (**7**), indolines (**8–12**)/tetrahydroquinoline (**13**), pyridine (**4**), lactams (**15–16**), and oxazoles (**18–19**). Many of synthesised compounds show a faster effect in antiproliferative assays than that of **4**, and displayed distinct antiproliferative activity after a 48 h treatment. The structure-activity relationship study indicated that N-acetylindoline derivative (**8**) and the piperidinone-containing compound (**16**) exhibit marked antiproliferative activity against DLD1 and HCT116 cells. After 48 h treatment, **8** has $IC_{50}$ values of 2.87 and 4.13 μM towards DLD1 and HCT116 cells and **16** has $IC_{50}$ values of 3.76 and 6.12 μM towards DLD1 and HCT116 cells. The anticancer activity of these compounds stems from their activation of the apoptosis pathway. Compounds **8** and **16** show HDAC1 and HDAC2 inhibitory activity comparable to that of **4**, and have the similar spatial alignment and interaction in the active site of HDAC enzyme. The results cited above indicate that the strategy of ring closure contributes to the decrease in the response time, and is probably due to the change of their physicochemical properties. Our work reports a

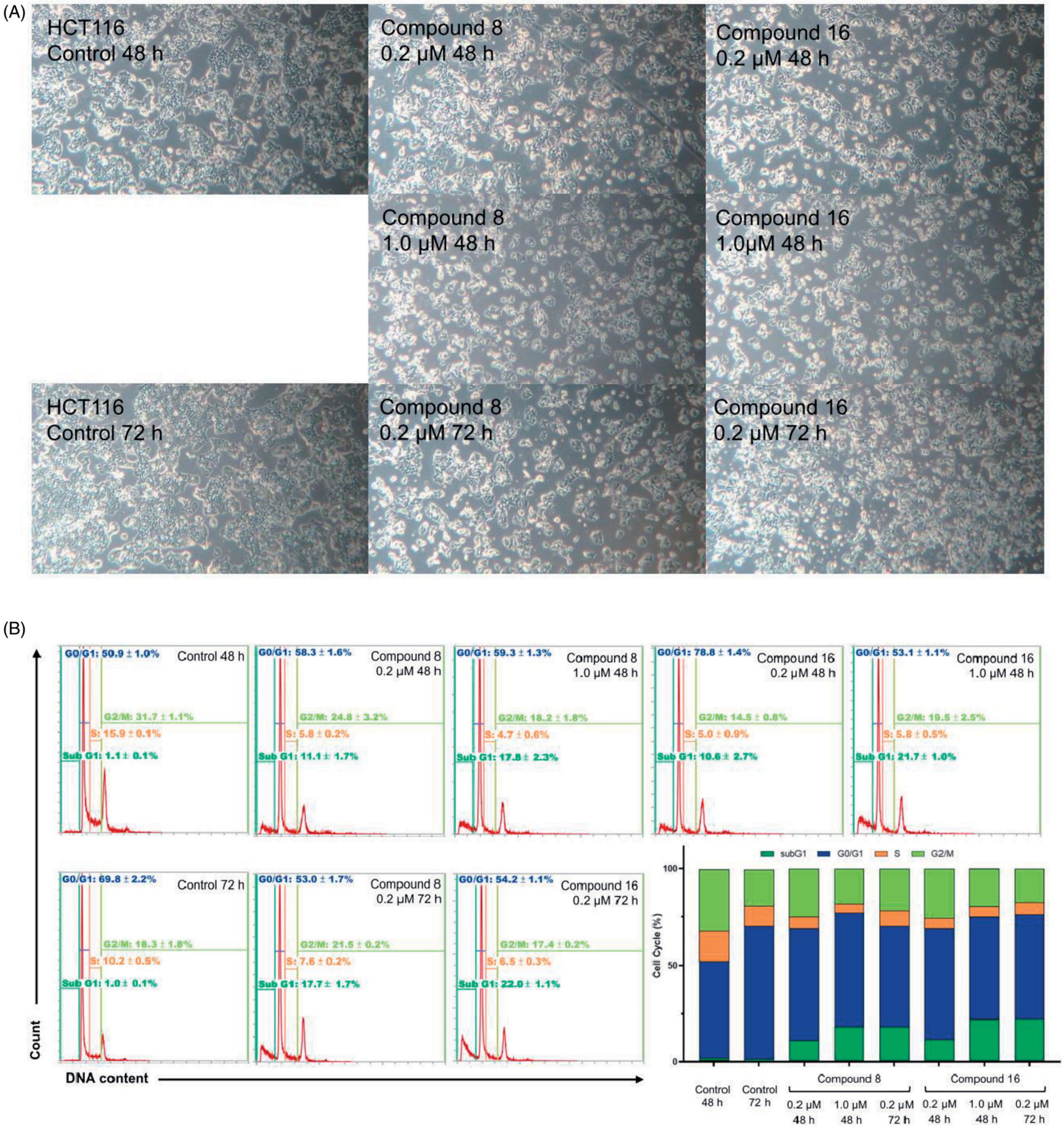

**Figure 3.** Bright-field microscopy of untreated/treated HCT116 cells (A) and flow cytometric analysis (B). Compounds **8** and **16** induced subG1 cell accumulation of HCT116 in a dose- and time-dependent manner. HCT116 cells were treated with **8** (0.2 μM, 1.0 μM) and **16** (0.2 μM, 1.0 μM) for indicated times, then the cell cycle distribution and DNA content were analysed by flow cytometry. Quantitative data were evaluated on the flow cytometric histograms and are shown as mean ± SD.

detailed study of the structure-activity relationships at the cap region and could benefit the development of class I HDAC inhibitors with concerning pharmcokinetic features.

## 4. Experimental section

### 4.1. Chemistry

Nuclear magnetic resonance spectra were obtained with Bruker DRX-300 spectrometer operating at 300 MHz and Agilent DD2 600 MHz NMR operating at 600 MHz, with chemical shifts reported in parts per million (ppm, $\delta$) downfield from TMS, an internal standard. High-resolution mass spectra (HRMS) were measured with a JEOL (JMS-700) electron impact (EI) mass spectrometer. Purity of the final compounds was determined using a Hitachi 2000 series HPLC system using C-18 column (Agilent ZORBAX Eclipse XDB-C18 5 μm. 4.6 mm $\times$ 150 mm) and was found to be $\geq$ 95% in all cases. Flash column chromatography was carried out using silica gel (Merck Kieselgel 60, No. 9385, 230–400 mesh ASTM). All reactions were done under an atmosphere of dry nitrogen.

#### 4.1.1. N-(2-Amino-5-(thiophen-2-yl)phenyl)-2-methyl-1H-indole-5-carboxamide (7)

Trifluoroactic acid (TFA, 0.6 ml) was added to a solution of compound **31** (0.34 g, 0.76 mmol) in dichloromethane (DCM, 8 ml) and

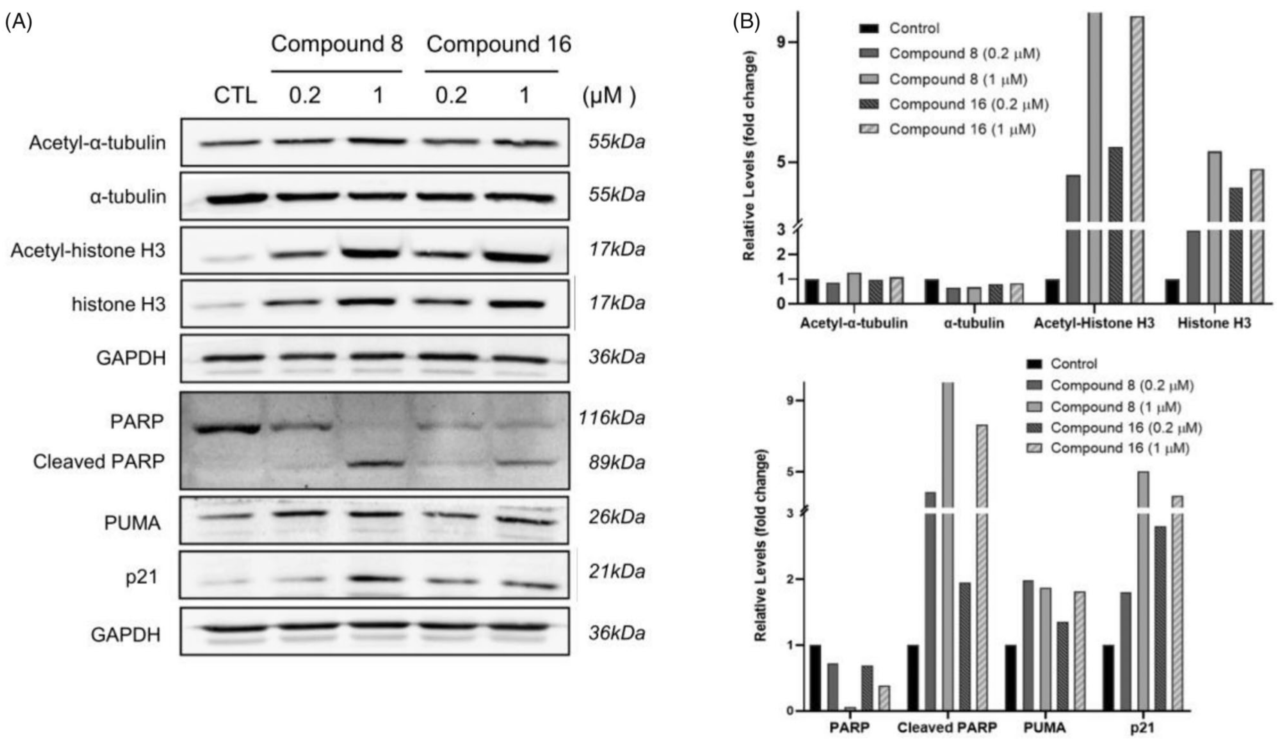

**Figure 4.** After pre-treatment, compounds **8** and **16** suppressed the cell growth effect of HCT116 cells. (A) Western blot analysis of acetylated α-tubulin, α-tubulin, acetylated histone H3, histone H3, PARP, PUMA, and p21 after treatment for 48 h with increasing doses of **8** and **16** in HCT116 cells. GAPDH was used as a loading control. (B) Graphical representation of the protein expression intensity normalised with corresponding loading control.

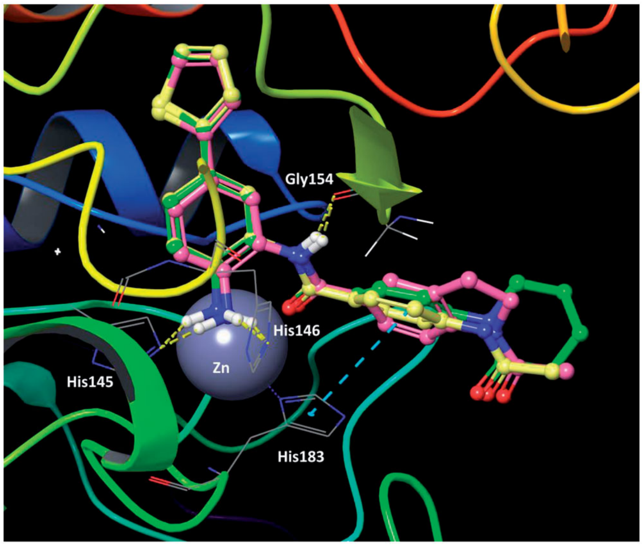

**Figure 5.** Overlay of compounds **4** (yellow), **8** (purple), and **16** (green) in the binding site of HDAC2 (PDB ID: 5IX0).

stirred at room temperature (rt) for 6 h. After completion of the reaction, the mixture was neutralised with sat. $NaHCO_{3(aq)}$ and extracted with EtOAc. The organic layer was dried over anhydrous $MgSO_4$, and the solvent was removed. The residue was purified by flash column chromatography (DCM/MeOH = 250/1) to afford the title compound (0.14 g, 53%); mp 106 °C (dec). $^1$H NMR (300 MHz, DMSO-$d_6$) $\delta$ 11.21 (s, 1H), 9.61 (s, 1H), 8.17 (s, 1H), 7.70 (dd, $J$ = 8.4, 1.8 Hz, 1H), 7.53 (d, $J$ = 2.1 Hz, 1H), 7.38–7.33 (m, 2H), 7.29 (dd, $J$ = 8.4, 2.1 Hz, 1H), 7.25 (dd, $J$ = 3.6, 1.2 Hz, 1H), 7.05 (dd, $J$ = 5.1, 3.6 Hz, 1H), 6.83 (d, $J$ = 8.1 Hz, 1H), 6.27 (s, 1H), 5.12 (s, 2H), 2.42 (s, 3H). $^{13}$C NMR (75 MHz, DMSO-$d_6$) $\delta$ 166.6, 144.4, 142.8, 137.9, 137.2, 128.2, 128.0, 125.0, 124.4, 123.7, 123.5, 123.2, 122.4, 121.0, 120.2, 119.5, 116.5, 110.0, 100.2, 13.4. HRMS (ESI) for $C_{20}H_{18}N_3OS$ (M + H$^+$) calcd 348.1165, found 338.1172.

#### *4.1.2. 1-Acetyl-N-(2-amino-5-(thiophen-2-yl)phenyl)indoline-5-carboxamide (8)*

The title compound was obtained in 51% overall yield from compound **35a** in a manner similar to that described for the preparation of **7**: mp 219 °C. $^1$H NMR (300 MHz, DMSO-$d_6$) $\delta$ 9.63 (s, 1H), 8.10 (d, $J$ = 8.4 Hz, 1H), 7.88–7.84 (m, 2H), 7.46 (d, $J$ = 1.8 Hz, 1H), 7.38–7.34 (m, 1H), 7.29 (dd, $J$ = 8.7, 2.1 Hz, 1H), 7.26–7.22 (m, 1H), 7.05 (dd, $J$ = 5.1, 3.6 Hz, 1H), 6.81 (d, $J$ = 8.4 Hz, 1H), 5.12 (s, 2H), 4.17 (t, $J$ = 8.4 Hz, 2H), 3.21 (t, $J$ = 8.4 Hz, 2H), 2.20 (s, 3H). $^{13}$C NMR (150 MHz, DMSO-$d_6$) $\delta$ 169.1, 165.0, 145.6, 144.2, 142.9, 131.9, 129.0, 128.2, 127.6, 124.5, 123.8, 123.8, 123.7, 123.2, 122.3, 121.0, 116.4, 114.8, 48.6, 27.1, 24.0. HRMS (ESI) for $C_{21}H_{20}N_3O_2S$ (M + H$^+$) calcd 378.1271, found 378.1276.

#### *4.1.3. N-(2-Amino-5-(thiophen-2-yl)phenyl)-1-butyrylindoline-5-carboxamide (9)*

The title compound was obtained in 99% overall yield from compound **35b** in a manner similar to that described for the preparation of **7**: $^1$H NMR (300 MHz, DMSO-$d_6$) $\delta$ 9.62 (s, 1H), 8.14 (d, $J$ = 8.4 Hz, 1H), 7.88–7.80 (m, 2H), 7.46 (d, $J$ = 2.1 Hz, 1H), 7.35 (dd, $J$ = 5.1, 1.2 Hz, 1H), 7.28 (dd, $J$ = 8.4, 2.4 Hz, 1H), 7.24 (dd, $J$ = 3.6, 0.9 Hz, 1H), 7.04 (dd, $J$ = 5.1, 3.6 Hz, 1H), 6.81 (d, $J$ = 8.4 Hz, 1H), 5.11 (s, 2H), 4.16 (t, $J$ = 8.4 Hz, 2H), 3.20 (t, $J$ = 8.4 Hz, 2H), 2.50–2.40 (m, 2H), 1.69–1.58 (m, 2H), 0.95 (t, $J$ = 7.5 Hz, 3H). $^{13}$C NMR (150 MHz, DMSO-$d_6$) $\delta$ 171.4, 165.00, 145.7, 144.2, 142.9, 131.8, 128.8, 128.2, 127.6, 124.5, 123.8, 123.7, 123.6, 123.2, 122.3, 121.0, 116.4, 114.8, 47.8, 39.1, 27.1, 17.2, 13.7. HRMS (ESI) for $C_{23}H_{24}N_3O_2S$ (M + H$^+$) calcd 406.1589, found 406.1592.

#### 4.1.4. N-(2-Amino-5-(thiophen-2-yl)phenyl)-1-isobutyrylindoline-5-carboxamide (10)

The title compound was obtained in 92% overall yield from compound **35c** in a manner similar to that described for the preparation of **7**: $^{1}$H NMR (300 MHz, DMSO-$d_6$) $\delta$ 9.63 (s, 1H), 8.16 (d, $J$ = 7.5 Hz, 1H), 7.89–7.84 (m, 2H), 7.46 (d, $J$ = 7.5 Hz, 1H), 7.35 (dd, $J$ = 5.4, 1.2 Hz, 1H), 7.29 (dd, $J$ = 8.1, 2.1 Hz, 1H), 7.24 (dd, $J$ = 3.6, 1.2 Hz, 1H), 7.07–7.03 (m, 1H), 6.81 (d, $J$ = 8.1 Hz, 1H), 5.11 (s, 2H), 4.24 (t, $J$ = 8.4 Hz, 2H), 3.23–3.19 (m, 2H), 2.90–2.85 (m, 1H), 1.12 (d, $J$ = 6.6 Hz, 6H). $^{13}$C NMR (150 MHz, DMSO-$d_6$) $\delta$ 175.5, 164.9, 145.8, 144.2, 142.9, 132.0, 128.9, 128.1, 127.5, 124.4, 123.8, 123.7, 123.6, 123.1, 122.3, 120.9, 116.4, 115.2, 47.8, 32.5, 30.6, 27.1, 18.9. HRMS (ESI) for $C_{23}H_{24}N_3O_2S$ $(M + H^+)$ calcd 406.1589, found 406.1588.

#### 4.1.5. N-(2-Amino-5-(thiophen-2-yl)phenyl)-1-(methylsulfonyl)indoline-5-carboxamide (11)

The title compound was obtained in 70% overall yield from compound **35d** in a manner similar to that described for the preparation of **7**: $^{1}$H NMR (300 MHz, DMSO-$d_6$) $\delta$ 9.64 (s, 1H), 7.93–7.88 (m, 2H), 7.45 (d, $J$ = 2.1 Hz, 1H), 7.37–7.32 (m, 2H), 7.29 (dd, $J$ = 8.4, 2.1 Hz, 1H), 7.23 (dd, $J$ = 3.6, 0.9 Hz, 1H), 7.07–7.03 (m, 1H), 6.81 (d, $J$ = 8.4 Hz, 1H), 5.12 (s, 2H), 4.02 (t, $J$ = 8.4 Hz, 2H), 3.19 (t, $J$ = 8.4 Hz, 2H), 3.07 (s, 3H). $^{13}$C NMR (150 MHz, DMSO-$d_6$) $\delta$ 164.8, 144.7, 144.2, 142.9, 131.8, 129.4, 128.2, 128.1, 125.2, 123.9, 123.8, 123.5, 123.1, 122.2, 120.9, 116.3, 112.1, 50.2, 34.6, 27.0. HRMS (ESI) for $C_{20}H_{20}N_3O_3S_2$ $(M + H^+)$ calcd 414.0946, found 414.0948.

#### 4.1.6. N-(2-Amino-5-(thiophen-2-yl)phenyl)-1-(phenylsulfonyl)indoline-5-carboxamide (12)

The title compound was obtained in 93% overall yield from compound **35e** in a manner similar to that described for the preparation of **7**: $^{1}$H NMR (300 MHz, DMSO-$d_6$) $\delta$ 9.71 (s, 1H), 7.91–7.86 (m, 3H), 7.80 (s, 1H), 7.75–7.68 (m, 1H), 7.64–7.55 (m, 3H), 7.45 (d, $J$ = 2.1 Hz, 1H), 7.38 (dd, $J$ = 5.1, 1.2 Hz, 1H), 7.32 (dd, $J$ = 5.1, 2.4 Hz, 1H), 7.26 (dd, $J$ = 3.6, 1.2 Hz, 1H), 7.05 (dd, $J$ = 5.1, 3.6 Hz, 1H), 6.87 (d, $J$ = 8.4 Hz, 1H), 4.01 (d, $J$ = 8.4 Hz, 2H), 3.03 (d, $J$ = 8.4 Hz, 2H). $^{13}$C NMR (150 MHz, DMSO-$d_6$) $\delta$ 164.8, 144.0, 143.8, 140.5, 135.9, 133.9, 132.0, 129.5, 128.2, 128.1, 127.1, 125.2, 124.6, 123.9, 123.8, 123.7, 123.6, 121.4, 117.5, 112.9, 50.3, 26.8. HRMS (ESI) for $C_{25}H_{22}N_3O_3S_2$ $(M + H^+)$ calcd 476.1103, found 476.1104.

#### 4.1.7. 1-Acetyl-N-(2-amino-5-(thiophen-2-yl)phenyl)-1,2,3,4-tetrahydroquinoline-6-carboxamide (13)

The title compound was obtained in 50% overall yield from compound **39** in a manner similar to that described for the preparation of **7**: mp 107–109 °C. $^{1}$H NMR (300 MHz, DMSO-$d_6$) $\delta$ 9.67 (s, 1H), 7.84 (s, 1H), 7.81 (d, $J$ = 8.4 Hz, 1H), 7.73–7.60 (m, 1H), 7.46 (d, $J$ = 2.1 Hz, 1H), 7.35 (dd, $J$ = 5.1, 1.2 Hz, 1H), 7.30 (dd, $J$ = 8.4, 2.1 Hz, 1H), 7.24 (dd, $J$ = 3.6, 1.2 Hz, 1H), 7.05 (dd, $J$ = 5.4, 3.9 Hz, 1H), 6.81 (d, $J$ = 8.4 Hz, 1H), 5.13 (s, 2H), 3.72 (t, $J$ = 6.3 Hz, 2H), 2.80 (t, $J$ = 6.3 Hz, 2H), 2.23 (s, 3H), 1.91 (p, $J$ = 6.6 Hz, 2H). $^{13}$C NMR (150 MHz, DMSO-$d_6$) $\delta$ 169.6, 165.0, 144.2, 143.0, 141.4, 131.3, 129.9, 128.2, 125.4, 123.9, 123.9, 123.5, 123.2, 122.3, 121.0, 116.4, 44.1, 26.6, 23.4, 23.3. HRMS (ESI) for $C_{22}H_{22}N_3O_2S$ $(M + H^+)$ calcd 392.1427, found 392.1432.

#### 4.1.8. N-(2-Amino-5-(thiophen-2-yl)phenyl)-4-(pyridin-2-ylamino)-benzamide (14)

The title compound was obtained in 41% overall yield from compound **43a** in a manner similar to that described for the preparation of **7**: mp 202 °C. $^{1}$H NMR (300 MHz, DMSO-$d_6$) $\delta$ 9.56 (s, 1H), 9.41 (s, 1H), 8.25–8.22 (m, 1H), 7.96 (d, $J$ = 8.7 Hz, 2H), 7.84 (d, $J$ = 8.7 Hz, 2H), 7.66–7.59 (m, 1H), 7.50 (d, $J$ = 2.4 Hz, 1H), 7.35 (dd, $J$ = 5.1, 1.2 Hz, 1H), 7.30 (dd, $J$ = 8.4, 2.1 Hz, 1H), 7.25 (dd, $J$ = 3.6, 1.2 Hz, 1H), 7.05 (dd, $J$ = 5.1, 3.6 Hz, 1H), 6.92 (d, $J$ = 8.4 Hz, 1H), 6.86–6.80 (m, 2H), 5.13 (s, 2H). $^{13}$C NMR (75 MHz, DMSO-$d_6$) $\delta$ 165.1, 155.3, 147.3, 144.8, 144.3, 142.9, 137.5, 128.8, 128.2, 125.5, 123.9, 123.8, 123.7, 123.2, 122.4, 121.0, 116.5, 116.4, 115.2, 111.5. HRMS (ESI) for $C_{22}H_{19}N_4OS$ $(M + H^+)$ calcd 387.1274, found 387.1276.

#### 4.1.9. N-(2-Amino-5-(thiophen-2-yl)phenyl)-4-(2-oxopyrrolidin-1-yl)benzamide (15)

The title compound was obtained in 55% overall yield from compound **43b** in a manner similar to that described for the preparation of **7**: mp 223 °C. $^{1}$H NMR (300 MHz, DMSO-$d_6$) $\delta$ 9.77 (s, 1H), 8.05 (d, $J$ = 8.7 Hz, 2H), 7.80 (d, $J$ = 9.0 Hz, 2H), 7.48 (d, $J$ = 2.1 Hz, 1H), 7.35 (dd, $J$ = 5.1, 1.2 Hz, 1H), 7.29 (dd, $J$ = 8.4, 2.1 Hz, 1H), 7.25 (dd, $J$ = 3.6, 1.2 Hz, 1H), 7.05 (dd, $J$ = 5.1, 3.6 Hz, 1H), 6.82 (d, $J$ = 8.4 Hz, 1H), 5.16 (s, 2H), 3.90 (t, $J$ = 7.2 Hz, 2H), 2.55 (t, $J$ = 7.8 Hz, 2H), 2.15–2.04 (m, 2H). $^{13}$C NMR (150 MHz, DMSO-$d_6$) $\delta$ 174.4, 164.8, 144.3, 143.0, 142.2, 129.2, 128.6, 128.2, 123.9, 123.8, 123.6, 123.2, 122.3, 121.0, 118.2, 116.4, 48.0, 32.4, 17.3. HRMS (ESI) for $C_{21}H_{20}N_3O_2S$ $(M + H^+)$ calcd 378.1271, found 378.1275.

#### 4.1.10. N-(2-Amino-5-(thiophen-2-yl)phenyl)-4-(2-oxopiperidin-1-yl)benzamide (16)

The title compound was obtained in 42% overall yield from compound **43c** in a manner similar to that described for the preparation of **7**: mp 97 °C (dec). $^{1}$H NMR (300 MHz, $CDCl_3$) $\delta$ 8.29 (s, 1H), 7.90 (d, $J$ = 8.4 Hz, 2H), 7.53 (d, $J$ = 2.1 Hz, 1H), 7.37–7.32 (m, 3H), 7.20–7.17 (m, 2H), 7.03 (dd, $J$ = 5.1, 3.6 Hz, 1H), 6.83 (d, $J$ = 8.7 Hz, 1H), 3.66 (t, $J$ = 6.0 Hz, 2H), 2.55 (t, $J$ = 6.0 Hz, 2H), 2.00–1.86 (m, 4H). $^{13}$C NMR (150 MHz, $CDCl_3$) $\delta$ 170.5, 165.3, 146.5, 144.4, 140.7, 132.1, 128.5, 128.0, 126.4, 126.2, 125.1, 124.8, 123.7, 123.2, 122.1, 118.7, 51.4, 33.1, 23.6, 21.4. HRMS (ESI) for $C_{22}H_{22}N_3O_2S$ $(M + H^+)$ calcd 392.1427, found 392.1432.

#### 4.1.11. N1-(2-Amino-5-(thiophen-2-yl)phenyl)-N4-methylterephthalamide (17)

The title compound was obtained in 88% overall yield from compound **43d** in a manner similar to that described for the preparation of **7**: mp 252 °C. $^{1}$H NMR (300 MHz, DMSO-$d_6$) $\delta$ 9.83 (s, 1H), 8.59 (d, $J$ = 4.5 Hz, 1H), 8.07 (d, $J$ = 8.1 Hz, 2H), 7.95 (d, $J$ = 8.4 Hz, 2H), 7.48 (d, $J$ = 1.8 Hz, 1H), 7.35 (dd, $J$ = 5.1, 1.2 Hz, 1H), 7.31 (dd, $J$ = 8.4, 2.4 Hz, 1H), 7.25 (dd, $J$ = 3.6, 0.9 Hz, 1H), 7.05 (dd, $J$ = 5.1, 3.6 Hz, 1H), 6.82 (d, $J$ = 8.4 Hz, 1H), 5.18 (s, 2H), 2.82 (d, $J$ = 4.5 Hz, 3H). $^{13}$C NMR (75 MHz, DMSO-$d_6$) $\delta$ 165.9, 165.0, 144.2, 143.2, 136.9, 136.7, 128.2, 127.9, 127.0, 124.1, 124.1, 123.2, 123.1, 122.2, 121.0, 116.3, 26.3. HRMS (ESI) for $C_{19}H_{18}N_3O_2S$ $(M + H^+)$ calcd 352.1114, found 352.1120.

### 4.1.12. N-(2-Amino-5-(thiophen-2-yl)phenyl)-4-(oxazol-2-yl)benzamide (18)

The title compound was obtained in 88% overall yield from compound **43e** in a manner similar to that described for the preparation of **7**: mp 209–211 °C. $^1$H NMR (300 MHz, DMSO-$d_6$) $\delta$ 9.88 (s, 1H), 8.30 (d, $J = 0.6$ Hz, 1H), 8.17 (d, $J = 8.4$ Hz, 2H), 8.12 (d, $J = 8.4$ Hz, 2H), 7.49 (d, $J = 2.1$ Hz, 1H), 7.46 (d, $J = 0.6$ Hz, 1H), 7.36 (dd, $J = 5.1$, 1.2 Hz, 1H), 7.31 (dd, $J = 8.4$, 2.1 Hz, 1H), 7.25 (dd, $J = 3.6$, 1.2 Hz, 1H), 7.05 (dd, $J = 5.1$, 3.6 Hz, 1H), 6.82 (d, $J = 8.4$ Hz, 1H), 5.20 (s, 2H). $^{13}$C NMR (75 MHz, DMSO-$d_6$) $\delta$ 164.8, 160.2, 144.2, 143.2, 140.7, 136.0, 129.3, 128.9, 128.8, 128.2, 125.7, 124.2, 124.1, 123.2, 123.1, 122.2, 121.0, 116.4. HRMS (ESI) for $C_{20}H_{16}N_3O_2S$ $(M + H^+)$ calcd 362.0958, found 362.0964.

### 4.1.13. N-(2-Amino-5-(thiophen-2-yl)phenyl)-4-(oxazol-5-yl)benzamide (19)

The title compound was obtained in 69% overall yield from compound **43f** in a manner similar to that described for the preparation of **7**: mp 209 °C. $^1$H NMR (300 MHz, DMSO-$d_6$) $\delta$ 9.82 (s, 1H), 8.53 (s, 1H), 8.12 (d, $J = 8.4$ Hz, 2H), 7.90–7.86 (m, 3H), 7.48 (d, $J = 2.1$ Hz, 1H), 7.35 (dd, 5.1, 1.2 Hz, 1H), 7.31 (dd, $J = 8.4$, 2.1 Hz, 1H), 7.25 (dd, $J = 3.6$, 0.9 Hz, 1H), 7.05 (dd, $J = 5.1$, 3.6 Hz, 1H), 6.82 (d, $J = 8.4$ Hz, 1H), 5.18 (s, 2H). $^{13}$C NMR (150 MHz, DMSO-$d_6$) $\delta$ 164.8, 152.4, 149.9, 144.2, 143.1, 134.2, 130.0, 128.7, 128.2, 124.1, 124.0, 123.8, 123.5, 123.2, 123.2, 122.3, 121.0, 116.4. HRMS (ESI) for $C_{20}H_{16}N_3O_2S$ $(M + H^+)$ calcd 362.0958, found 362.0962.

### 4.1.14. N-(2-Amino-5-(thiophen-2-yl)phenyl)-4-(1H-benzo[d]imidazol-2-yl)benzamide(20)

The title compound was obtained in 60% overall yield from compound **43g** in a manner similar to that described for the preparation of **7**: mp 356 °C (dec). $^1$H NMR (300 MHz, DMSO-$d_6$) $\delta$ 9.97 (s, 1H), 8.34 (d, 2H), 8.24 (d, 2H), 7.75–7.70 (m, 2H), 7.53 (d, J = 2.1 Hz, 1H), 7.41–7.33 (m, 4H), 7.28 (dd, J = 3.6, 0.9 Hz, 1H), 7.07 (dd, J = 5.1, 3.6 Hz, 1H), 6.89 (d, J = 8.4 Hz, 1H). $^{13}$C NMR (150 MHz, DMSO-$d_6$) $\delta$ 164.8, 149.5, 144.0, 141.6, 136.5, 136.5, 129.9, 128.8, 128.3, 126.9, 124.2, 124.0, 123.9, 123.6, 123.3, 121.4, 117.2, 114.9. HRMS (ESI) for $C_{24}H_{19}N_4OS$ $(M + H^+)$ calcd 411.1274, found 411.1277.

### 4.1.15. 4-Bromo-2-nitroaniline (22)

NBS (4.83 g, 27.14 mmol) was added portionwise to a solution of 2-nitroaniline (**21**, 3.75 g, 27.17 mmol) in AcOH (24 ml) in an ice bath, and stirred at 0 °C for 9 min. The mixture was then poured into ice-cold water, and the suspension was filtered to obtain the title compound as an orange solid (5.52 g, 94%). $^1$H NMR (300 MHz, DMSO-$d_6$) $\delta$ 8.06 (d, $J = 2.4$ Hz, 1H), 7.57 (s, 2H), 7.53 (dd, $J = 9.0$, 2.4 Hz, 1H), 6.99 (d, $J = 9.0$ Hz, 1H).

### 4.1.16. Tert-Butyl (4-bromo-2-nitrophenyl)carbamate (23)

A mixture of **22** (5.52 g, 25.44 mmol), $Boc_2O$ (16.8 g, 76.98 mmol), and DMAP (0.31 g, 2.54 mmol) in THF (50 ml) was stirred at rt for 2 days, and then the solvent was removed *in vacuo* to obtain a dark purple oil which was redissolved in THF (50 ml), treated with 5 N $NaOH_{(aq)}$ (16 ml, 80 mmol), and heated to 70 °C for 15 h. After completion of the reaction, the solvent was removed under reduced pressure. Water was added water to the residue, which was then extracted with DCM, and dried over anhydrous $MgSO_4$. The solvent was then concentrated to afford the title compound (8.00 g, 99%) which was used in next step without further purification. $^1$H NMR (300 MHz, DMSO-$d_6$) $\delta$ 9.67 (s, 1H), 8.12 (d, $J = 2.4$ Hz, 1H), 7.86 (dd, $J = 9.0$, 2.4 Hz, 1H), 7.59 (d, $J = 8.7$ Hz, 1H), 1.44 (s, 9H).

### 4.1.17. Tert-Butyl (2-nitro-4-(thiophen-2-yl)phenyl)carbamate (24)

A mixture of **23** (1.23 g, 3.88 mmol), 2-thienylboronic acid (0.66 g, 5.16 mmol), $Pd(PPh_3)_4$ (0.27 g, 0.23 mmol), P(o-tol)$_3$ (0.36 g, 1.17 mmol), $K_2CO_3$ (1.64 g, 11.87 mmol), $H_2O$ (7 ml) and DME (20 ml) was stirred at reflux for 4.5 h. After completion of the reaction, the mixture was filtered through celite and washed with EtOAc. Water was added to the filtrate, and the two layers were separated. The organic layer was dried over anhydrous $MgSO_4$, and the solvent was removed. The crude material was purified by flash column chromatography (Hex/DCM = 5/1) to afford the title compound as a yellow solid (1.02 g, 82%). $^1$H NMR (300 MHz, DMSO-$d_6$) $\delta$ 9.64 (s, 1 H), 8.14 (d, $J = 2.4$ Hz, 1H), 7.94 (dd, $J = 8.7$, 2.1 Hz, 1H), 7.69 (d, $J = 8.7$ Hz, 1H), 7.64–7.60 (m, 2H), 7.17 (dd, $J = 5.1$, 3.6 Hz, 1 H), 1.45 (s, 9H).

### 4.1.18. Tert-Butyl (2-amino-4-(thiophen-2-yl)phenyl)carbamate (25)

A catalytic amount of 10% Pd/C was added to a suspension of **24** (1.02 g, 3.18 mmol) in MeOH (10 ml), and the mixture was stirred at rt for 4 h under a hydrogen balloon. After completion of the reaction, the mixture was filtered through celite, and the solvent was removed *in vacuo* to afford the title compound as a light yellow solid (0.92 g, 99%). $^1$H NMR (300 MHz, DMSO-$d_6$) $\delta$ 8.33 (s, 1H), 7.44 (dd, $J = 5.1$, 1.2 Hz, 1H), 7.29 (dd, $J = 3.6$, 1.2 Hz, 1H), 7.26 (d, $J = 8.4$ Hz, 1H), 7.08 (dd, $J = 5.1$, 3.6 Hz, 1H), 6.97 (d, $J = 2.1$ Hz, 1H), 6.84 (d, $J = 8.4$, 2.1 Hz, 1H), 5.00 (s, 2H), 1.46 (s, 9H).

### 4.1.19. Methyl (E)-3-(2-(dimethylamino)vinyl)-4-nitrobenzoate (27)

A mixture of **26** (5 g, 25.63 mmol) and DMF-DMA (8 ml, 60.09 mmol) was stirred at reflux for 31 h under an inert atmosphere. After completion of the reaction, the solvent was removed *in vacuo*, and the residue was recrystallized from MeOH to yield the title compound as a dark purple solid (3.9 g, 61%). $^1$H NMR (300 MHz, DMSO-$d_6$) $\delta$ 8.16 (d, $J = 1.8$ Hz, 1H), 7.84 (d, $J = 8.4$ Hz, 1H), 7.5 (d, $J = 13.2$ Hz, 1H), 7.43 (dd, $J = 8.4$, 1.8 Hz, 1H), 5.51 (d, $J = 13.5$ Hz, 1H), 3.88 (s, 3H), 2.91 (s, 6H).

### 4.1.20. Methyl 4-nitro-3-(2-oxopropyl)benzoate (28)

Acetyl chloride (1.44 ml, 20.36 mmol) was added dropwise to a solution of **27** (3.6 g, 14.39 mmol) and pyridine (1.85 ml, 22.96 mmol) in DCM (60 ml). After stirring at rt for 24 h, water was added to the mixture and the organic layer was collected and concentrated. Water (9.6 ml) and dioxane (18 ml) were added to the residue, which was then heated to reflux for 15 h. After cooling down, the dioxane was then evaporated under reduced pressure, and the residue was extracted with EtOAc. The organic layer was collected, dried over anhydrous $MgSO_4$, concentrated, and purified by flash column chromatography (Hex/EtOAc = 5/1) to afford the title compound (2.2 g, 64%). $^1$H NMR (300 MHz, $CDCl_3$) $\delta$ 8.14 (d, $J = 8.4$ Hz, 1H), 8.09 (dd, $J = 8.4$, 1.8 Hz, 1H), 7.95 (d, $J = 1.8$ Hz, 1H), 4.18 (s, 2H), 3.96 (s, 1H), 2.33 (s, 1H).

### 4.1.21. Methyl 2-methyl-1H-indole-5-carboxylate (29)

A mixture of **28** (2.8 g, 11.80 mmol) and Fe powder (6.53 g, 116.61 mmol) in AcOH (30 ml) was stirred at 90 °C for 12.5 h. After cooling down, the reaction mixture was filtered through celite, neutralised with sat. $NaHCO_{3(aq)}$, and extracted with EtOAc. The organic layer was dried over anhydrous $MgSO_4$, and the solvent was removed. The crude material was purified by flash column chromatography (Hex/EtOAc = 6/1) to afford the title compound (1.83 g, 82%). $^1$H NMR (300 MHz, DMSO-$d_6$) $\delta$ 11.30 (s, 1H), 8.11–8.09 (m, 1 H), 7.64 (dd, $J$ = 8.4, 1.8 Hz, 1H), 7.33 (dt, $J$ = 8.4, 0.6 Hz, 1H), 6.27–6.26 (m, 1H), 3.81 (s, 3H), 2.39 (d, $J$ = 0.6 Hz, 3H).

#### *4.1.22. 2-Methyl-1H-indole-5-carboxylic acid (30)*

NaOH (0.5 g, 12.50 mmol) and $H_2O$ (6 ml) were added to a solution of **29** (0.6 g, 3.17 mmol) in MeOH (15 ml). The mixture was stirred at rt for 1.5 h, then acidified with 3 N $HCl_{(aq)}$. The resulting suspension was filtered to afford the title compound (0.55 g, 98%). $^1$H NMR (300 MHz, DMSO-$d_6$) $\delta$ 12.29 (s, 1H), 11.25 (s, 1H), 8.07 (d, $J$ = 1.8 Hz, 1H), 7.62 (dd, $J$ = 8.4, 1.8 Hz, 1H), 7.30 (d, $J$ = 8.4 Hz, 1H), 6.25–6.23 (m, 1H), 2.39 (d, $J$ = 0.9 Hz, 3H).

#### *4.1.23. Tert-Butyl (2-(2-methyl-1H-indole-5-carboxamido)-4-(thiophen-2-yl)phenyl) carbamate (31)*

DIPEA (2.48 ml, 14.24 mmol) was added to a solution of **30** (0.5 g, 2.85 mmol), **25** (1.13 g, 3.89 mmol), and HATU (2.17 g, 5.71 mmol) in DMF (14 ml), and the mixture was stirred at 70 °C for 20 h. The reaction was quenched with water, followed by extraction with EtOAc. The organic layer was dried over anhydrous $MgSO_4$, concentrated, and purified by flash column chromatography to afford the title compound (0.48 g, 38%). $^1$H NMR (300 MHz, DMSO-$d_6$) $\delta$ 11.27 (s, 1H), 9.82 (s, 1H), 8.78 (s, 1H), 8.11 (d, $J$ = 1.5 Hz, 1H), 7.89 (d, $J$ = 2.1 Hz, 1H), 7.66 (dd, $J$ = 8.7, 1.5 Hz, 1H), 7.56 (d, $J$ = 8.7 Hz, 1H), 7.53 (dd, $J$ = 5.1, 1.2 Hz, 1H), 7.49 (dd, $J$ = 8.4, 2.1 Hz, 1H), 7.46 (dd, 3.6, 1.2 Hz, 1H), 7.37 (d, $J$ = 8.7 Hz, 1H), 7.13 (dd, $J$ = 5.1, 3.6 Hz, 1H), 6.27 (s, 1H), 2.42 (d, $J$ = 0.6 Hz, 3H), 1.47 (s, 9H).

#### *4.1.24. Methyl 1-acetylindoline-5-carboxylate (33a)*

A mixture of methyl indoline-5-carboxylate (**32**, 1.0 g, 5.64 mmol) and acetic anhydride ($Ac_2O$, 3.5 ml) was stirred at 130 °C for 2 h. After completion of the reaction, the mixture was poured into ice-cold water, and the resulting suspension was filtered. The solid was recrystallized from MeOH to afford the title compound (1.08 g, 87%). $^1$H NMR (300 MHz, DMSO-$d_6$) $\delta$ 8.10 (d, $J$ = 9 Hz, 1H), 7.82–7.78 (m, 2H), 4.15 (t, $J$ = 8.7 Hz, 2H), 3.81 (s, 3H), 3.18 (t, $J$ = 8.4 Hz, 2H), 2.19 (s, 3H).

#### *4.1.25. Methyl 1-(methylsulfonyl)indoline-5-carboxylate (33d)*

The title compound was obtained from compound **32** in a manner similar to that described for the preparation of **33a**: $^1$H NMR (300 MHz, $CDCl_3$) $\delta$ 7.93–7.86 (m, 2H), 7.40 (d, $J$ = 8.4 Hz, 1H), 4.04 (t, $J$ = 8.7 Hz, 2H), 3.88 (s, 3H), 3.18 (t, $J$ = 8.7 Hz, 2H), 2.92 (s, 3H).

#### *4.1.26. Methyl 1-(methyl-($\lambda^1$-oxidanyl)-(phenyl)sulfinyl)indoline-5-carboxylate (33e)*

The title compound was obtained from compound **32** in a manner similar to that described for the preparation of **33a**: $^1$H NMR (300 MHz, DMSO-$d_6$) $\delta$ 7.90–7.81 (m, 3H), 7.74–7.67 (m, 2H), 7.63–7.54 (m, 3H), 3.98 (t, $J$ = 8.4 Hz, 2H), 3.79 (s, 3H), 3.02 (t, $J$ = 8.4 Hz, 2H).

#### *4.1.27. 1-Acetylindoline-5-carboxylic acid (34a)*

1N $LiOH_{(aq)}$ (2.65 ml) was added to a solution of compound **33a** (0.2 g, 0.91 mmol) in THF (5 ml), and the mixture was stirred at rt for 5 h. After completion of the reaction, water was added to the mixture which was then acidified with 3 N $HCl_{(aq)}$ to pH $\approx$ 4. The resulting suspension was filtered to yield the title compound (0.14 g, 75%). $^1$H NMR (300 MHz, DMSO-$d_6$) $\delta$ 8.07 (d, 8.7 Hz, 1H), 7.79–7.76 (m, 2H), 4.14 (t, $J$ = 8.7 Hz, 2H), 3.17 (t, $J$ = 8.7 Hz, 2H), 2.18 (s, 3H).

#### *4.1.28. 1-Butyrylindoline-5-carboxylic acid (34b)*

The title compound was obtained from compound **33b** in a manner similar to that described for the preparation of **34a**: $^1$H NMR (300 MHz, DMSO-$d_6$) $\delta$ 12.61 (s, 1H), 8.11 (d, $J$ = 8.7 Hz, 1H), 7.79–7.75 (m, 2H), 4.13 (t, $J$ = 8.4 Hz, 2H), 3.16 (t, $J$ = 8.4 Hz, 2H), 2.45 (t, $J$ = 7.5 Hz, 2H), 1.67–1.54 (m, 2H), 0.94 (t, $J$ = 7.5 Hz, 3H).

#### *4.1.29. 1-Isobutyrylindoline-5-carboxylic acid (34c)*

The title compound was obtained from compound **33c** in a manner similar to that described for the preparation of **34a**: $^1$H NMR (300 MHz, DMSO-$d_6$) $\delta$ 12.63 (s, 1H), 8.13 (d, $J$ = 7.8 Hz, 1H), 7.79–7.75 (m, 2H), 4.21 (t, $J$ = 8.4 Hz, 2H), 3.18 (t, $J$ = 8.4 Hz, 2H), 2.90–2.80 (m, 1H), 1.10 (d, $J$ = 6.6 Hz, 6H).

#### *4.1.30. 1-(Methylsulfonyl)indoline-5-carboxylic acid (34d)*

The title compound was obtained from compound **33d** in a manner similar to that described for the preparation of **34a**: $^1$H NMR (300 MHz, DMSO-$d_6$) $\delta$ 12.71 (s, 1H), 7.84–7.80 (m, 2H), 7.33–7.29 (m, 1H), 4.00 (t, $J$ = 8.4 Hz, 2H), 3.15 (t, $J$ = 8.4 Hz, 2H), 3.06 (s, 3H).

#### *4.1.31. 1-(Phenylsulfonyl)indoline-5-carboxylic acid (34e)*

The title compound was obtained from compound **33e** in a manner similar to that described for the preparation of **34a**: $^1$H NMR (300 MHz, DMSO-$d_6$) $\delta$ 12.72 (s, 1H), 7.89–7.85 (m, 2H), 7.80 (dd, $J$ = 8.4, 1.8 Hz, 1H), 7.74–7.67 (m, 2H), 7.63–7.56 (m, 2H), 7.53 (d, $J$ = 8.4 Hz, 1H), 3.97 (t, $J$ = 8.4 Hz, 2H), 3.00 (t, $J$ = 8.4 Hz, 2H).

#### *4.1.32. Tert-Butyl (2-(1-acetylindoline-5-carboxamido)-4-(thiophen-2-yl)phenyl) carbamate (35a)*

The title compound was obtained in 90% overall yield from compound **34a** in a manner similar to that described for the preparation of **31**: $^1$H NMR (300 MHz, DMSO-$d_6$) $\delta$ 9.84 (s, 1H), 8.74 (s, 1H), 8.13 (d, $J$ = 9.0 Hz, 1H), 7.86–7.80 (m, 3H), 7.58 (d, $J$ = 8.4 Hz, 1H), 7.54–7.48 (m, 2H), 7.45 (dd, $J$ = 3.6, 1.2 Hz, 1H), 7.13 (dd, $J$ = 5.1, 3.6 Hz, 1H), 4.18 (t, $J$ = 8.7 Hz, 2H), 3.22 (t, $J$ = 8.7 Hz, 2H), 2.20 (s, 3H), 1.46 (s, 9H).

#### *4.1.33. Tert-Butyl (2-(1-butyrylindoline-5-carboxamido)-4-(thiophen-2-yl)phenyl)carbamate (35b)*

The title compound was obtained from compound **34b** in a manner similar to that described for the preparation of **31**: $^1$H NMR (300 MHz, DMSO-$d_6$) $\delta$ 9.83 (s, 1H), 8.74 (s, 1H), 8.16 (d, $J$ = 8.4 Hz, 1H), 7.85–7.82 (m, 3H), 7.60–7.44 (m,4H), 7.15–7.11 (m, 1H), 4.17 (t, $J$ = 8.4 Hz, 2H), 3.21 (t, $J$ = 8.4 Hz, 2H), 2.48–2.45 (m, 1H), 1.67–1.59 (m, 2H), 1.56 (s, 9H), 0.95 (t, $J$ = 7.5 Hz, 3H).

### 4.1.34. Tert-Butyl (2-(1-isobutyrylindoline-5-carboxamido)-4-(thiophen-2-yl)phenyl)carbamate (35c)

The title compound was obtained in 90% overall yield from compound **34c** in a manner similar to that described for the preparation of **31**: $^1$H NMR (300 MHz, DMSO-$d_6$) $\delta$ 9.83 (s, 1H), 8.75 (s, 1H), 8.19 (d, $J$ = 6.9 Hz, 1H), 7.85–7.81 (m, 3H), 7.59–7.44 (m, 4H), 7.15–7.11 (m, 1H), 4.25 (t, $J$ = 8.7 Hz, 2H), 3.23 (t, $J$ = 8.7 Hz, 2H), 2.89–2.85 (m, 1H), 1.46 (s, 9H), 1.12 (d, $J$ = 6.9 Hz, 6H).

### 4.1.35. Tert-Butyl (2-(1-(methylsulfonyl)indoline-5-carboxamido)-4-(thiophen-2-yl)phenyl)-carbamate (35d)

The title compound was obtained in 90% overall yield from compound **34d** in a manner similar to that described for the preparation of **31**: $^1$H NMR (300 MHz, DMSO-$d_6$) $\delta$ 9.85 (s, 1H), 8.70 (s, 1H), 7.89–7.86 (m, 2H), 7.80 (d, $J$ = 2.1 Hz, 1H), 7.60 (d, $J$ = 8.4 Hz, 1H), 7.54–7.48 (m, 2H), 7.44 (dd, $J$ = 3.6, 0.9 Hz, 1H), 7.36 (d, $J$ = 9.0 Hz, 1H), 7.15–7.11 (m, 1H), 4.03 (t, $J$ = 8.4 Hz, 2H), 3.20 (t, $J$ = 8.4 Hz, 2H), 3.08 (s, 3H), 1.46 (s, 9H).

### 4.1.36. Tert-Butyl (2-(1-(phenylsulfonyl)indoline-5-carboxamido)-4-(thiophen-3-yl)phenyl)-carbamate (35e)

The title compound was obtained in 90% overall yield from compound **34e** in a manner similar to that described for the preparation of **31**: $^1$H NMR (300 MHz, DMSO-$d_6$) $\delta$ 9.81 (s, 1H), 8.69 (s, 1H), 7.91–7.84 (m, 3H), 7.77–7.69 (m, 3H), 7.64–7.57 (m, 4H), 7.53–7.47 (m, 2H), 7.43 (dd, $J$ = 3.6, 1.2 Hz, 1H), 7.12 (dd, $J$ = 5.1, 3.6 Hz, 1H), 4.01 (t, $J$ = 8.4 Hz, 2H), 3.04 (t, $J$ = 8.4 Hz, 2H), 1.44 (s, 9H).

### 4.1.37. 1,2,3,4-Tetrahydroquinoline-6-carboxylic acid (37)

A mixture of quinoline-6-carboxylic acid (**36**, 1 g, 5.8 mmol), ammonium formate (1.82 g, 28.9 mmol) and 10% Pd/C (0.61 g, 0.58 mmol) in MeOH (30 ml) was refluxed for 2 h. After completion of the reaction, the mixture was filtered through celite, and the solvent was removed *in vacuo*. Water was added to the residue which was extracted several times with DCM and EtOAc. The organic layers were combined, dried over anhydrous $MgSO_4$, and evaporated under reduced pressure to yield the title compound (0.5 g, 49%). $^1$H NMR (300 MHz, DMSO-$d_6$) $\delta$ 11.83 (s, 1H), 7.46–7.41 (m, 2H), 6.49 (s, 1H), 6.42–6.38 (m, 1H), 3.25–3.19 (m, 2H), 2.66 (t, $J$ = 6.0 Hz, 2H), 7.82–7.73 (m, 2H).

### 4.1.38. 1-Acetyl-1,2,3,4-tetrahydroquinoline-6-carboxylic acid (38)

Acetyl chloride ($CH_3COCl$, 0.08 ml, 1.13 mmol) was added to a solution of compound **37** (0.2 g, 1.13 mmol) and TEA (0.16 ml, 1.13 mmol) in DCM (6 ml), and the mixture was stirred at rt for 20 min. After the reaction, 5~6 equivalents of 1 N $NaOH_{(aq)}$ were added, and the mixture water was added to the mixture which was then washed with EtOAc. The aqueous layer was then acidified with 1 N $HCl_{(aq)}$ and extracted with EtOAc. The organic layers were collected, dried over anhydrous $MgSO_4$ and concentrated to afford the title compound (0.15 g, 61%). $^1$H NMR (300 MHz, DMSO-$d_6$) $\delta$ 7.80–7.60 (m, 3H), 3.70 (t, $J$ = 6.3 Hz, 2H), 2.76 (t, $J$ = 6.6 Hz, 2H), 2.21 (s, 3 H), 1.92–1.83 (m, 2H).

### 4.1.39. Tert-Butyl (2-(1-acetyl-1,2,3,4-tetrahydroquinoline-6-carboxamido)-4-(thiophen-2- l)phenyl) carbamate (39)

The title compound was obtained in 80% overall yield from compound **38** in a manner similar to that described for the preparation of **31**: $^1$H NMR (300 MHz, DMSO-$d_6$) $\delta$ 9.88 (s, 1H), 8.71 (s, 1H), 7.83–7.76 (m, 3H), 7.74–7.68 (m, 1H), 7.60 (d, $J$ = 8.4 Hz, 1H), 7.54–7.48 (m, 2H), 7.45 (dd, $J$ = 3.6, 0.9 Hz, 1H), 7.13 (dd, $J$ = 5.1, 3.6 Hz, 1H), 3.73 (t, $J$ = 6.3 Hz, 2H), 2.78 (t, $J$ = 6.3 Hz, 2H), 2.23 (s, 3H), 1.91 (p, $J$ = 6.3 Hz, 2H), 1.46 (s, 9H).

### 4.1.40. Methyl 4-(pyridin-2-ylamino)benzoate (41a)

A mixture of methyl-4-aminobenzoate (**40a**, 2.1 g, 13.3 mmol) and 2-bromopyridine (1 g, 6.33 mmol) was heated to 140 °C and reacted for 2 h. After cooling down, water and 1 N $HCl_{(aq)}$ (13 ml) was added to the mixture, and the solution was washed with EtOAc. The organic layer was removed, and the aqueous layer was basified with 5 N $NaOH_{(aq)}$, followed by extraction with EtOAc. The organic layer was collected, dried over anhydrous $MgSO_4$, concentrated, and purified by flash column chromatography (DCM then DCM/MeOH = 20/1) to yield the title compound (0.87 g, 60%). $^1$H NMR (300 MHz, DMSO-$d_6$) $\delta$ 9.52 (s, 1H), 8.25–8.21 (m, 1H), 7.89–7.80 (m, 4H), 7.67–7.60 (m, 1H), 6.94–6.90 (m, 1H), 6.88–6.82 (m, 1H), 3.80 (s, 3H).

### 4.1.41. Methyl 4-(2-oxopyrrolidin-1-yl)benzoate (41b)

A mixture of methyl 4-bromobenzoate (**40b**, 2.4 g, 11.2 mmol), 2-pyrrolidone (0.8 g, 9.4 mmol), $Pd_2(dba)_3$ (0.22 g, 0.24 mmol), xantphos (0.4 g, 0.71 mmol), $Cs_2CO_3$ (4.3 g,13.16 mmol) and *p*-dioxane (30 ml) was stirred at 90 °C for 2 h. After completion of the reaction, the mixture was filtered through celite, and the solvent was removed *in vacuo*. The residue was purified by flash column chromatography (Hex/EtOAc = 3/2) to afford the title compound (2.02 g, 98%). $^1$H NMR (300 MHz, $CDCl_3$) $\delta$ 8.03 (d, $J$ = 9.0 Hz, 2H), 7.72 (d, $J$ = 9.0 Hz, 2H), 3.92–3.87 (m, 5H), 2.64 (t, $J$ = 8.1 Hz, 2H), 2.24–2.13 (m, 2H).

### 4.1.42. Methyl 4-(2-oxopiperidin-1-yl)benzoate (41c)

A mixture of 2-piperdone (0.5 g, 5 mmol), methyl 4-bromobenzoate (**40b**, 1.26 g, 5.85 mmol), $Pd_2(dba)_3$ (0.12 g, 0.13 mmol), xantphos (0.22 g, 0.38 mmol), $Cs_2CO_3$ (2.3 g, 7 mmol) and *p*-dioxane (16 ml) was stirred at reflux for 3 h. After completion of the reaction, the mixture was filtered through celite, and the solvent was removed *in vacuo*. The residue was purified by flash column chromatography (Hex/EtOAc = 1/1) to afford the title compound (1.11 g, 99%). $^1$H NMR (300 MHz, DMSO-$d_6$) $\delta$ 8.05 (d, $J$ = 8.7 Hz, 2H), 7.36 (d, $J$ = 9.0 Hz, 2H), 3.91 (s, 3H), 3.71–3.66 (m, 2H), 2.61–2.56 (m, 2H), 1.98–1.92 (m, 4H).

### 4.1.43. Methyl 4-(methylcarbamoyl)benzoate (41d)

A mixture of 4-(methoxycarbonyl)benzoic acid (**40c**, 0.5 g, 2.8 mmol), methylamine hydrochloride (0.2 g, 3 mmol), EDC·HCl (1-ethyl-3-(3-dimethylaminopropyl)-carbodiimide hydrochloride, 1 g, 5.6 mmol) and DMAP (4-dimethylaminopyridine, 1.5 g, 12.6 mmol) in DMF (5 ml) was stirred at rt for 5 h. The reaction was quenched with water, added 3 N $HCl_{(aq)}$, and extracted with EtOAc. The organic layer was washed with sat. $NaHCO_{3(aq)}$, dried over anhydrous $MgSO_4$ and concentrated to afford the title compound (0.28 g, 52%). $^1$H NMR (300 MHz, $CDCl_3$) $\delta$ 8.08 (d, $J$ = 8.4 Hz, 2H), 7.81 (d, $J$ = 8.4 Hz, 2H), 6.27 (s, 1H), 3.93 (s, 3H), 3.03 (d, $J$ = 4.8 Hz, 3H).

#### 4.1.44. Methyl 4-(oxazol-2-yl)benzoate (41e)

A mixture of 4-(methoxycarbonyl)benzoic acid (**40c**, 6 g, 33.3 mmol), aminoacetaldehyde dimethyl acetal (3.52 g, 33.3 mmol), EDC·HCl (12.8 g, 66.6 mmol), and NMM (N-methylmorpholine, 9 ml, 81.8 mmol) in DCM (67 ml) was stirred at rt for 22 h. The reaction was quenched with water, acidified with 3 N $HCl_{(aq)}$ to pH ≈ 2, and extracted with DCM. The organic layer was dried over anhydrous $MgSO_4$, and the solvent was removed *in vacuo* to afford the crude product. Phosphorus pentoxide ($P_4O_{10}$, 15 g, 52.8 mmol) was added to a solution of the crude product (3 g, 11.2 mmol) in $MeSO_3H$ (22 ml) in an ice bath, and the mixture was heated to 140 °C for 9 h under an inert atmosphere. After cooling down, water was added to the mixture, which was then extracted with DCM, and dried over anhydrous $MgSO_4$. After removal of the solvent, the crude material was purified by flash column chromatography (Hex/EtOAc = 3/2) to afford the title compound (0.23 g, 7.8%). $^1$H NMR (300 MHz, $CDCl_3$) δ 8.13 (s, 4H), 7.76 (d, J = 0.9 Hz, 1H), 7.29 (d, J = 0.6 Hz, 1H), 3.95 (s, 3H).

#### 4.1.45. Methyl 4-(oxazol-5-yl)benzoate (41f)

A mixture of methyl 4-formylbenzoate (**40d**, 0.6 g, 3.66 mmol), TosMIC (0.86 g, 4.39 mmol), and $K_2CO_3$ (0.66 g, 4.75 mmol) in MeOH (10 ml) was stirred at reflux for 3 h. After cooling down, the resulting suspension was filtered to afford the title compound (0.48 g, 65%). $^1$H NMR (300 MHz, $CDCl_3$) δ 8.10 (d, J = 9.0 Hz, 2H), 7.97 (s, 1H), 7.73 (d, J = 8.7 Hz, 2H), 7.48 (s, 1H), 3.94 (s, 3H).

#### 4.1.46. Methyl 4-(1H-benzo[d]imidazol-2-yl)benzoate (41g)

A mixture of methyl 4-formylbenzoate (**40d**, 1.5 g, 9.14 mmol), *o*-phenylenediamine (1 g, 9.25 mmol) and $Na_2S_2O_4$ (1.6 g, 9.2 mmol) in DMF (6 ml) was stirred at 70 °C for 18 h. After completion of the reaction, ice-cold water was added, and the resulting suspension was filtered to afford the title compound (2.21 g, 95%). $^1$H NMR (300 MHz, DMSO-$d_6$) δ 8.31 (d, J = 8.7 Hz, 2H), 8.12 (d, J = 8.7 Hz, 2H), 7.65–7.61 (m, 2H), 7.28–7.21 (m, 2H), 3.89 (s, 3H).

#### 4.1.47. 4-(Pyridin-2-ylamino)benzoic acid (42a)

A mixture of compound **41a** (0.55 g, 2.40 mmol) and 1 N $LiOH_{(aq)}$ (7.2 ml) was stirred in *p*-dioxane (10 ml) at rt for 3 h. The solution was quenched with water and neutralised with 3 N $HCl_{(aq)}$. The solvent was removed under reduced pressure, and the crude product (0.93 g) was used in next step without purification.

#### 4.1.48. 4-(2-Oxopyrrolidin-1-yl)benzoic acid (42b)

The title compound was obtained in 76% overall yield from compound **41b** in a manner similar to that described for the preparation of **30**: $^1$H NMR (300 MHz, MeOH-$d_4$ + $CDCl_3$) δ 8.02 (d, J = 9.0 Hz, 2H), 7.75 (d, J = 9.0 Hz, 2H), 3.96 (t, J = 7.2 Hz, 2H), 2.63 (t, J = 7.8 Hz, 2H), 2.25–2.14 (m, 2H).

#### 4.1.49. 4-(2-Oxopiperidin-1-yl)benzoic acid (42c)

The title compound was obtained in 68% overall yield from compound **41c** in a manner similar to that described for the preparation of **30**: $^1$H NMR (300 MHz, DMSO-$d_6$) δ 7.93 (d, J = 8.4 Hz, 2H), 7.42 (d, J = 8.4 Hz, 2H), 3.65 (t, J = 5.7 Hz, 2H), 2.42 (t, J = 6.0 Hz, 2H), 1.91–1.82 (m, 4H).

#### 4.1.50. 4-(Methylcarbamoyl)benzoic acid (42d)

The title compound was obtained in 99% overall yield from compound **41d** in a manner similar to that described for the preparation of **34a**: $^1$H NMR (300 MHz, DMSO-$d_6$) δ 8.59 (d, J = 4.5 Hz, 1H), 8.00 (d, J = 8.4 Hz, 2H), 7.91 (d, J = 8.4 Hz, 2H), 2.79 (d, J = 4.5 Hz, 3H).

#### 4.1.51. 4-(Oxazol-2-yl)benzoic acid (42e)

The title compound was obtained in 89% overall yield from compound **41e** in a manner similar to that described for the preparation of **30**: $^1$H NMR (300 MHz, DMSO-$d_6$) δ 8.29 (d, J = 0.6 Hz, 1H), 8.12–8.05 (m, 4H), 7.45 (d, J = 0.6 Hz, 1H).

#### 4.1.52. 4-(Oxazol-5-yl)benzoic acid (42f)

The title compound was obtained in 100% overall yield from compound **41f** in a manner similar to that described for the preparation of **30**: $^1$H NMR (300 MHz, DMSO-$d_6$) δ 8.53 (s, 1H), 8.03 (d, J = 8.4 Hz, 2H), 7.87–7.83 (m, 3H).

#### 4.1.53. 4-(1h-Benzo[d]imidazol-2-yl)benzoic acid (42g)

The title compound was obtained in 77% overall yield from compound **41g** in a manner similar to that described for the preparation of **34a**: $^1$H NMR (300 MHz, DMSO-$d_6$) δ 8.29 (d, J = 8.4 Hz, 2H), 8.11 (d, J = 8.4 Hz, 2H), 7.66–7.61 (m, 2H), 7.29–7.23 (m, 2H).

#### 4.1.54. Tert-Butyl (2-(4-(pyridin-2-ylamino)benzamido)-4-(thiophen-2-yl)phenyl) carbamate (43a)

The title compound was obtained from compound **42a** in a manner similar to that described for the preparation of **31**: $^1$H NMR (600 MHz, DMSO-$d_6$) δ 9.76 (s, 1H), 9.45 (s, 1H), 8.71 (s, 1H), 8.24 (dd, J = 5.4, 1.8 Hz, 1H), 7.92 (d, J = 7.2 Hz, 2H), 7.85 (d, J = 9.0 Hz, 2H), 7.84 (d, J = 2.4 Hz, 1H), 7.66–7.62 (m, 1H), 7.59 (d, J = 8.4 Hz, 1H), 7.53 (dd, J = 4.8, 1.2 Hz, 1H), 7.50 (dd, J = 8.4, 1.4 Hz, 1H), 7.45 (dd, J = 4.2, 1.2 Hz, 1H), 7.13 (dd, J = 5.4, 3.6 Hz, 1H), 6.92 (d, J = 8.4 Hz, 1H), 6.86–6.83 (m, 1H), 1.47 (s, 9H).

#### 4.1.55. Tert-Butyl (2-(4-(2-oxopyrrolidin-1-yl)benzamido)-4-(thiophen-2-yl)phenyl) carbamate (43b)

The title compound was obtained in 85% overall yield from compound **42b** in a manner similar to that described for the preparation of **31**: $^1$H NMR (300 MHz, DMSO-$d_6$) δ 9.89 (s, 1H), 8.72 (s, 1H), 8.00 (d, J = 8.7 Hz, 2H), 7.84 (d, J = 8.7 Hz, 2H), 7.82 (d, J = 2.1 Hz, 1H), 7.61 (d, J = 8.4 Hz, 1H), 7.54–7.49 (m, 2H), 7.46 (dd, J = 3.6, 1.2 Hz, 1H), 7.13 (dd, J = 5.1, 3.6 Hz, 1H), 3.90 (t, J = 7.2 Hz, 2H), 2.55 (t, J = 7.8 Hz, 2H), 2.15–2.04 (m, 2H), 1.45 (s, 9H).

#### 4.1.56. Tert-Butyl (2-(4-(2-oxopiperidin-1-yl)benzamido)-4-(thiophen-2-yl)phenyl) carbamate (43c)

The title compound was obtained in 82% overall yield from compound **42c** in a manner similar to that described for the preparation of **31**: $^1$H NMR (300 MHz, DMSO-$d_6$) δ 9.91 (s, 1H), 8.71 (s, 1H), 7.98 (d, J = 8.7 Hz, 2H), 7.82 (d, J = 2.1 Hz, 1H), 7.63 (d, J = 8.7 Hz, 1H), 7.53–7.50 (m, 2H), 7.48 (d, J = 8.7 Hz, 2H), 7.45 (dd, J = 3.6, 1.2 Hz, 1H), 7.13 (dd, J = 5.1, 3.6 Hz, 1H), 3.68 (t, J = 6.0 Hz, 2H), 2.43 (t, J = 6.3 Hz, 2H), 1.93–1.80 (m, 4H), 1.46 (s, 9H).

#### 4.1.57. Tert-Butyl (2-(4-(methylcarbamoyl)benzamido)-4-(thiophen-2-yl)phenyl) carbamate (43d)

The title compound was obtained in 70% overall yield from compound **42d** in a manner similar to that described for the preparation of **31**: $^1$H NMR (300 MHz, DMSO-$d_6$) $\delta$ 9.99 (s, 1H), 8.77 (s, 1H), 8.61 (d, $J$ = 4.5 Hz, 1H), 8.05 (d, $J$ = 8.4 Hz, 2H), 7.98 (d, $J$ = 8.7 Hz, 2H), 7.82 (d, $J$ = 2.1 Hz, 1H), 7.64 (d, $J$ = 8.7 Hz, 1H), 7.54–7.50 (m, 2H), 7.46 (dd, $J$ = 3.6, 1.2 Hz, 1H), 7.13 (dd, 5.1, 3.6 Hz, 1H), 2.82 (d, $J$ = 4.5 Hz, 3H), 1.46 (s, 9H).

#### 4.1.58. Tert-Butyl (2-(4-(oxazol-2-yl)benzamido)-4-(thiophen-2-yl)phenyl)carbamate (43e)

The title compound was obtained in 74% overall yield from compound **42e** in a manner similar to that described for the preparation of **31**: $^1$H NMR (300 MHz, DMSO-$d_6$) $\delta$ 9.84 (s, 1H), 8.35 (s, 1H), 8.20 (s, 4H), 8.11 (d, $J$ = 0.6 Hz, 1H), 8.07 (d, $J$ = 2.1 Hz, 1H), 7.67 (d, $J$ = 8.4 Hz, 1H), 7.55 (dd, $J$ = 8.4, 2.1 Hz, 1H), 7.46–7.44 (m, 1H), 7.44 (s, 1H), 7.38 (d, $J$ = 0.6 Hz, 1H), 7.15–7.11 (m, 1H), 1.51 (s, 9H).

#### 4.1.59. Tert-Butyl (2-(4-(oxazol-5-yl)benzamido)-4-(thiophen-2-yl)phenyl)carbamate (43f)

The title compound was obtained in 80% overall yield from compound **42f** in a manner similar to that described for the preparation of **31**: $^1$H NMR (300 MHz, $CDCl_3$) $\delta$ 9.37 (s, 1H), 8.08–8.02 (m, 3H), 7.98 (s, 1H), 7.73 (d, $J$ = 8.7 Hz, 2H), 7.47 (s, 1H), 7.38 (dd, $J$ = 8.4, 2.4 Hz, 1H), 7.28–7.26 (m, 1H), 7.24 (dd, $J$ = 5.1, 1.2 Hz, 1H), 7.23 (d, $J$ = 2.4 Hz, 1H), 7.03 (dd, $J$ = 5.1, 3.6 Hz, 1H), 6.87 (s, 1H), 1.53 (s, 9H).

#### 4.1.60. Tert-Butyl (2-(4-(1H-benzo[d]imidazol-2-yl)benzamido)-4-(thiophen-2-yl)phenyl)-carbamate (43g)

The title compound was obtained in 80% overall yield from compound **42g** in a manner similar to that described for the preparation of **31**: $^1$H NMR (300 MHz, DMSO-$d_6$) $\delta$ 13.07 (s, 1H), 10.02 (s, 1H), 8.79 (s, 1H), 8.35 (d, $J$ = 8.4 Hz, 2H), 8.16 (d, $J$ = 8.4 Hz, 2H), 7.85 (d, $J$ = 2.1 Hz, 1H), 7.77–7.57 (m, 3H), 7.56–7.51 (m, 2H), 7.47 (dd, $J$ = 3.6, 1.2 Hz, 1H), 7.30–7.20 (m, 2H), 7.14 (dd, $J$ = 5.1, 3.6 Hz, 1H), 1.47 (s, 9H).

### 4.2. Biology

#### 4.2.1. In vitro cell viability assay

Human colorectal tumour cell lines (HCT116 and DLD1), human non-cancerous cell lines, kidney cells (293 T) and lung cells (IMR90), were obtained from the America Type Culture Collection (ATCC). All cell lines were cultivated in DMEM or RPMI 1640 medium (supplemented with 10% foetal bovine serum and 1% antibiotic-antimycotic) and maintained at 37 °C in a 5% $CO_2$ humidified incubator. 4,500 cells/well were seeded into 96-well plates. After an incubation time of 24 h, cells were treated with varying concentrations of synthetic compounds in quadruple wells. For indicated 48 h or 72 h treatments, the cell viability was performed by Cell Viability (MTT) Assay, with 3-(4,5-dimethylthiazol-2-yl)-2,5-diphenyltetrazolium bromide. The cells were incubated for 4–6 h, and the insoluble crystals were dissolved after adding dimethyl sulfoxide (DMSO). The absorbance value was detected by a spectrophotometer (BioTek Instruments) at 570 nm. The concentration required to suppress cell growth by 50% ($IC_{50}$) was estimated from nonlinear regression.

#### 4.2.2. In vitro cell cycle assay

HCT116 cells ($2.5 \times 10^5$) were seeded into 6-well plates and exposed with increasing doses (0.2 μM, 1.0 μM) of **8** and **16**. After 48 h of treatment, cells were harvested and fixed in pre-chilled 70% (v/v) EtOH at −20 °C. Then, the samples were washed with PBS twice, and stained by propidium iodide (50 μg/mL) containing RNase A (100 μg/mL). DNA content was detected by flow cytometric analysis with an Attune$^{TM}$ NxT Acoustic Focussing Cytometer (Thermo Fisher Scientific). Sub G1 peaks were analysed as indicative of apoptotic population.

#### 4.2.3. Western blot analysis

HCT116 cells were treated with as described previously before harvesting and lysis. Protein concentrations were quantified using a Bio-Rad protein assay. For Western blotting, protein extracts were separated by SDS polyacrylamide gel electrophoresis and transferred to PVDF membranes. The membranes were blocked with 5% non-fat dried milk in 1 × TBS containing 0.1% Tween 20 for 1 h at rt then probed with the indicated primary antibodies. The employed antibodies were obtained from the following sources: Acetyl-Histone H3 (Lys9), acetyl-α-tubulin (Lys40) were obtained from Cell Signalling Technology. Histone H3, α-tubulin, PARP (poly-ADP-ribose polymerase), PUMA (the p53 upregulated modulator of apoptosis), GAPDH were purchased from GeneTex. $p21^{Cip1/WAF1}$ was purchased from Santa Cruz. Signal intensity of protein expression was quantified using the ImageJ software.

## Disclosure statement

The authors report no declarations of interest.

## Funding

This research was supported by the Ministry of Science and Technology, Taiwan [grant no. MOST108-2320-B-038-042-MY3 and MOST 109-2320-B-038-038], Taiwan, and WanFang Hospital, Chi-Mei Medical Center, and Hualien Tzu-Chi Hospital Joint Cancer Center Grant-Focus on Colon Cancer Research [MOHW109-TDU-B-212-134020, supported by Health and welfare surcharge of tobacco products], Taiwan.

## References

1. Sharma S, Kelly TK, Jones PA. Epigenetics in cancer. Carcinogenesis 2010;31:27–36.
2. Biswas S, Rao CM. Epigenetic tools (The Writers, The Readers and The Erasers) and their implications in cancer therapy. Eur J Pharmacol 2018;837:8–24.
3. Falkenberg KJ, Johnstone RW. Histone deacetylases and their inhibitors in cancer, neurological diseases and immune disorders. Nat Rev Drug Discov 2014;13:673–91.
4. Marks PA, Xu WS. Histone deacetylase inhibitors: potential in cancer therapy. J Cell Biochem 2009;107:600–8.
5. Yang F, Zhao N, Ge D, Chen Y. Next-generation of selective histone deacetylase inhibitors. RSC Adv 2019;9:19571–83.
6. Li Y, Seto E. HDACs and HDAC inhibitors in cancer development and therapy. Cold Spring Harb Perspect Med 2016;6:a026831.

7. Xie HJ, Noh JH, Kim JK, et al. HDAC1 inactivation induces mitotic defect and caspase-independent autophagic cell death in liver cancer. PLoS One 2012;7:e34265.
8. Fritsche P, Seidler B, Schüler S, et al. HDAC2 mediates therapeutic resistance of pancreatic cancer cells via the BH3-only protein NOXA. Gut 2009;58:1399–409.
9. Bieliauskas AV, Pflum MK. Isoform-selective histone deacetylase inhibitors. Chem Soc Rev 2008;37:1402–13.
10. Wong JC, Hong R, Schreiber SL. Structural biasing elements for in-cell histone deacetylase paralog selectivity. J Am Chem Soc 2003;125:5586–7.
11. Beckers T, Burkhardt C, Wieland H, et al. Distinct pharmacological properties of second generation HDAC inhibitors with the benzamide or hydroxamate head group. Int J Cancer 2007;121:1138–48.
12. Moradei O, Raeppel S, Leit S, et al. Discovery of N-(2-aminophenyl)-4-[(4-pyridin-3-ylpyrimidin-2-ylamino)methyl]benzamide (MGCD0103), an orally active histone deacetylase inhibitor. J Med Chem 2008;51:4072–5.
13. Wang DF, Wiest O, Helquist P, et al. On the function of the 14 A long internal cavity of histone deacetylase-like protein: implications for the design of histone deacetylase inhibitors. J Med Chem 2004;47:3409–17.
14. Methot JL, Chakravarty PK, Chenard M, et al. Exploration of the internal cavity of histone deacetylase (HDAC) with selective HDAC1/HDAC2 inhibitors (SHI-1:2). Bioorg Med Chem Lett 2008;18:973–8.
15. Witter DJ, Harrington P, Wilson KJ, et al. Optimization of biaryl selective HDAC1&2 inhibitors (SHI-1:2). Bioorg Med Chem Lett 2008;18:726–31.
16. Moradei OM, Mallais TC, Frechette S, et al. Novel aminophenyl benzamide-type histone deacetylase inhibitors with enhanced potency and selectivity. J Med Chem 2007;50: 5543–6.
17. Lee HY, Pan SL, Su MC, et al. Furanylazaindoles: potent anticancer agents *in vitro* and *in vivo*. J Med Chem 2013;56: 8008–18.
