# Supplementary material for: Ring size changes in the development of class I HDAC inhibitors
Source: J Enzyme Inhib Med Chem. 2021 Jun 24;36(1):1387–401. doi: 10.1080/14756366.2021.1941920 (PMC8231390; doi:10.1080/14756366.2021.1941920)

Support Information

Ring Size Changes in the Development of Class I HDAC Inhibitors

Er-Chieh Cho, Chi-Yuan Liu, Di-Wei Tang, Hsueh-Yun Lee*

Contents:

Supplementary Table 1 ……………………………………………………………SI-2

Supplementary Figure 1……………………………………………………………SI-3

^{1}H NMR Spectra for compounds **7-20**…………………………………………SI-4

^{13}C NMR Spectra for compounds **7-20**………………………………………SI-18

Supplementary Table 1. Inhibitory activity (IC_{50}, μM[a]) of compounds in 293T and IMR90 cells after the treatment of tested compounds for 48 h.

Compound	IC_{50} (μM)	
	293T	IMR90
Compound 4	24.23 ± 5.48	51.39 ± 6.29
Compound 8	56.57 ± 7.99	>100
Compound 9	33.08 ± 15.55	>100
Compound 10	50.87 ± 39.1	>100
Compound 11	32.83 ± 8.29	>100
Compound 12	15.68 ± 4.58	39.56 ± 14.25
Compound 13	51.10 ± 10.43	20.17 ± 3.31
Compound 14	>300	20.05 ± 1.83
Compound 15	>300	74.02 ± 33.38
Compound 16	24.30 ± 4.14	19.34 ± 8.81

[a]The IC_{50} was estimated using GraphPad Prism 7 software and shown as mean ± SD from at last three independent experiments. [b]ND: not determined.

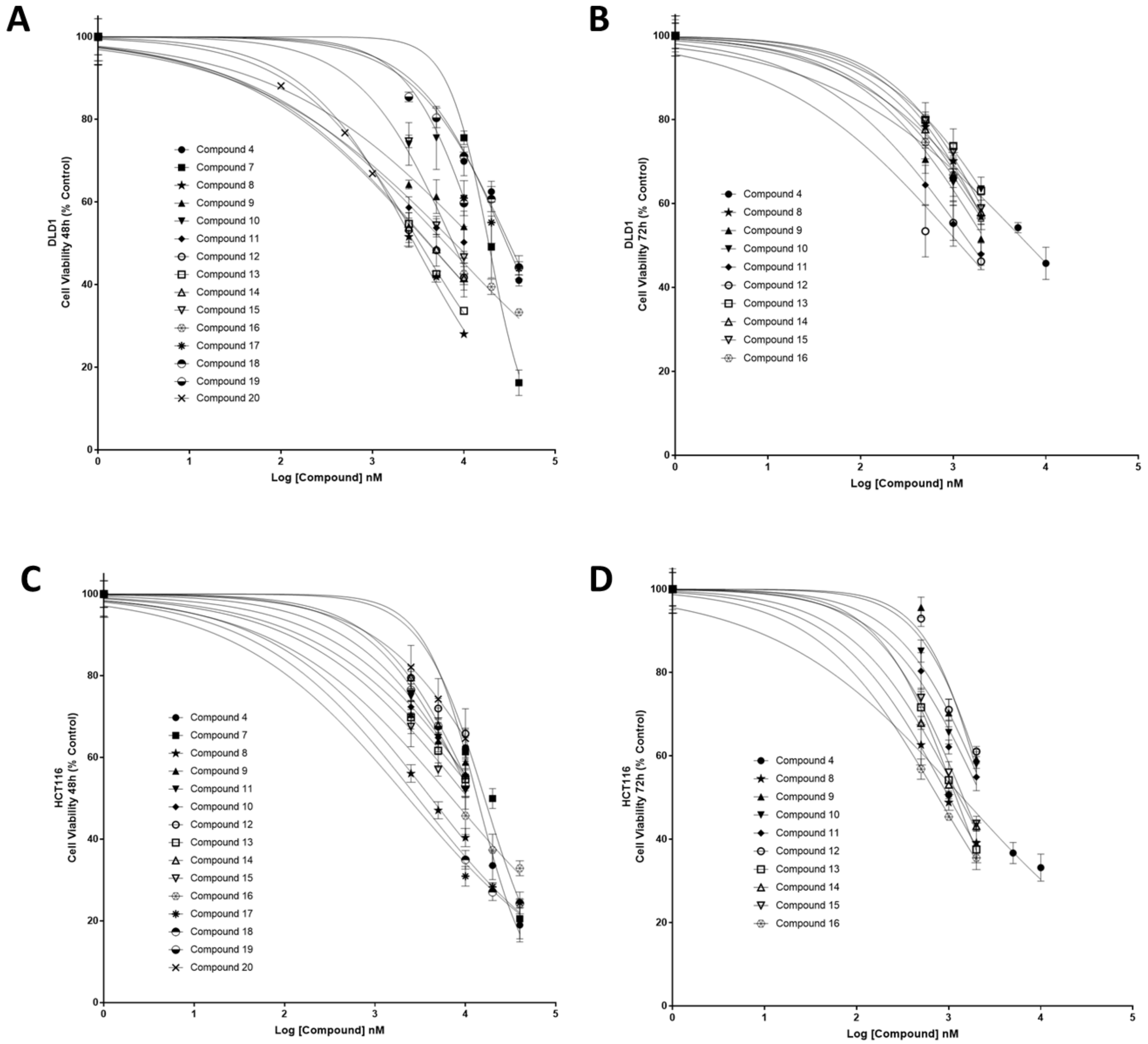

Supplementary Figure 1. IC_{50} curves of compounds in DLD1 and HCT116 cells at 48 h and 72 h.

^{1}H Spectra for compound 7

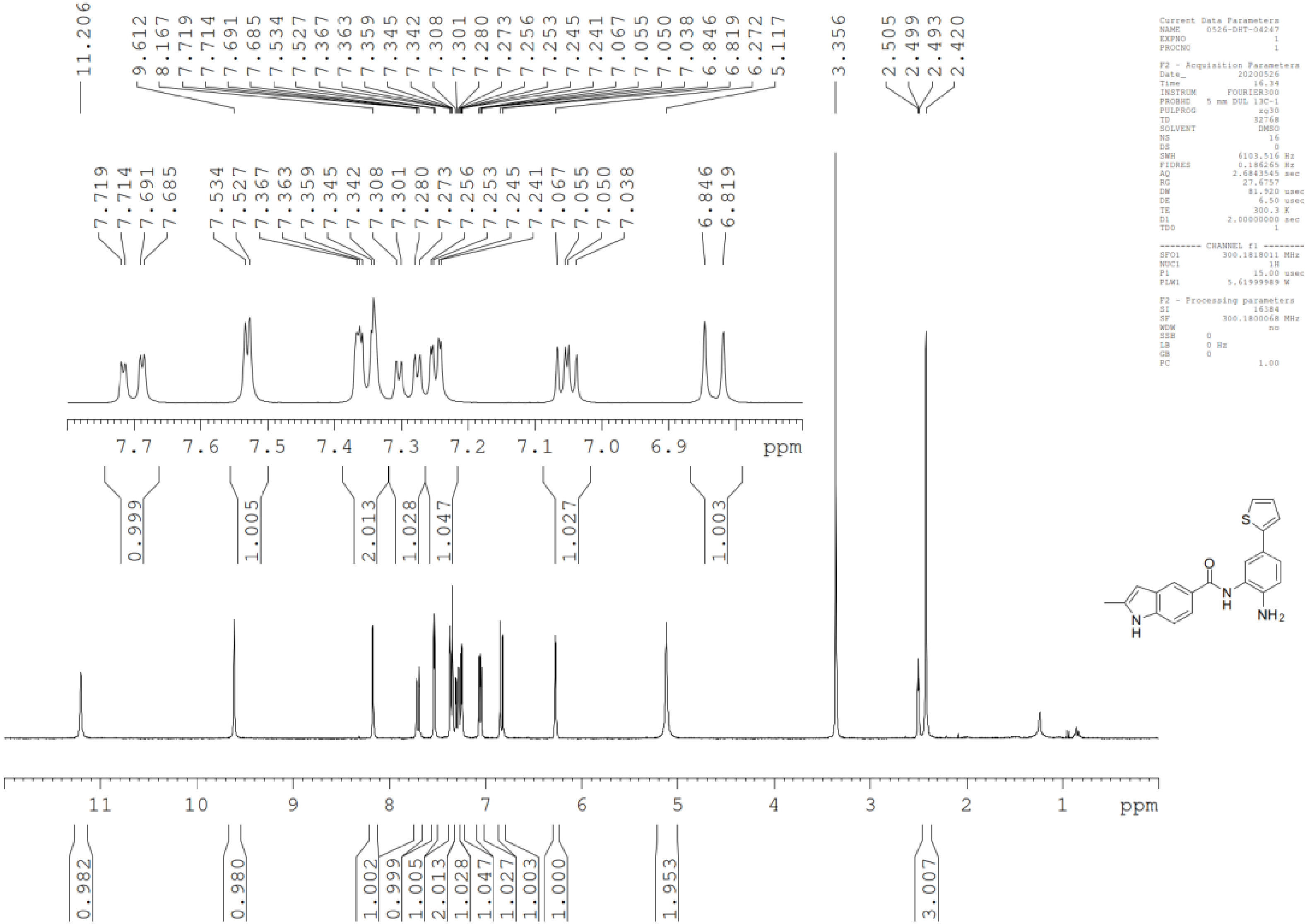

^{1}H Spectra for compound 8

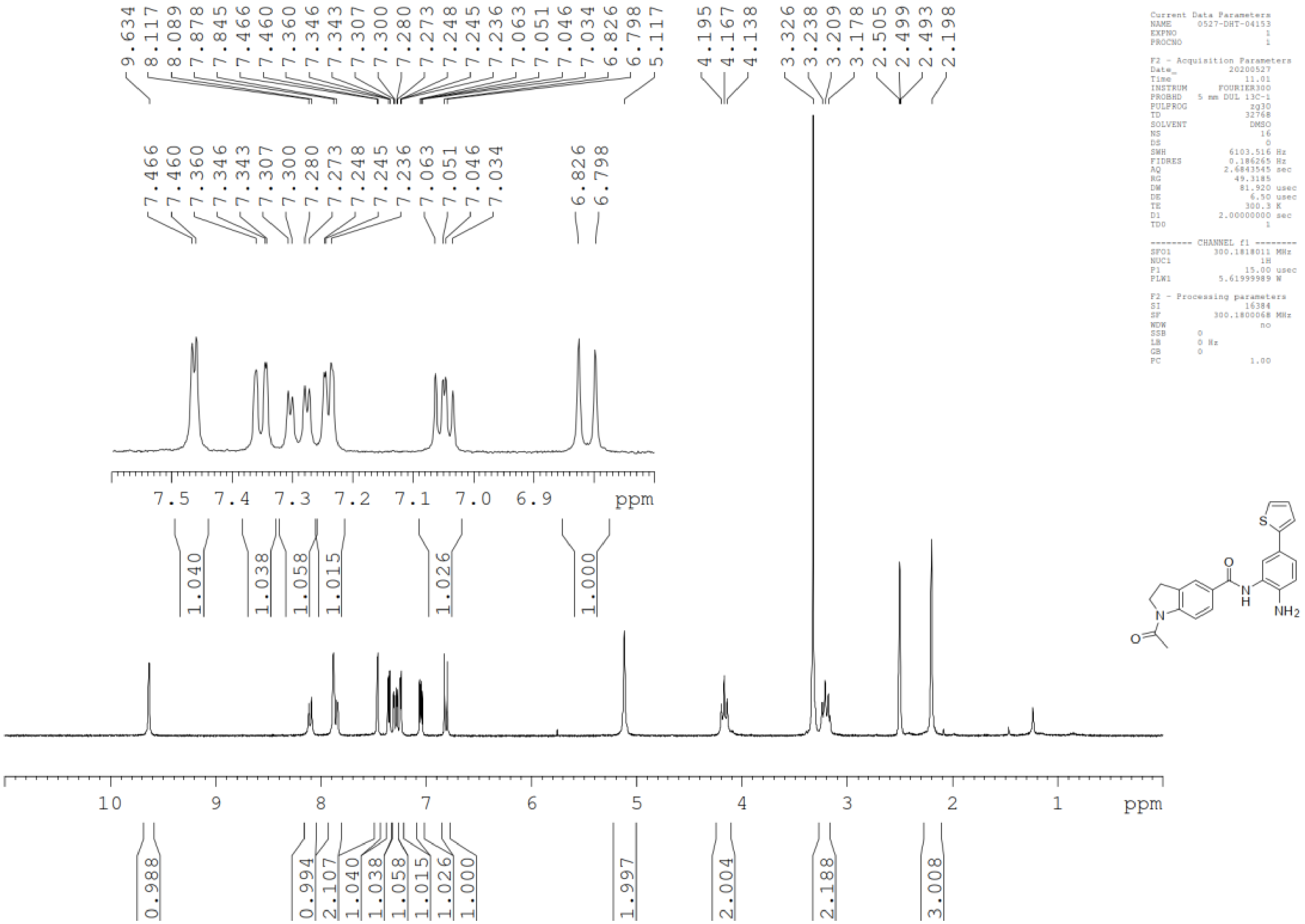

[1]H Spectra for compound 9

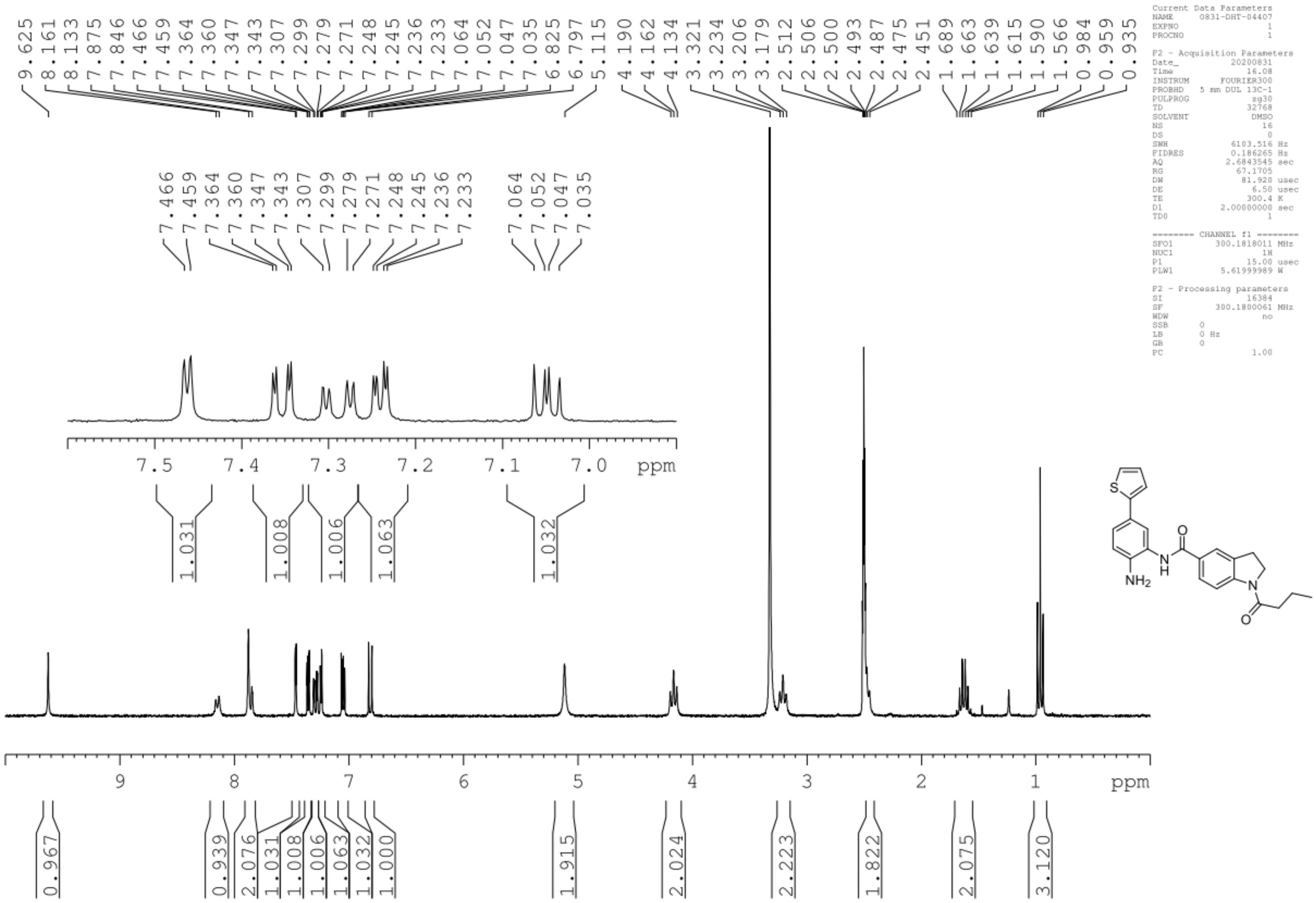

[1]H Spectra for compound 10

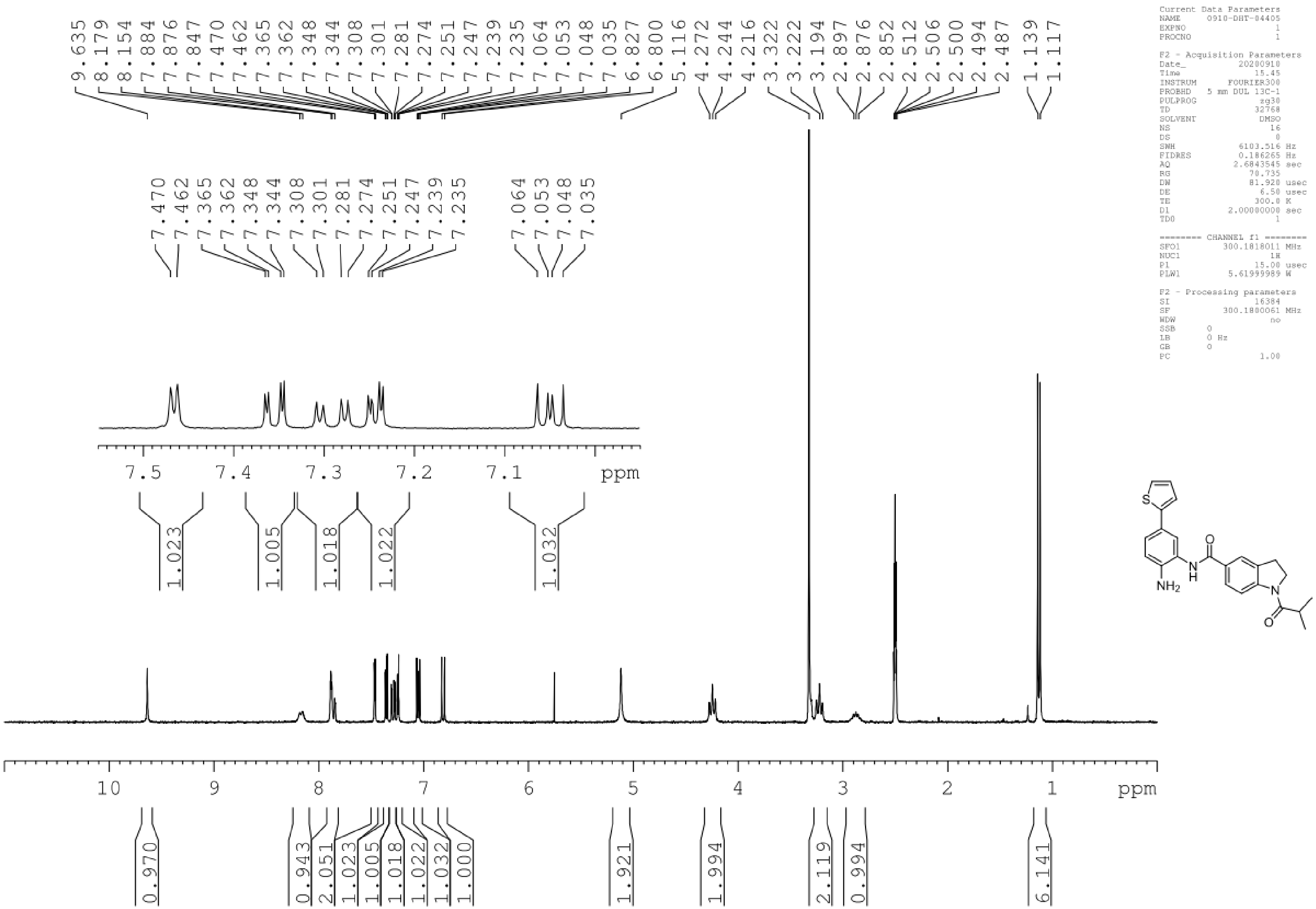

[1]H Spectra for compound 11

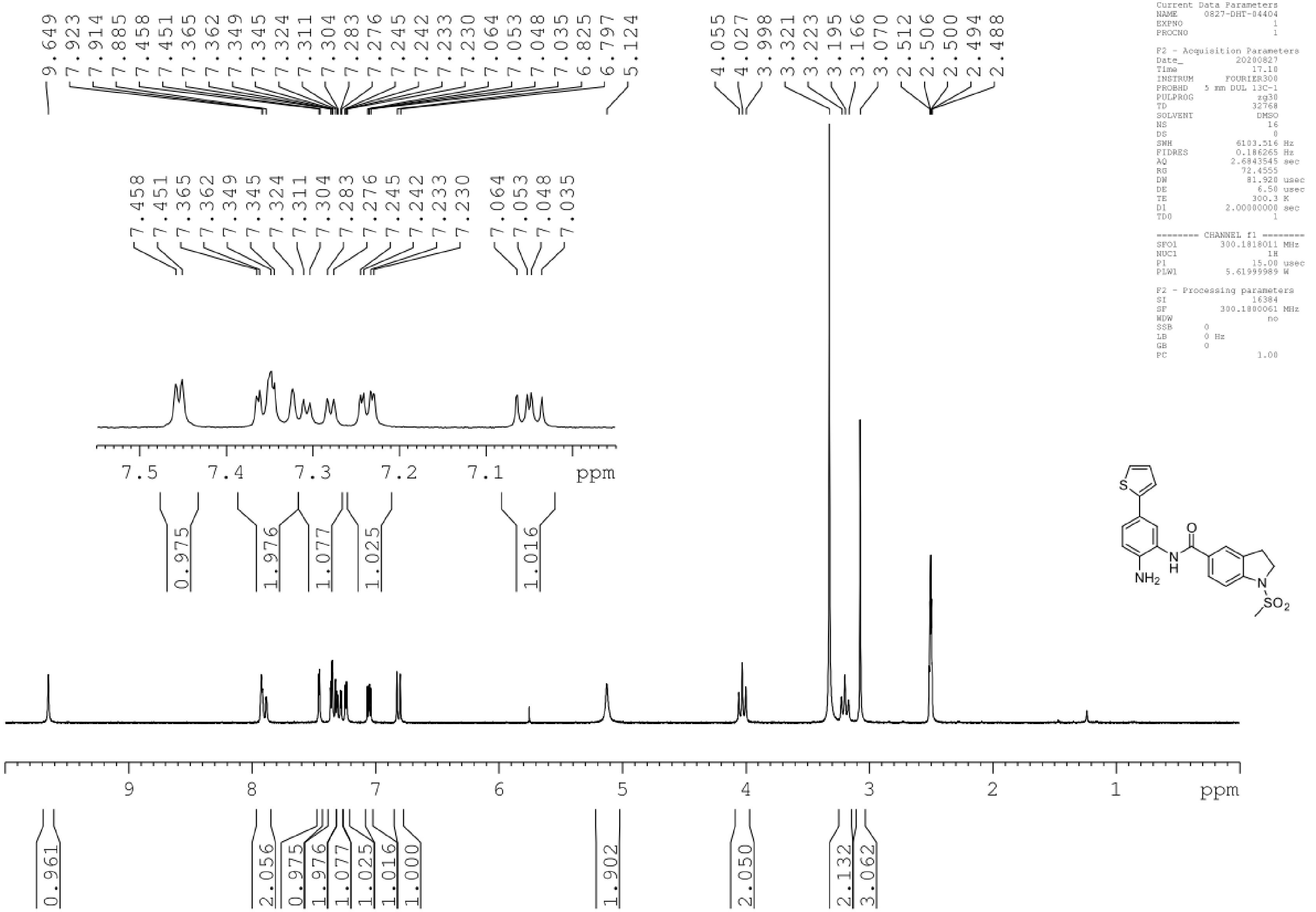

^{1}H Spectra for compound 12

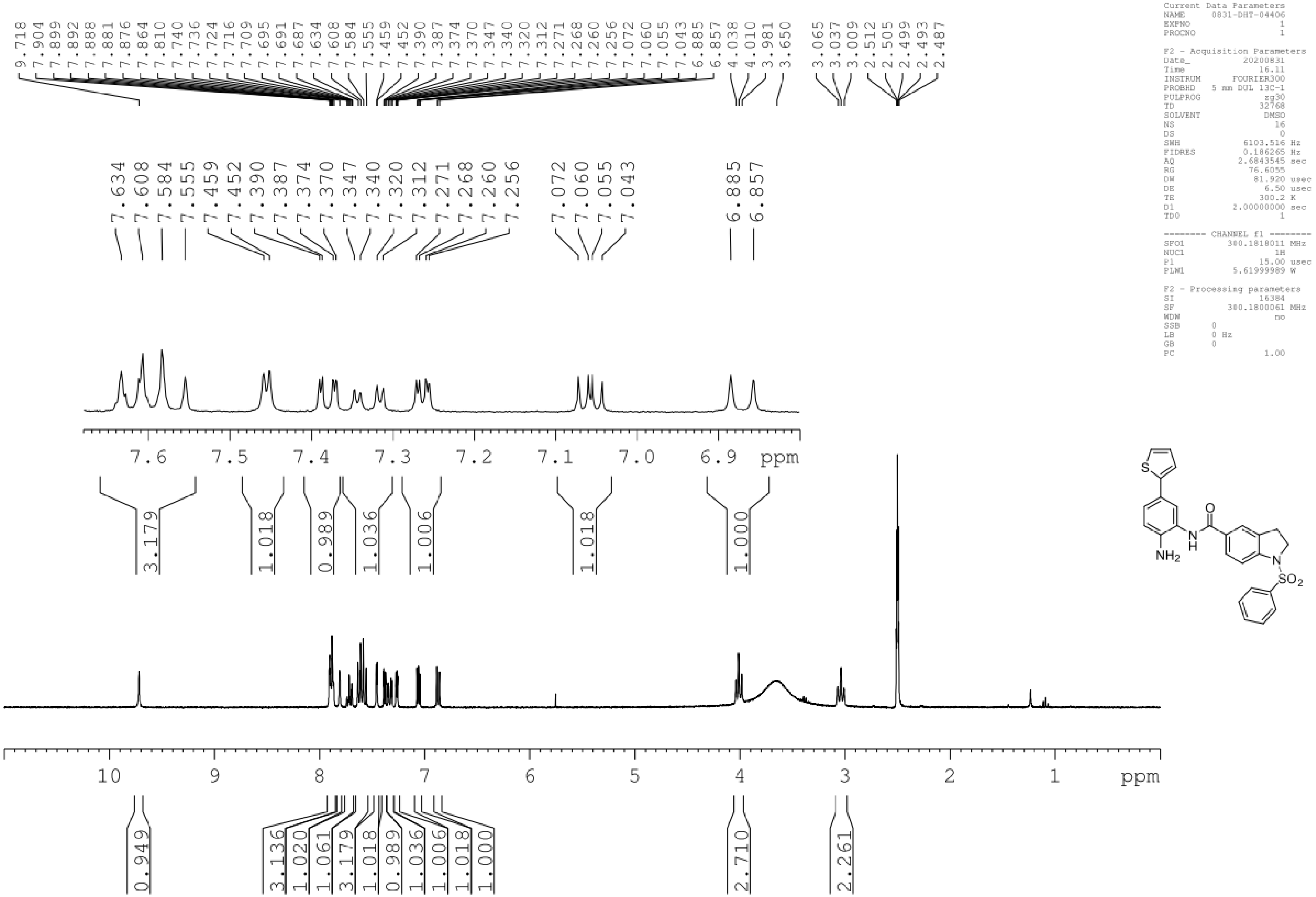

[1]H Spectra for compound 13

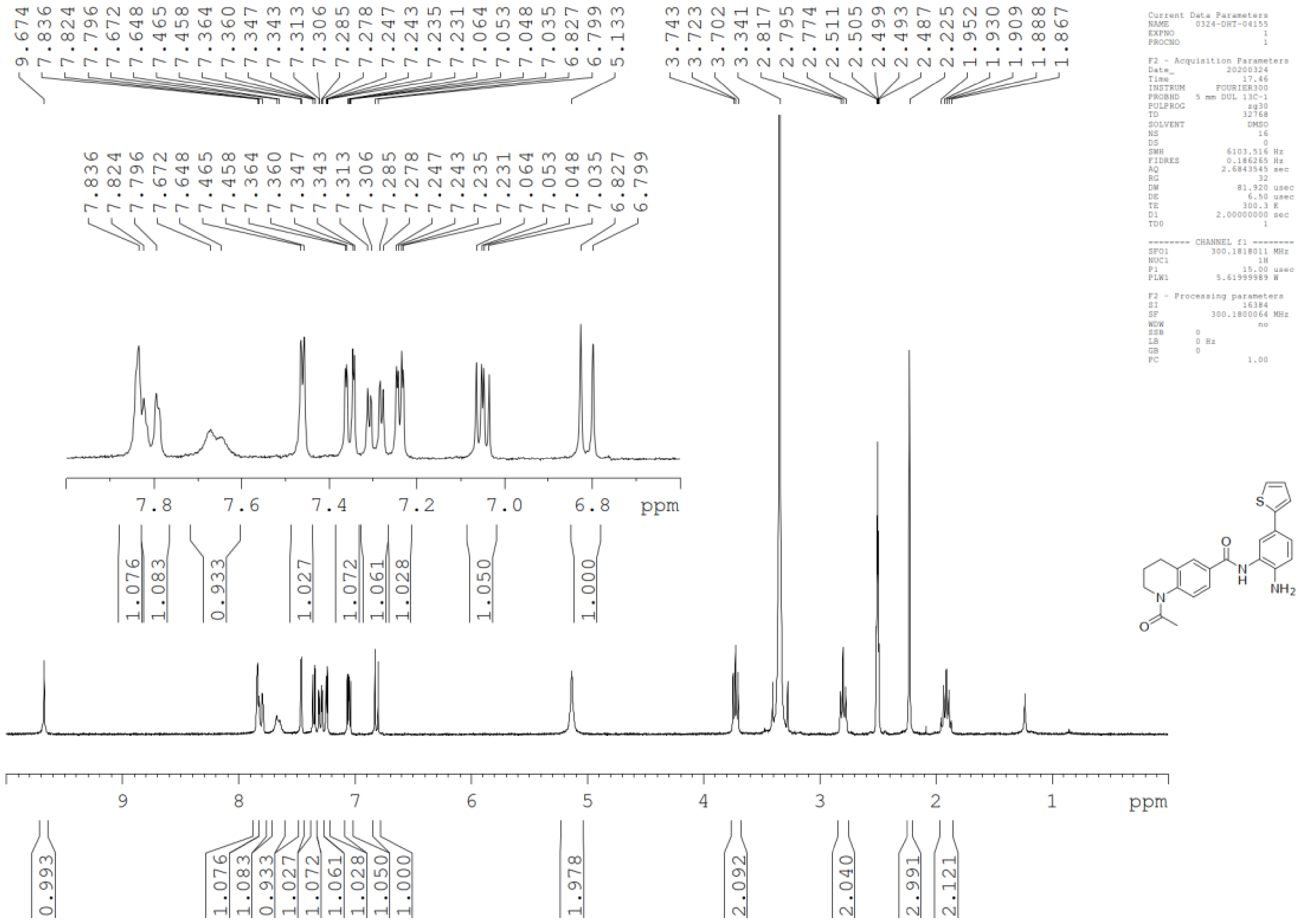

[1]H Spectra for compound 14

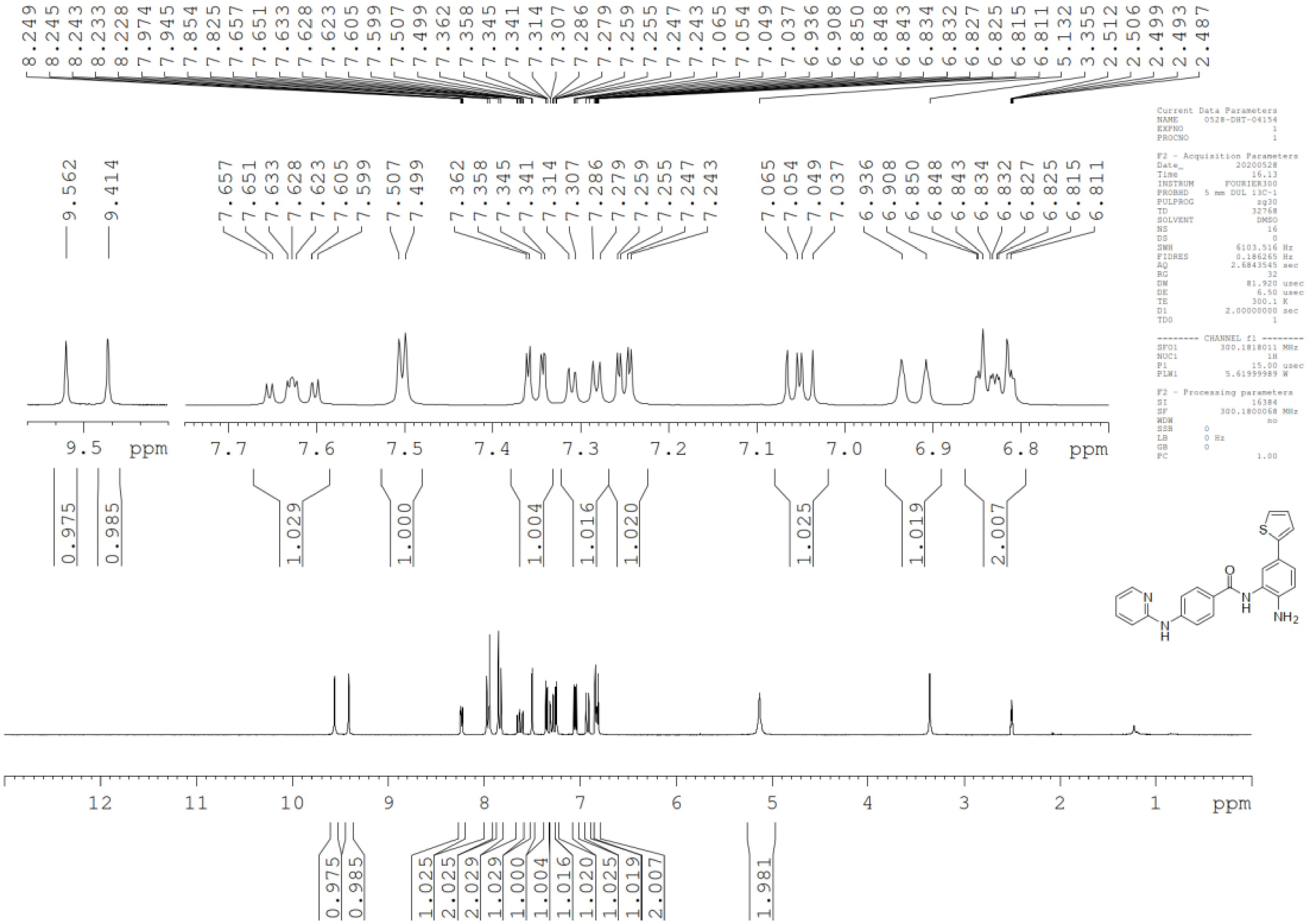

[1]H Spectra for compound 15

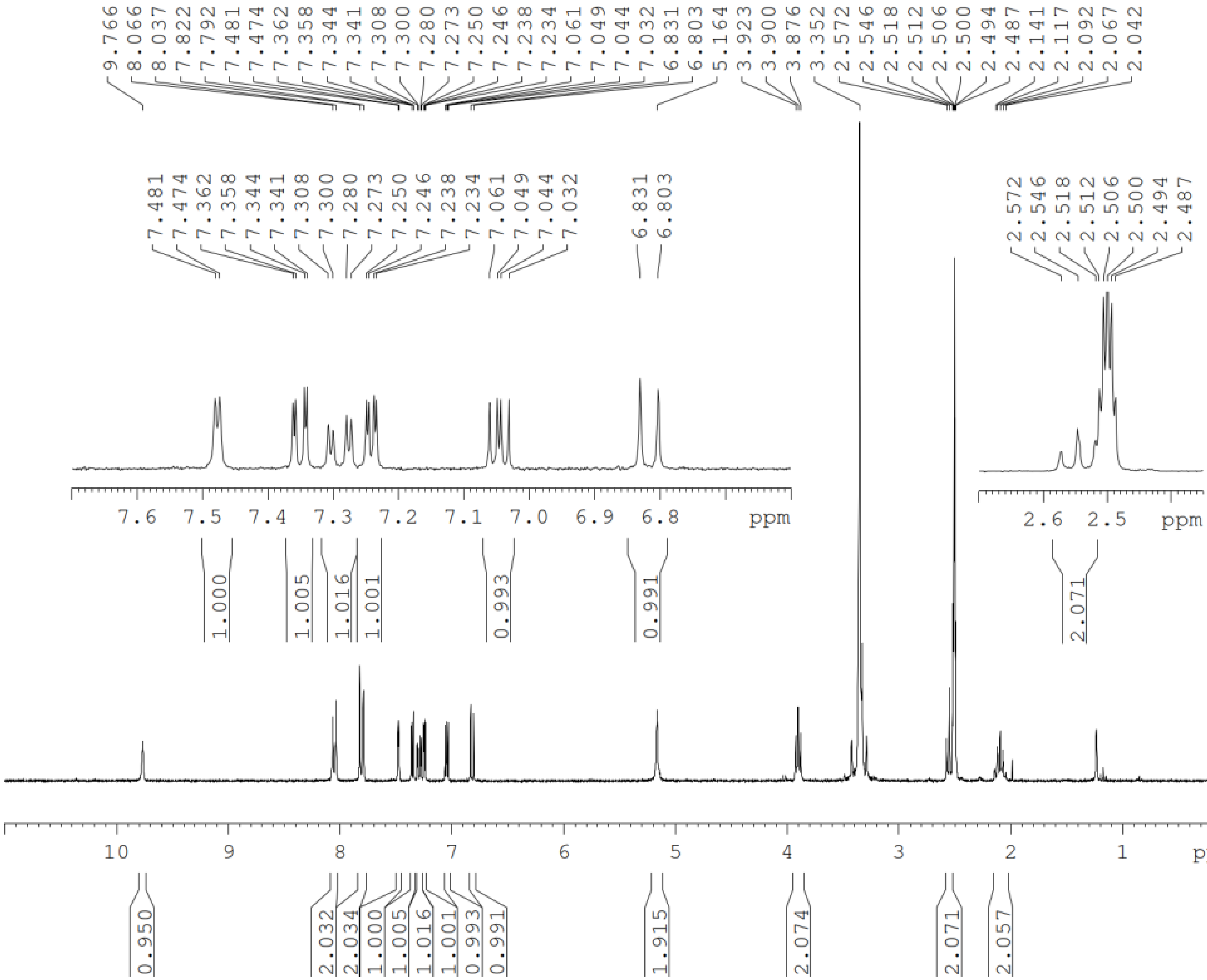

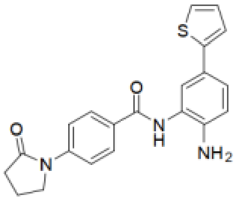

^{1}H Spectra for compound 16

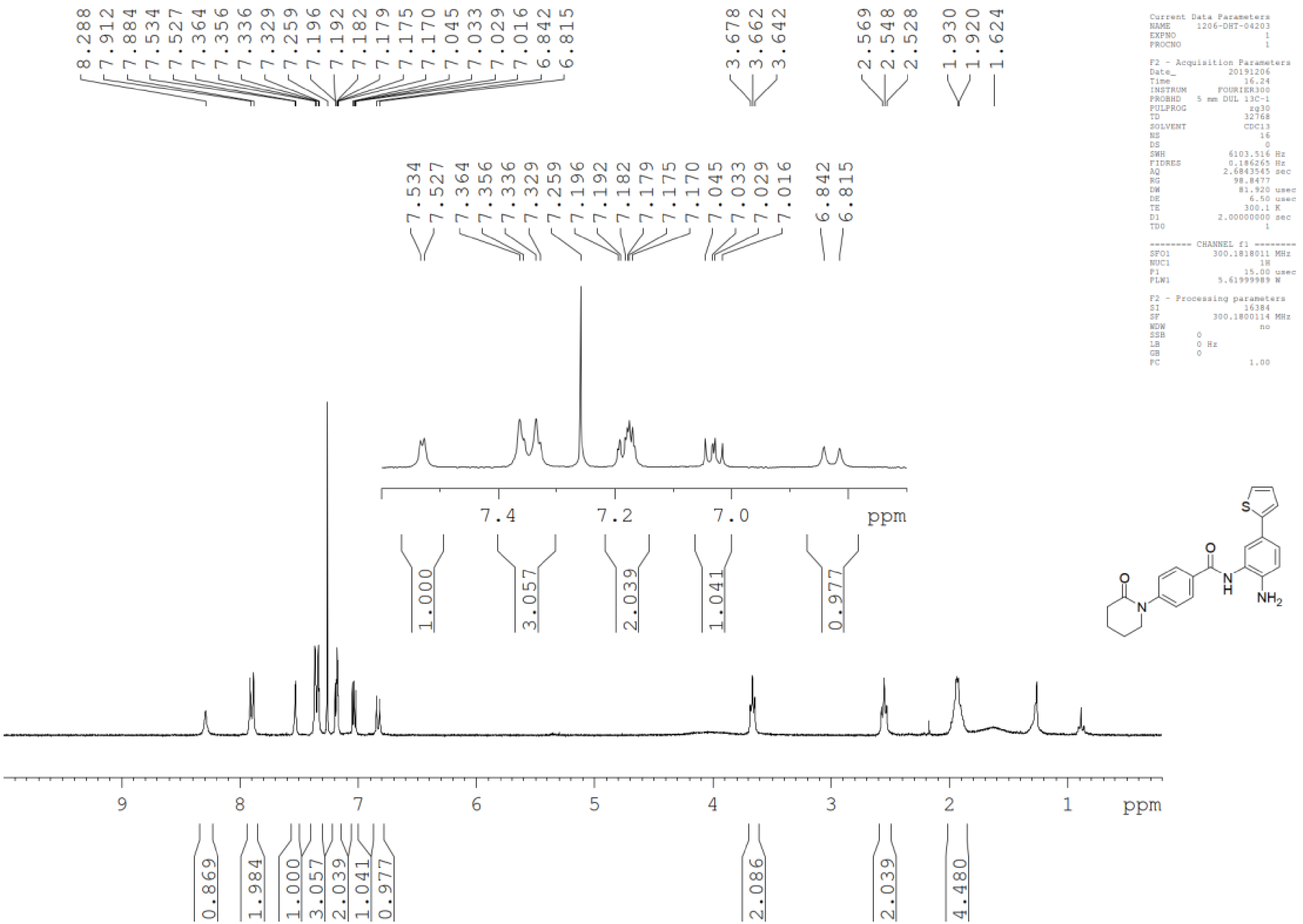

[1]H Spectra for compound 17

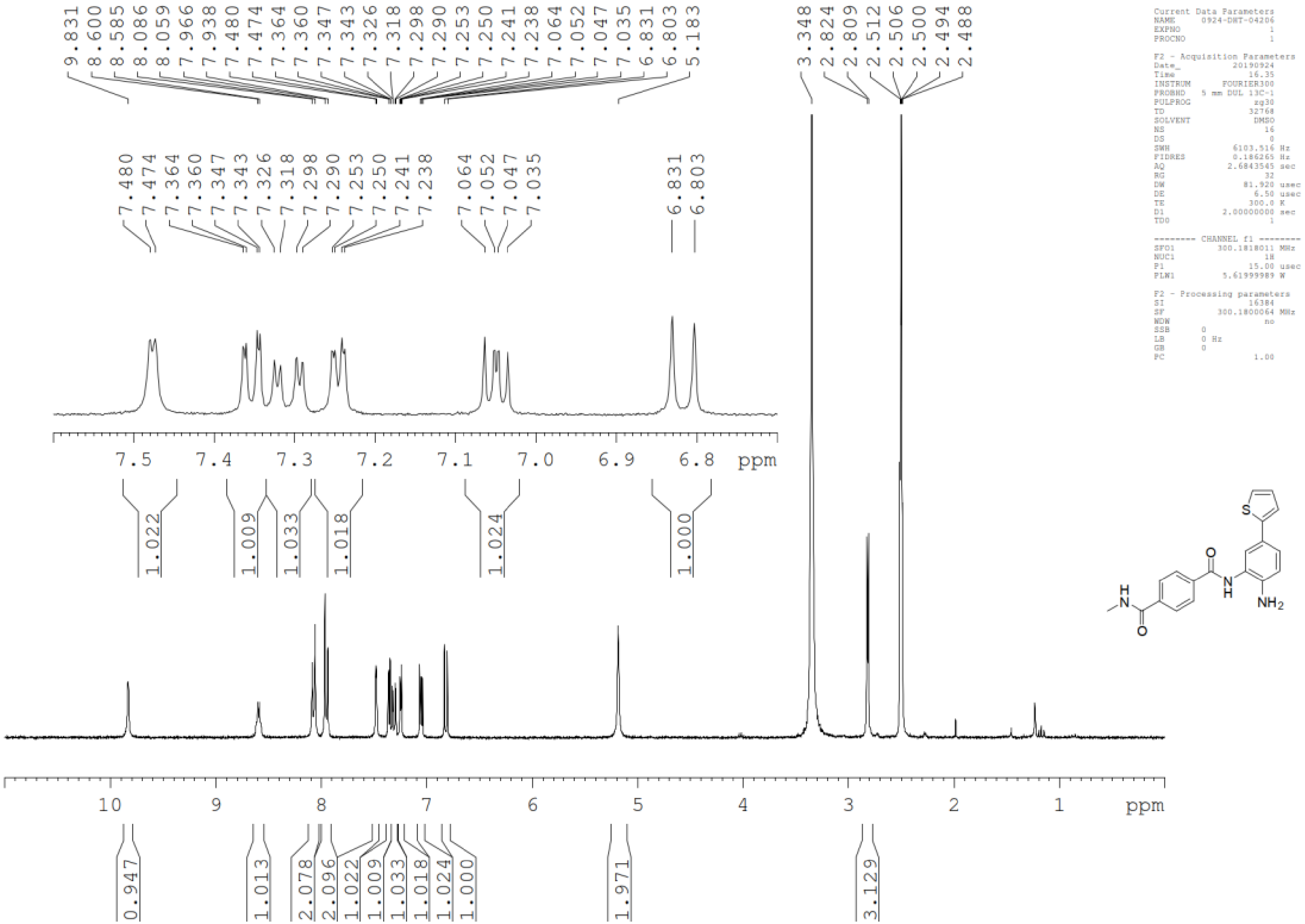

^{1}H Spectra for compound 18

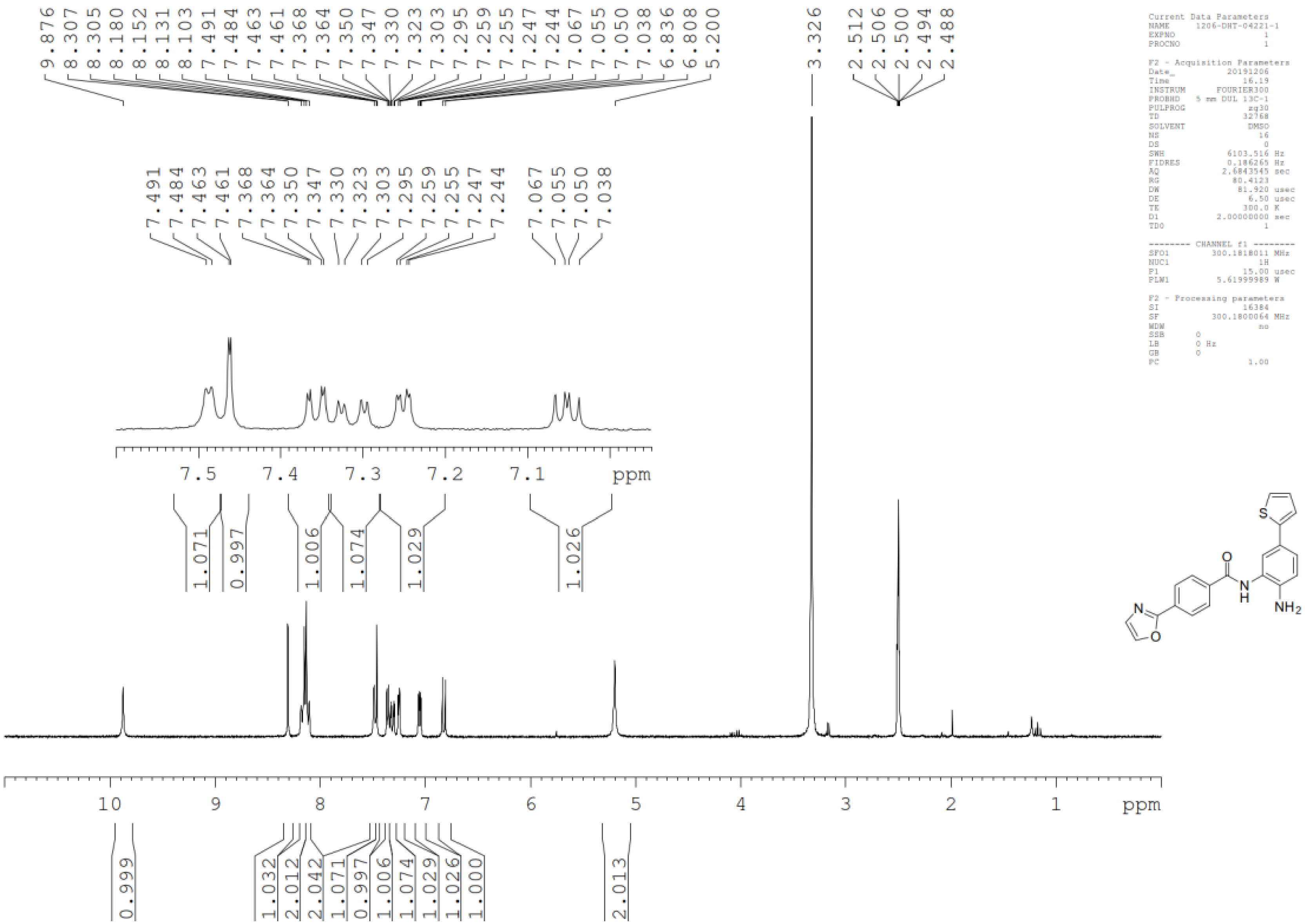

[1]H Spectra for compound 19

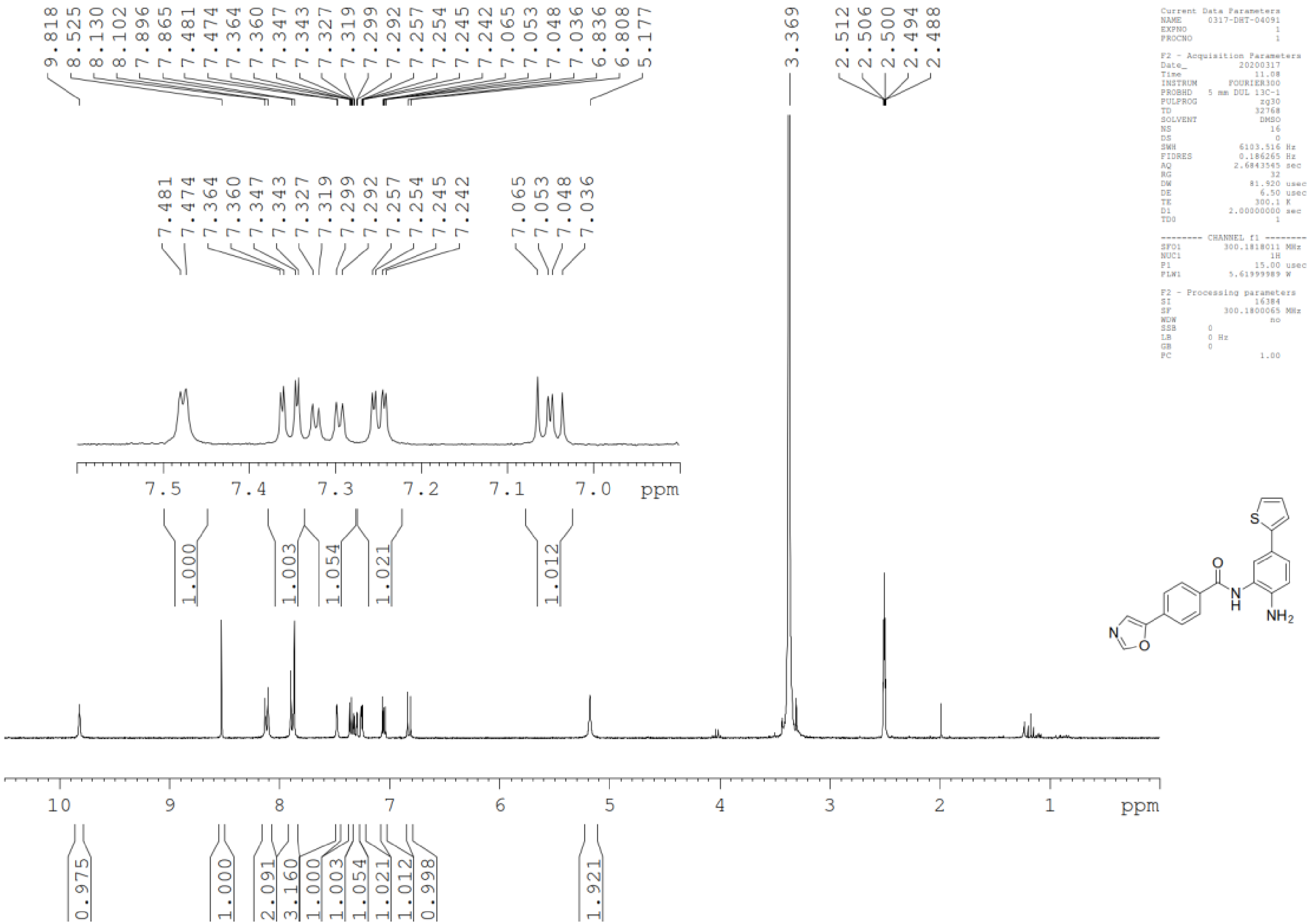

[1]H Spectra for compound 20

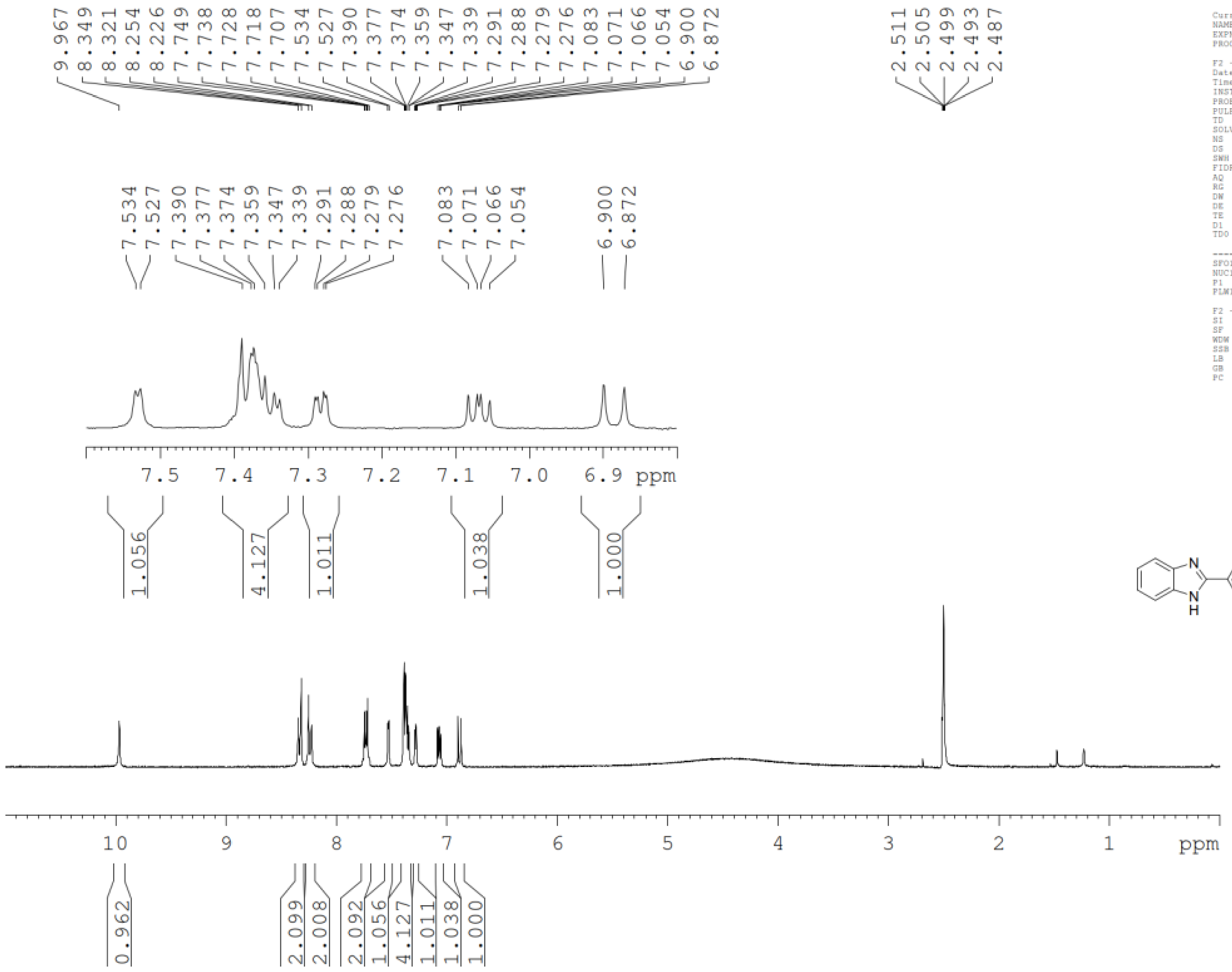

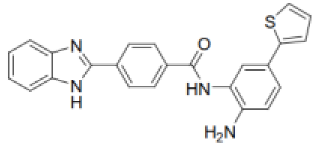

```
Current Data Parameters
NAME     0723-DHT-benzimidazole
EXPNO              1
PROCNO             1

F2 - Acquisition Parameters
Date_          20200723
Time              16.19
INSTRUM      FOURIER300
PROBHD   5 mm DUL 13C-1
PULPROG            zg30
TD                32768
SOLVENT            DMSO
NS                   16
DS                    0
SWH          6103.516 Hz
FIDRES       0.186265 Hz
AQ          2.6843545 sec
RG              56.7166
DW              81.920 usec
DE                6.50 usec
TE               300.0 K
D1         2.00000000 sec
TD0                   1

======== CHANNEL f1 ========
SFO1      300.1818011 MHz
NUC1                 1H
P1                15.00 usec
PLW1         5.61999989 W

F2 - Processing parameters
SI                16384
SF        300.1800061 MHz
WDW                  no
SSB      0
LB       0 Hz
GB       0
PC                 1.00
```

^{13}C Spectra for compound 7

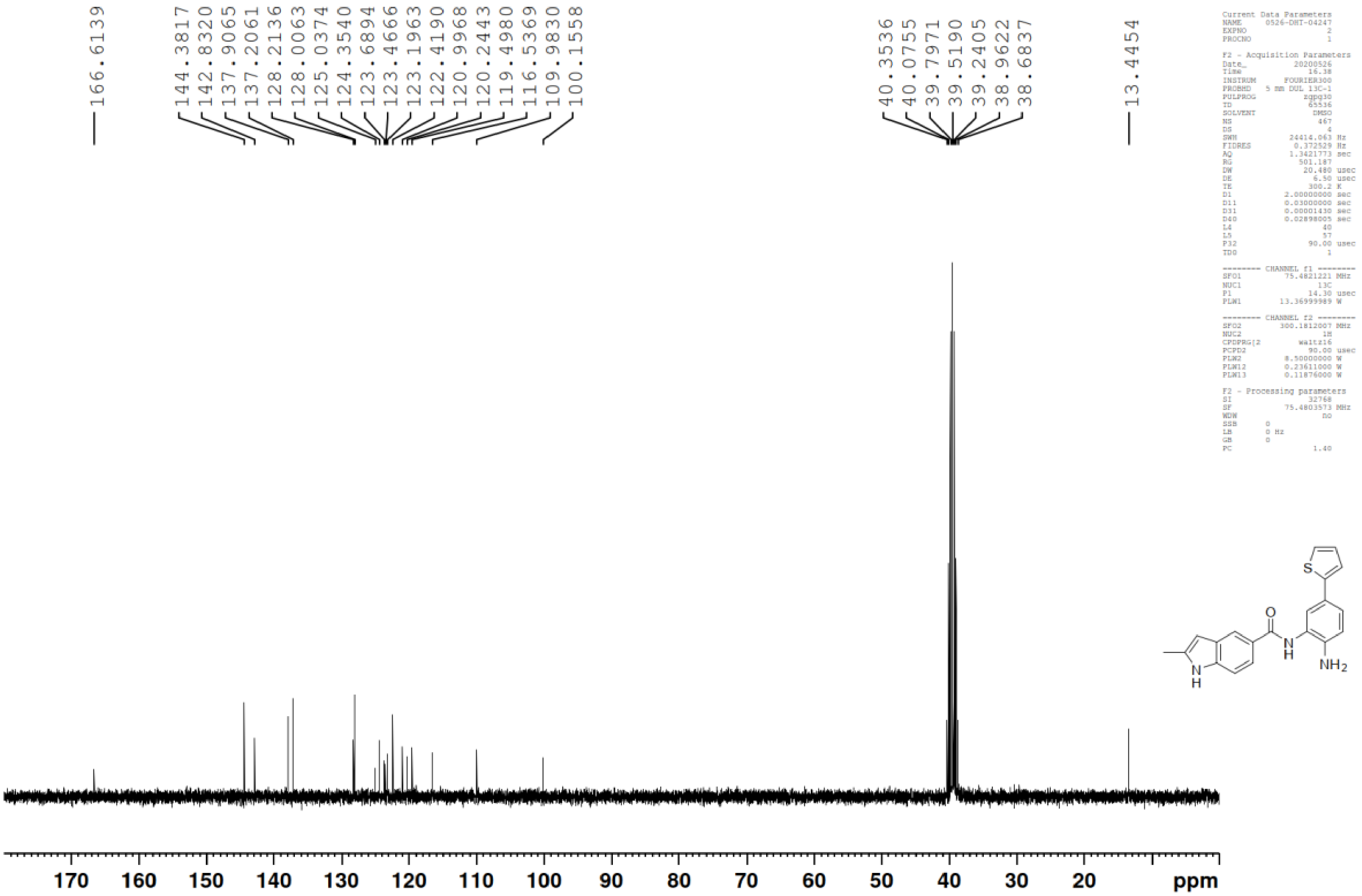

^{13}C Spectra for compound 8

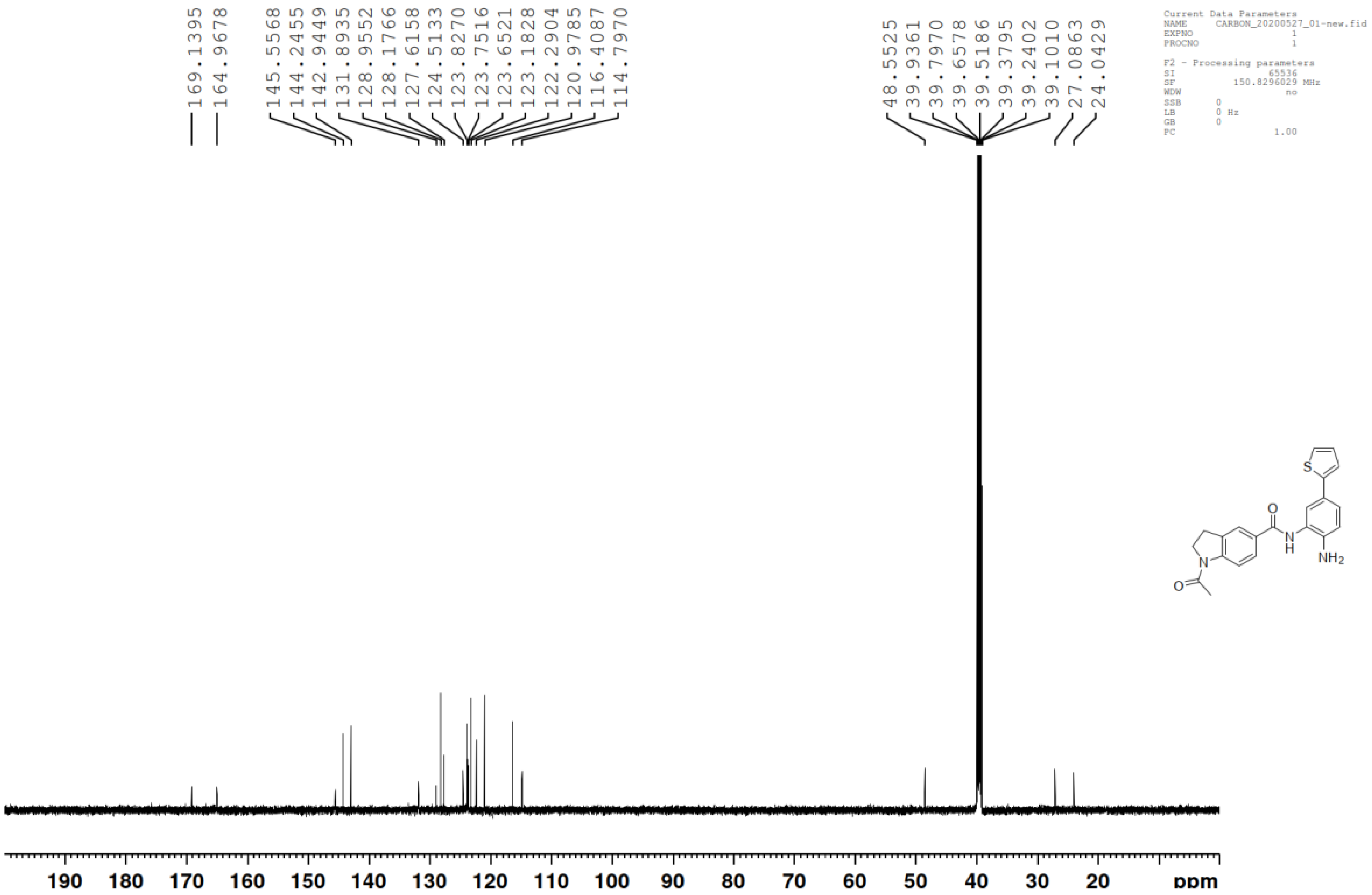

^{13}C Spectra for compound 9

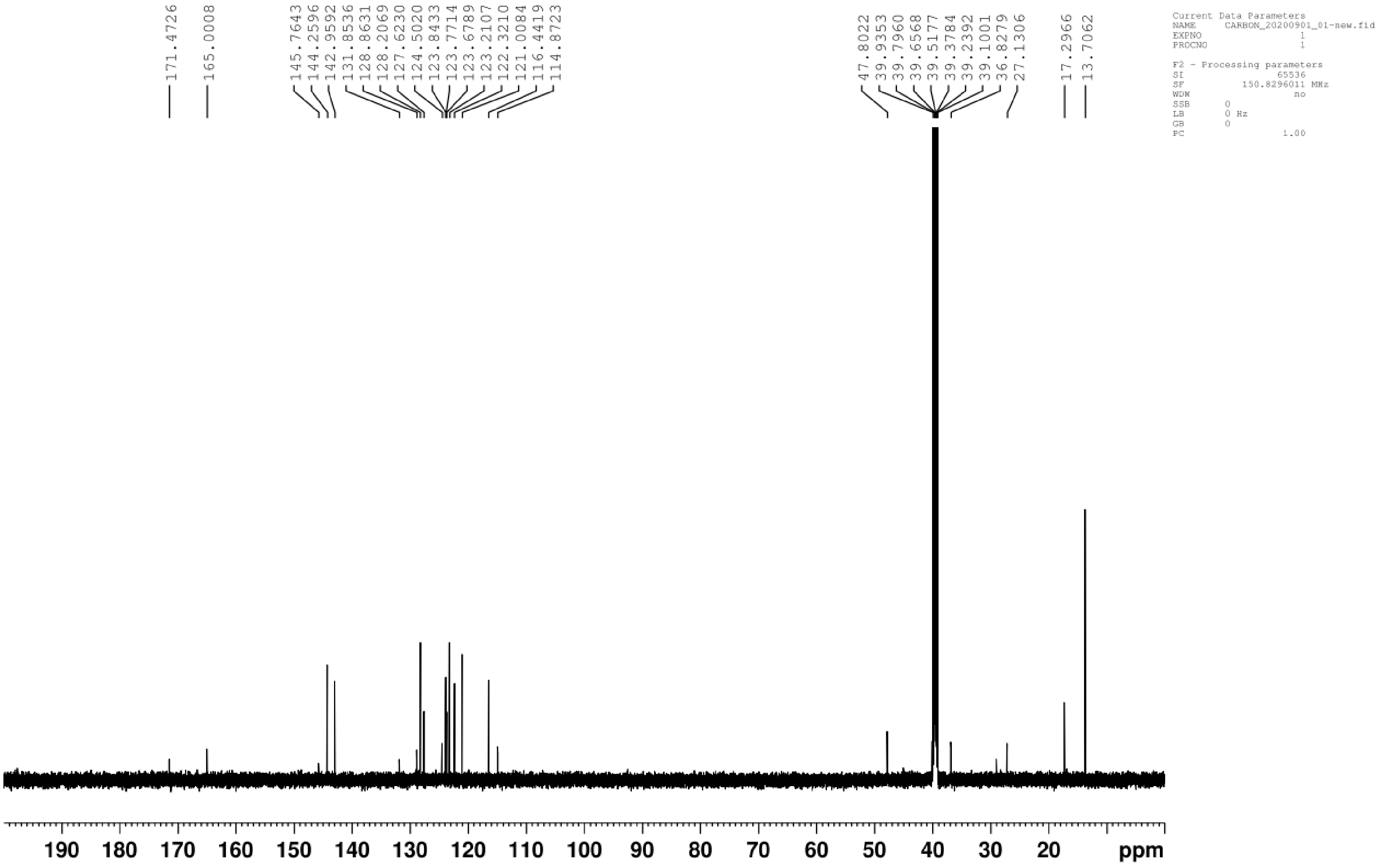

^{13}C Spectra for compound 10

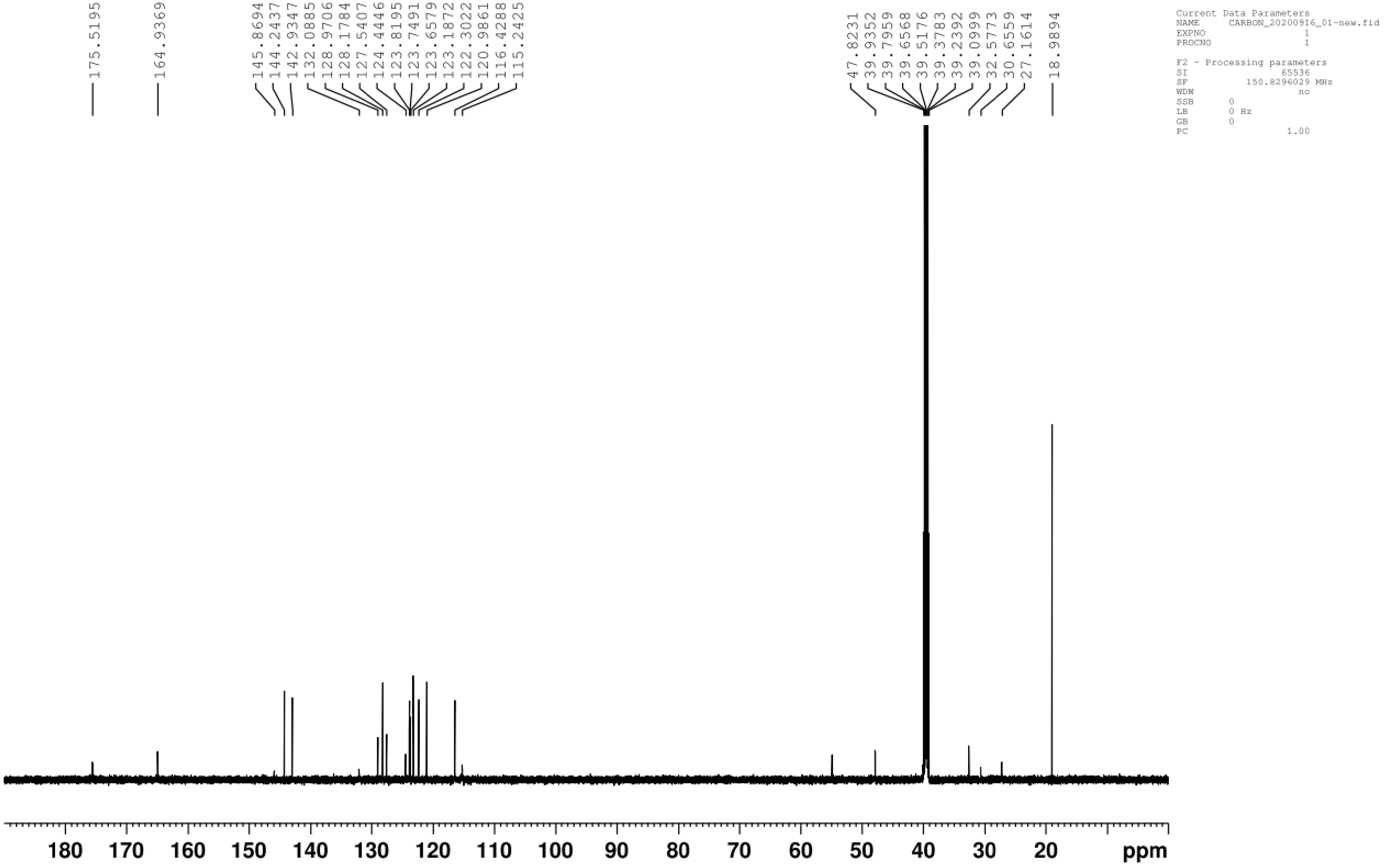

^{13}C Spectra for compound 11

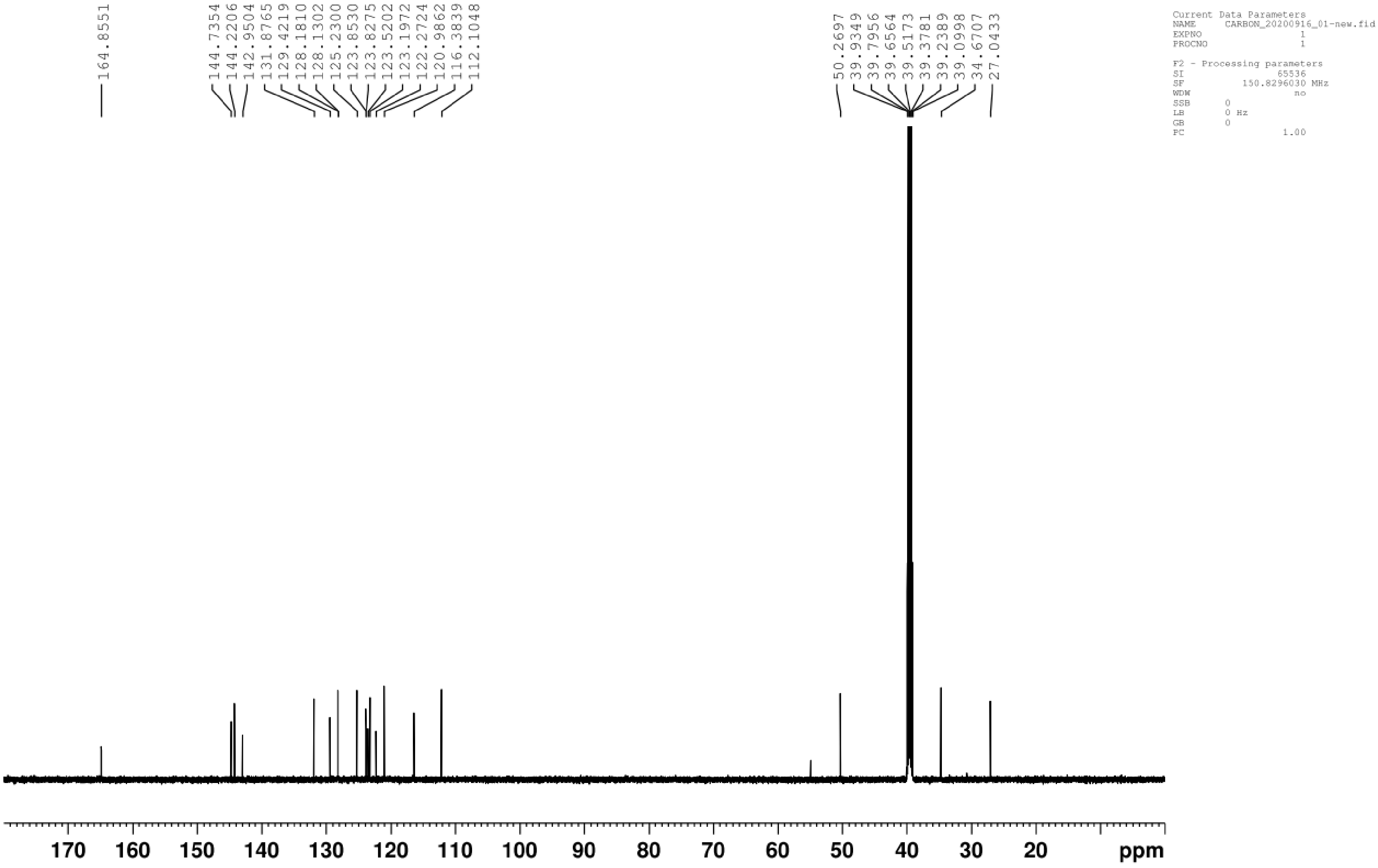

^{13}C Spectra for compound 12

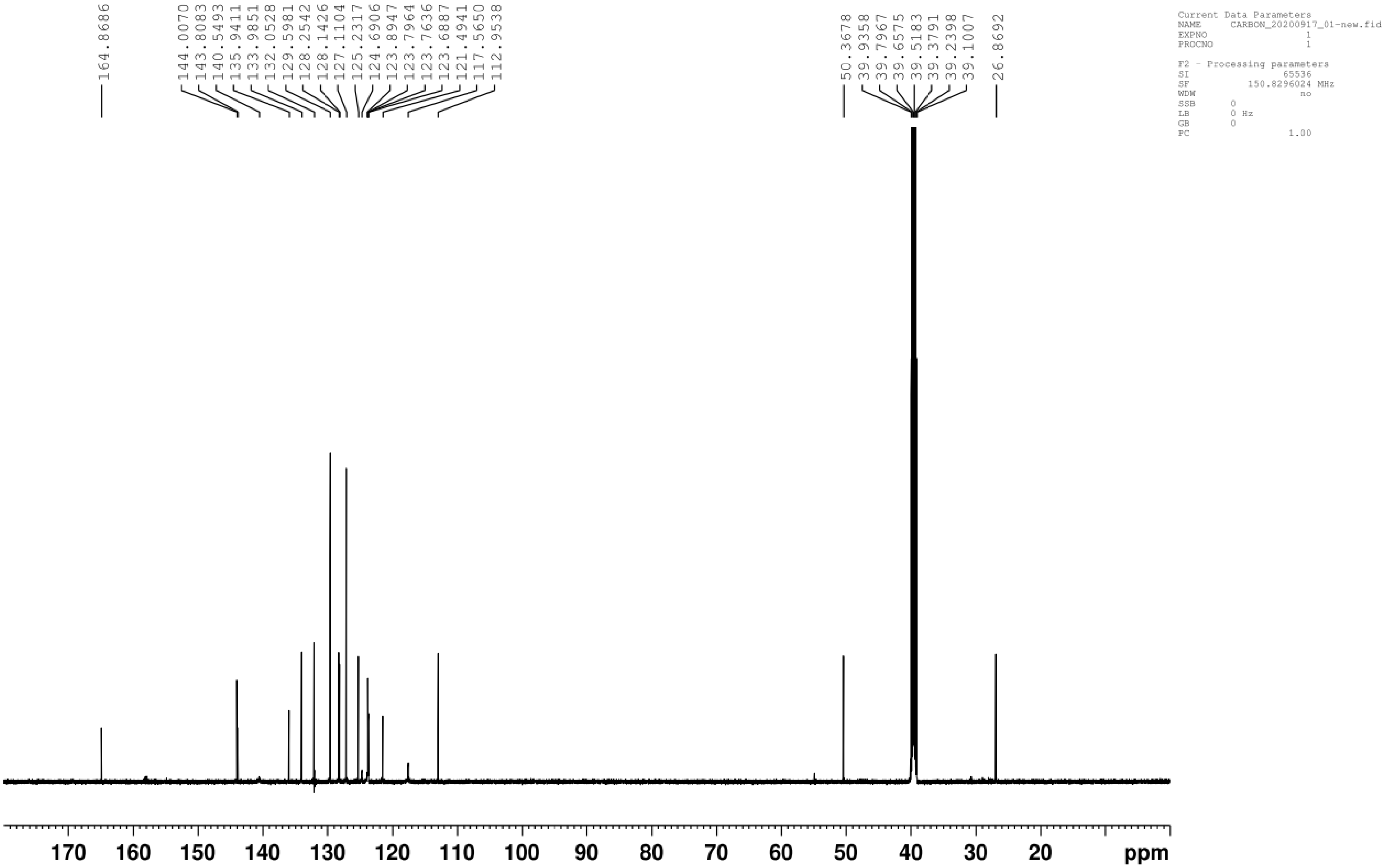

^{13}C Spectra for compound 13

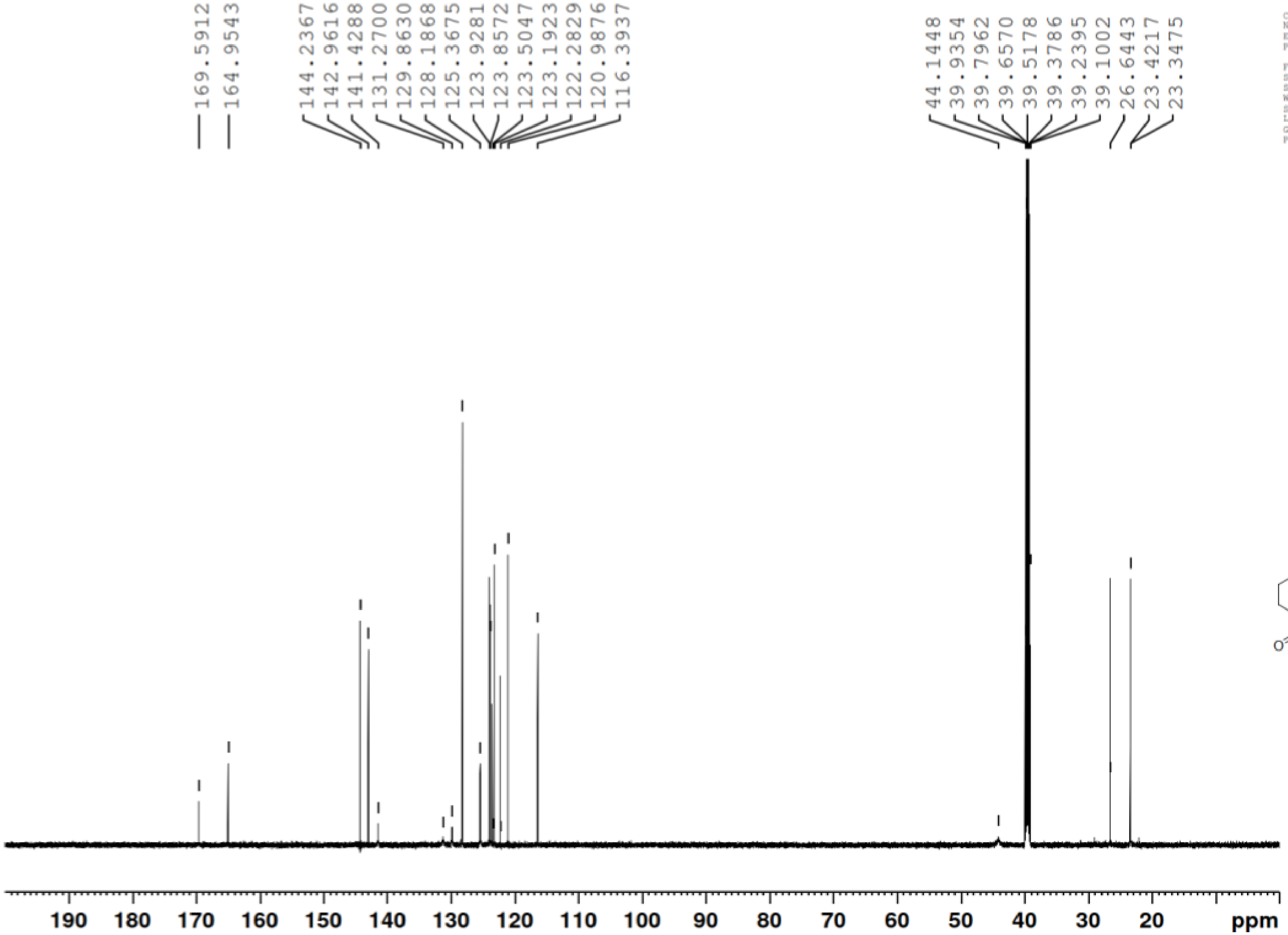

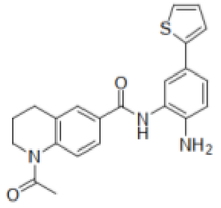

[13]C Spectra for compound 14

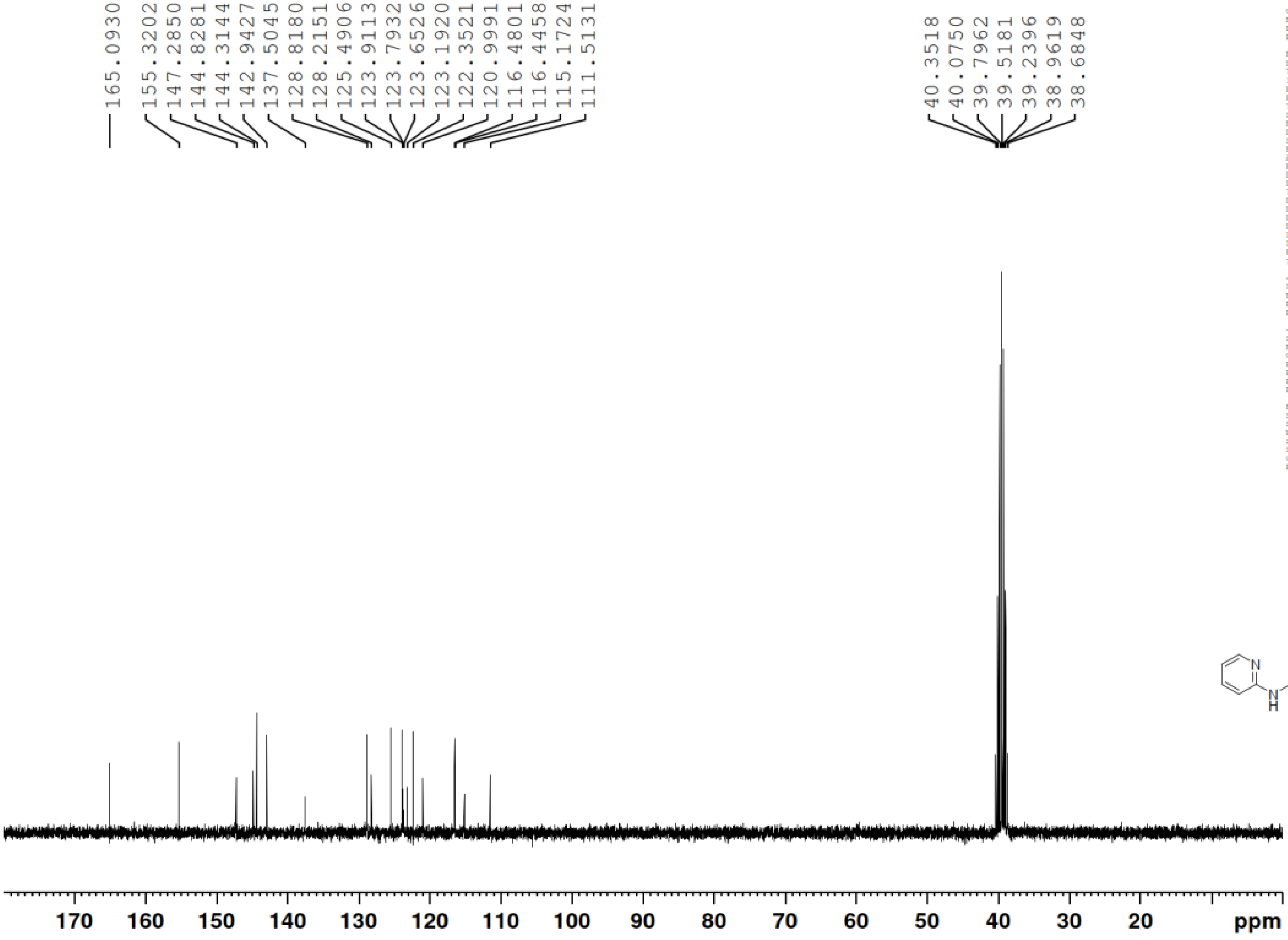

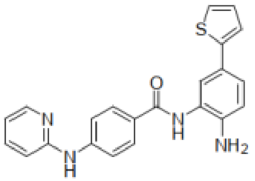

^{13}C Spectra for compound 15

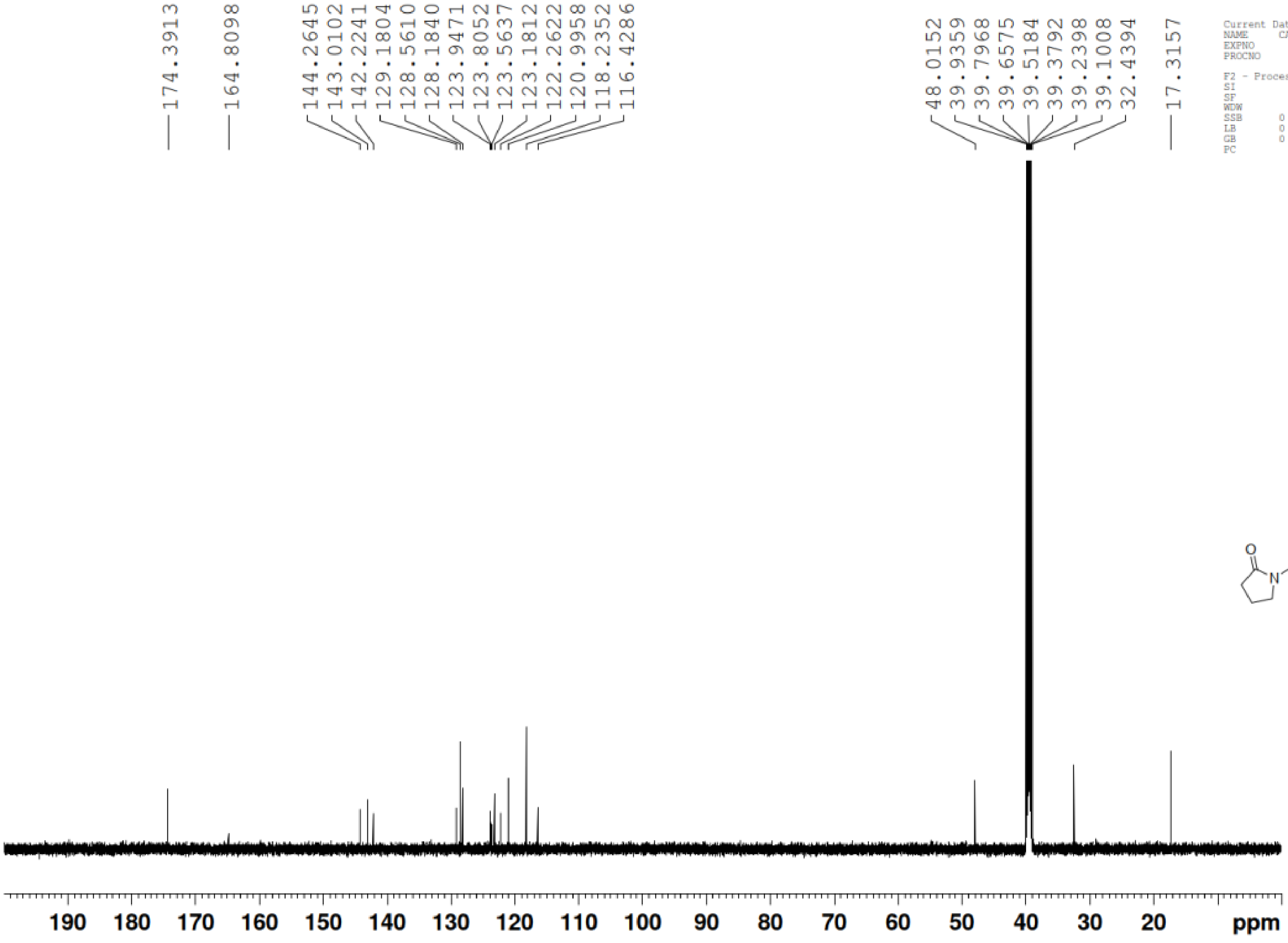

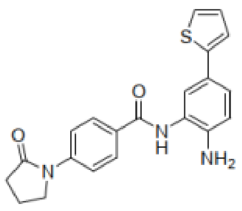

^{13}C Spectra for compound 16

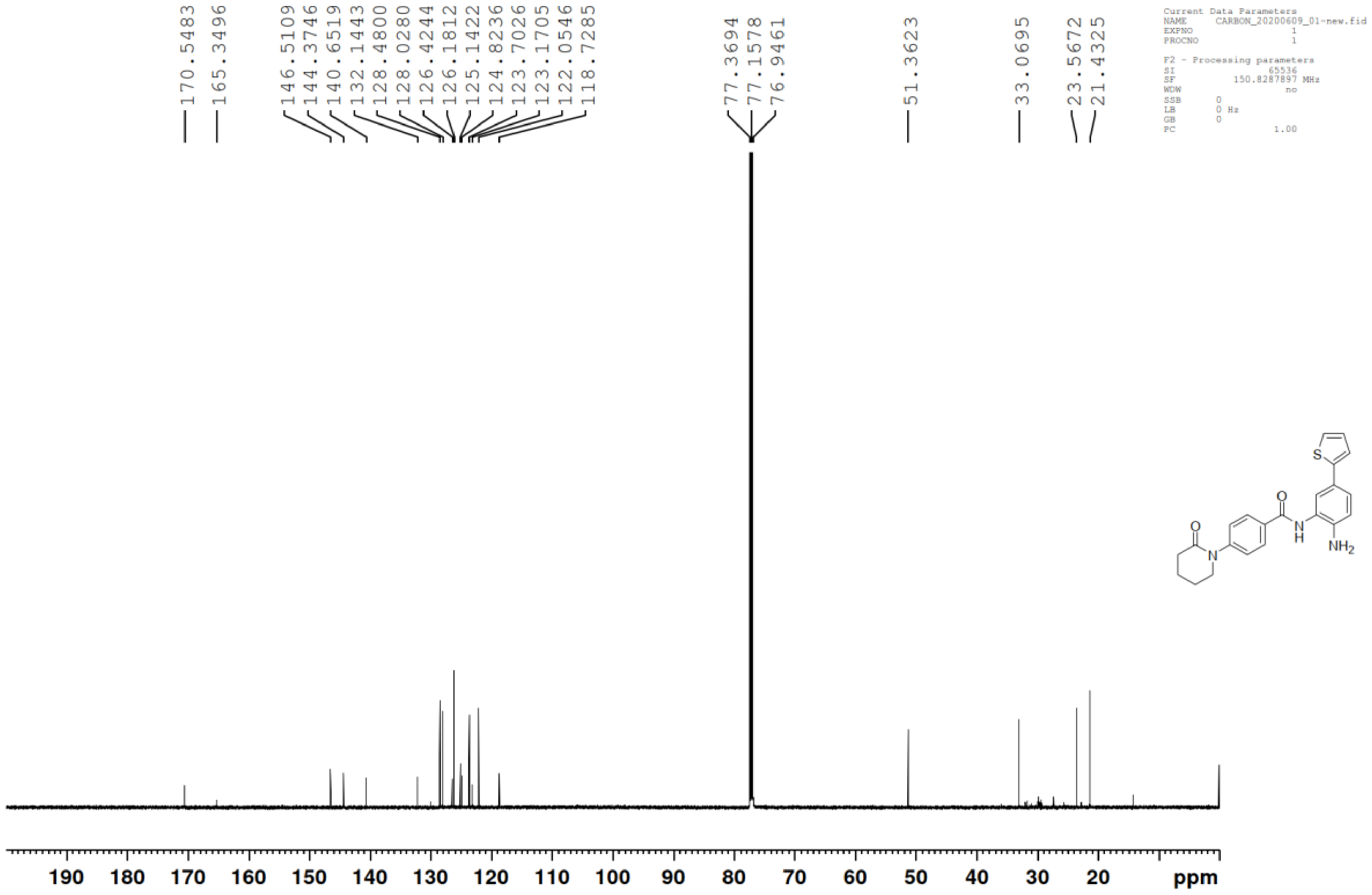

^{13}C Spectra for compound 17

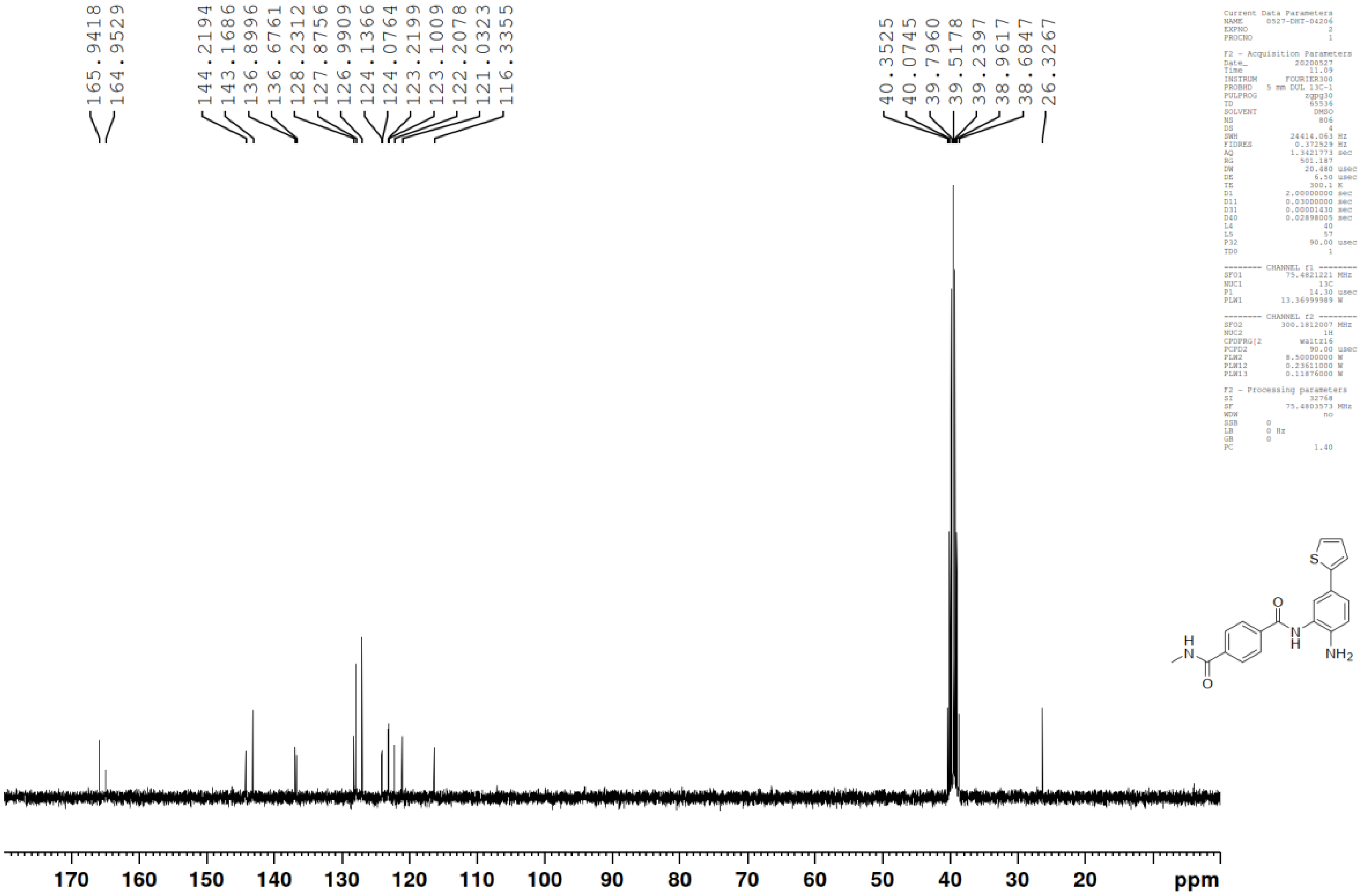

^{13}C Spectra for compound 18

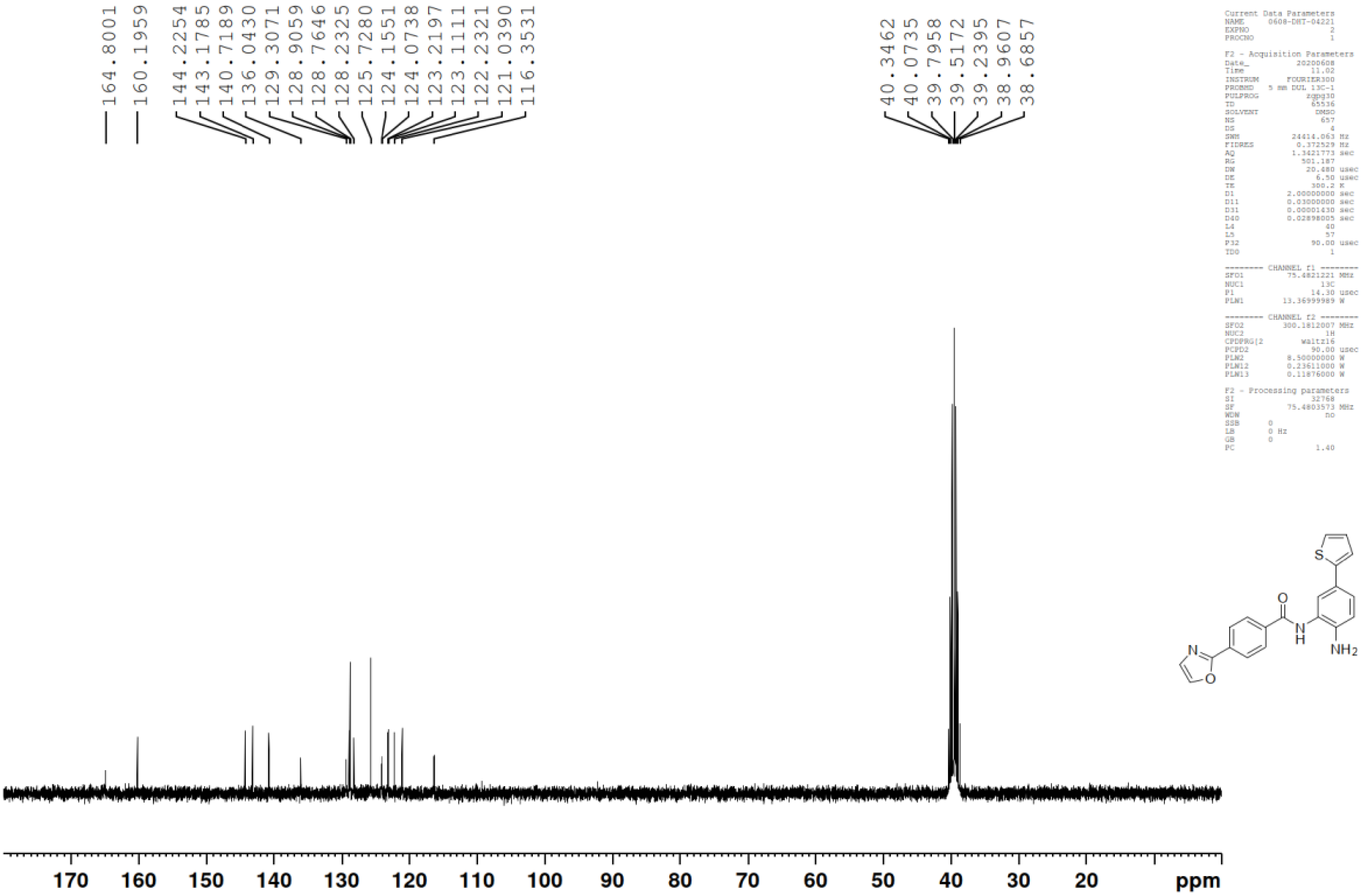

^{13}C Spectra for compound 19

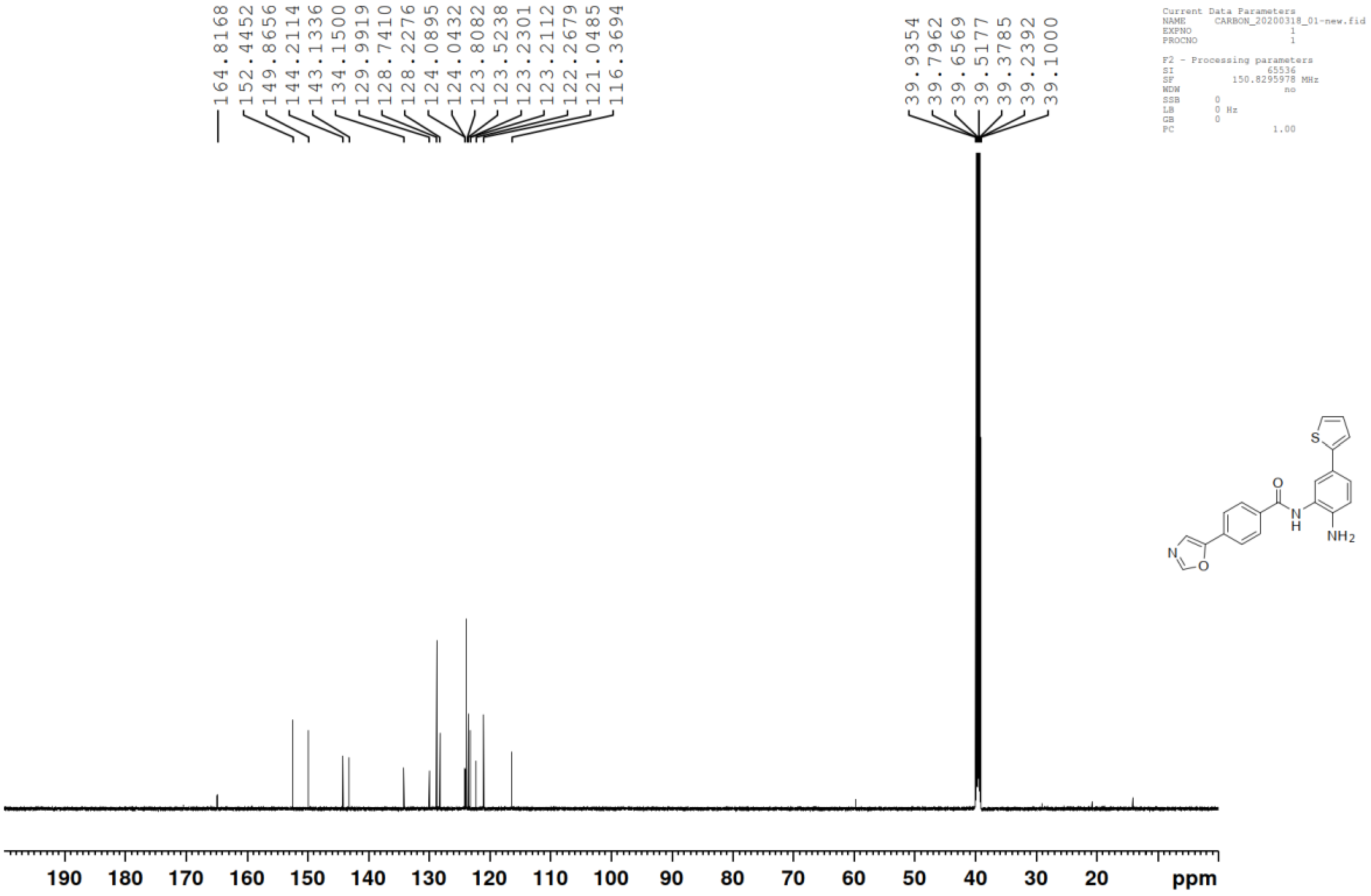

^{13}C Spectra for compound 20

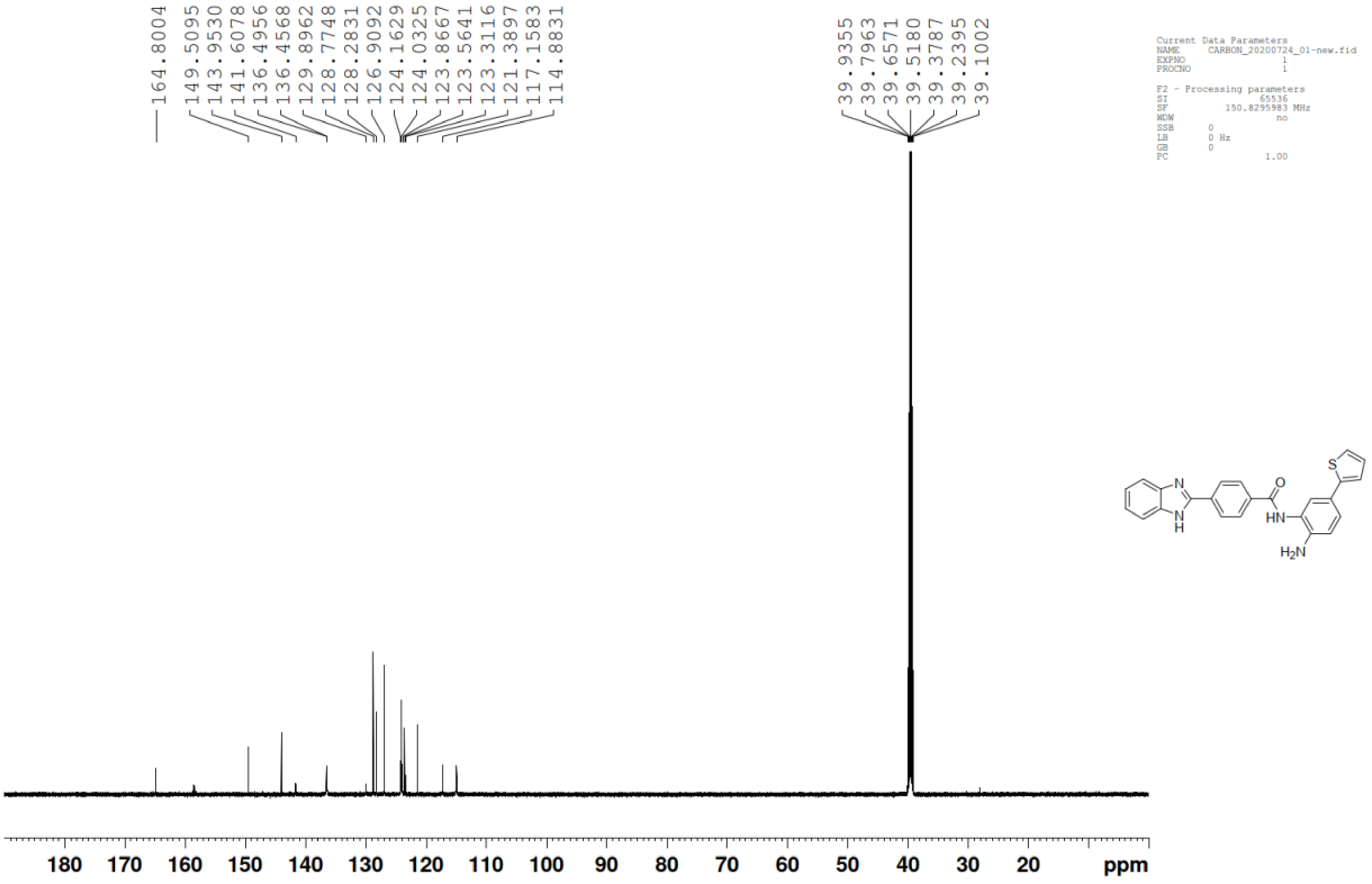

Supplement: Supplemental Material [file IENZ_A_1941920_SM3737.pdf]
